# Hydrostatic pressure-induced heritable variation in rice is associated with transposon mobilization, DNA methylation changes, and transcriptional reprogramming

**DOI:** 10.1007/s00299-026-04009-y

**Published:** 2026-10-06

**Authors:** Lanjuan Hu, Qi Wang, Yi Li, Wenzhu Jiang, Yu Zhong, Shuangzhan Huang, Zhengyu Feng, Xinru Xing, Boya Guo, Jiawei Peng, Xinglin Du, Ziming Ma

**Affiliations:** 1 Jilin Provincial Engineering Laboratory of Plant Genetic Improvement, College of Plant Science, Jilin University, Changchun, 130062 China; 2 Max-Planck-Institute of Molecular Plant Physiology, Am Muehlenberg 1, 14476 Potsdam-Golm, Germany

**Keywords:** Hydrostatic pressure, Rice (*Oryza sativa* L.), Transposable elements, DNA methylation, Transposon mobilization, Epigenetic variation, Transcriptional reprogramming

## Abstract

**Key message:**

Hydrostatic pressure treatment is associated with persistent phenotypic variation in rice, accompanied by transposon mobilization, DNA methylation changes, and transcriptional reprogramming that remain detectable after nine generations of self-pollination.

**Abstract:**

Environmental stresses can induce genetic and epigenetic changes that contribute to phenotypic variation and, in some cases, may persist across generations. However, whether hydrostatic pressure can generate long-lasting molecular and phenotypic variation in plants remains poorly understood. Here, we investigated a hydrostatic pressure-derived rice lineage (HPM) obtained from pressure-treated seeds and maintained by self-pollination for nine generations. Compared with the wild-type cultivar JL307, HPM plants showed significantly reduced plant height, tiller number, and seed-setting rate. To investigate the molecular basis of these phenotypic differences, we combined analyses of transposon mobilization, DNA methylation, and gene expression. Transposon display showed that the MITE *mPing* remained mobile, and whole-genome resequencing identified 255 candidate HPM-specific transposable element insertion sites, predominantly in the LTR retrotransposon families Dasheng and Hopi; detected DNA methylation alterations at sampled CCGG sites, in which hypomethylation predominated; and found extensive transcriptional reprogramming involving numerous stress-responsive genes and transcription factor families, including WRKY, MYB, ERF/AP2, NAC, bHLH, and VQ. These molecular changes were accompanied by phenotypic differences that remained detectable in the ninth selfed generation. These findings suggest that hydrostatic pressure treatment can be associated with persistent variation in a selfed rice lineage that remain detectable after nine generations of selfing and was accompanied by transposon mobilization, DNA methylation changes, and transcriptional reprogramming, providing a foundation for further studies of stress-associated heritable variation in plants and its potential applications in crop improvement.

**Supplementary Information:**

The online version contains supplementary material available at https://doi.org/10.1007/s00299-026-04009-y.

## Introduction

Plants frequently encounter environmental stresses that affect growth, development, and reproduction. In addition to triggering immediate physiological responses, stress exposure can induce persistent changes in cellular regulatory networks, and some stress-associated effects can be transmitted to subsequent generations (Lämke and Bäurle 2017; Herman and Sultan 2011). Such intergenerational and transgenerational responses are frequently associated with epigenetic mechanisms, including DNA methylation, histone modification, and small RNA-mediated pathways, which can alter gene expression without changing the underlying DNA sequence (Lämke and Bäurle 2017; Law and Jacobsen 2010; Zhang et al. 2018). These heritable molecular changes may contribute to phenotypic variation and environmental adaptation (Herman and Sultan 2011; Lisch 2013). However, the mechanisms by which environmental stresses generate persistent variation remain incompletely understood.

Transposable elements (TEs) are major components of plant genomes and important sources of genetic variation (Lisch 2013). In rice, TEs constitute a large fraction of the genome and have shaped genome structure and gene regulation (International Rice Genome Sequencing Project 2005; Naito et al. 2006). Under normal conditions, most TEs are maintained in a transcriptionally silent state through DNA methylation and repressive chromatin modifications (Law and Jacobsen 2010; Slotkin and Martienssen 2007). Environmental stresses can disrupt this silencing and release TEs from epigenetic control, leading to transposition and altered expression of nearby genes (Ito et al. 2011; Ito 2022; McClintock 1984), and stress-induced TE activation has been reported in a wide range of plant species (Ito 2022; Negi et al. 2016). In rice, the transposable element *mPing* has become a well-established model for environmentally and genomically induced transposition, demonstrating that environmental and genomic perturbations can stimulate TE mobilization and generate heritable genetic variation (Naito et al. 2006; Lin et al. 2006; Nakazaki et al. 2003). In addition, environmental stresses can induce changes in DNA methylation (Ma et al. 2025; Zhang et al. 2018; Lodhi and Srivastava 2025), which may contribute to stress memory and the maintenance of altered molecular states (Lämke and Bäurle 2017).

Compared with other abiotic stresses, the biological effects of hydrostatic pressure in plants remain relatively poorly studied. Hydrostatic pressure treatment has been reported to affect seed germination, seedling growth, and gene expression in rice (Liu et al. 2008; Bai et al. 2003a) and to trigger the mobilization of the transposable element *mPing* and its autonomous partner *Pong* in planta (Lin et al. 2006). Moreover, hydrostatic pressure has been explored as a physical mutagen: treatment of rice seeds has been reported to induce heritable changes in agronomic traits, such as plant height, panicle number, grain size, and fertility (Bai et al. 2003b), and pressure-induced *mPing* mobilization has been proposed as a potential tool for gene tagging (Lin et al. 2006). However, whether pressure-associated molecular changes can persist across multiple generations and contribute to persistent variation remains unknown; in particular, the possible involvement of transposon mobilization, DNA methylation reprogramming, and transcriptional regulation in hydrostatic pressure-associated inheritance is largely unexplored.

In our previous work, hydrostatic pressure treatment stimulated the mobilization of *mPing* and *Pong* in rice (Lin et al. 2006). From the same experimental system, a hydrostatic pressure-derived rice lineage (HPM) was subsequently obtained and maintained through nine generations of self-pollination, and it exhibited stable reductions in plant height and seed-setting rate. On this basis, we hypothesized that hydrostatic pressure treatment can induce persistent transposon mobilization and DNA methylation alterations that are associated with persistent phenotypic variation in rice. To test this hypothesis, we characterized the HPM lineage at the phenotypic, genomic, epigenetic, and transcriptomic levels by integrating transposon display, whole-genome resequencing, methylation-sensitive amplified polymorphism (MSAP), locus-specific bisulfite sequencing, and microarray-based expression profiling. Our findings show that long-term hydrostatic pressure-associated phenotypic variation in rice is accompanied by transposon mobilization, DNA methylation alterations, and transcriptional reprogramming, providing new insights into stress-associated heritable variation in plants.

## Materials and methods

### Plant materials and hydrostatic pressure treatment

The rice materials used in this study consisted of the *japonica* cultivar JL307 (WT) and a stable hydrostatic pressure-derived lineage (HPM lineage). The HPM lineage was derived from a population generated through hydrostatic pressure treatment of JL307 seeds (Lin et al. 2006) and was obtained as two independent eighth-generation lines, designated 1214 and 1218. Line 1214 was selected for detailed characterization because it showed both low AFLP polymorphism relative to WT (30 primer pairs: 9 polymorphic bands out of 1603 scorable bands, 0.56%) and excision of *mPing* at multiple insertion sites, whereas no such excisions were detected at the corresponding sites in line 1218, which was, therefore, not analyzed further. Excision of *mPing* was monitored during maintenance of the lineage by locus-specific PCR using primers designed from the flanking sequences of known insertion sites (a PCR product approximately 430 bp shorter than the corresponding product containing the element indicates excision); these screening data are not shown, and plants showing excision events were advanced to the next generation. The selected line was self-pollinated for one additional generation, and plants of the ninth generation were used for all analyses. The HPM lineage exhibited stable phenotypic alterations, including reduced plant height, decreased tiller number, and lower seed-setting rate compared with the wild type.

Hydrostatic pressure treatment of JL307 seeds was performed as described in our previous study (Lin et al. 2006): seeds soaked in distilled water at room temperature for 24 h were subjected to continuous high hydrostatic pressure. The HPM lineage analyzed in this study descends from seeds treated at 100 MPa for 15 min at room temperature (Lin et al. 2006; Liu et al. 2008). Following treatment, germinated seeds were transplanted into a conventional paddy field. Plants showing altered phenotypes were advanced through continuous self-pollination, and the HPM lineage was maintained for nine generations. The ninth-generation HPM lineage (HPM) and the corresponding wild-type JL307 were used for all analyses in the present study.

For molecular analyses, leaf tissues were collected from seedlings at the two-leaf stage and immediately frozen in liquid nitrogen prior to DNA and RNA extraction.

### Phenotypic evaluation

To evaluate phenotypic variation between WT and HPM, plant height, tiller number, and seed-setting rate were measured under field conditions at the mature stage. Plants were grown in a conventional paddy field at the Rice Research Institute, Jilin Academy of Agricultural Sciences, Gongzhuling, Jilin Province, China, in the 2011 growing season; rows of WT and HPM (10 plants per row) were interleaved alternately within the same field, and all plants were managed under the standard local agronomic practices for japonica rice, applied uniformly to both genotypes. Plant height was determined as the distance from the soil surface to the tip of the tallest panicle (excluding awns) and was measured on 80 plants per genotype. Tiller number was recorded from 82 plants per genotype. Seed-setting rate was calculated as the ratio of filled grains to total spikelets and was determined from five representative panicles per plant, averaged over 107 WT plants and 84 HPM plants. The number of plants assessed differed among traits and between genotypes because each trait was measured independently on the plants that could be reliably assessed for that trait; all recorded data are reported without modification. Data are presented as mean ± standard deviation (SD), and medians and interquartile ranges are additionally provided for seed-setting rate. Differences between WT and HPM were evaluated using Welch’s t-test for plant height, tiller number, and seed-setting rate. Because the distribution of seed-setting rate deviated substantially from normality and variances were unequal between the genotypes (Levene’s test, *P* < 0.001), differences in seed-setting rate were additionally evaluated using the non-parametric Mann–Whitney U test, and 95% confidence intervals for the difference in means were estimated by bootstrap resampling (10,000 iterations). Statistical significance was defined as *P* < 0.05.

### Transposon display analysis

Transposon activity was evaluated using transposon display (TD) analysis following previously described procedures with minor modifications (Casa et al. 2000; Miyao et al. 2003). Genomic DNA was extracted from WT and four individual plants of the ninth-generation HPM lineage; the same four plants were used for both transposon display and MSAP analyses. Approximately 300 ng of genomic DNA was digested with *Mse*I and ligated to *Mse*I adapters, and selective amplification was performed using transposon-specific primers targeting the MITE family *mPing*, an element previously shown to be mobilized by hydrostatic pressure treatment in this experimental system (Lin et al. 2006); the 29 selective primer combinations adopted from (Lin et al. 2006) are listed in Supplementary Table S1. Amplification products were separated on 6% polyacrylamide gels, and only clear and reproducible bands were scored. Bands present in HPM, but absent in WT were considered putative transposon insertions, whereas bands present in WT, but absent in HPM were considered putative excision events. The transposon polymorphism rate was calculated as the number of polymorphic bands divided by the total number of scored bands (Supplementary Table S2).

### Whole-genome resequencing and identification of transposable element insertions

Whole-genome resequencing was performed to investigate genomic variation and identify transposable element (TE) insertion events in HPM. Genomic DNA extracted from WT (JL307) and HPM plants, with three biological replicates per genotype (each pooled from ten plants), was sequenced on the Illumina HiSeq 2000 platform with 90-bp paired-end reads, yielding average depths of approximately 13.2× (WT) and 21.7× (HPM). Clean reads were aligned to the rice reference genome (MSU Rice Genome Annotation Project Release 7.0; Nipponbare) using the Burrows–Wheeler Aligner (BWA; Li and Durbin 2009). To enable genotype-specific TE detection, a WT-informed reference genome was constructed by integrating sequence variants identified from the WT reads into the Nipponbare reference assembly using custom Perl scripts. TE insertions in HPM were detected by a paired-end mapping (PEM) strategy (Sabot et al. 2011): reads that could not be aligned to the reference genome were aligned with Bowtie to a collection of known rice transposable elements (which did not include the MITE *mPing*, whose mobility was assessed independently by transposon display), and candidate insertion sites were identified from discordant paired-end alignments in which one read mapped to a TE sequence and its paired read mapped uniquely to the reference genome. Candidates showing a clear paired-end mapping pattern on both the chromosomal region and the TE sequence were retained and filtered using SAMtools (v0.1.5c) with a Phred score ≥ 30 and coverage ≥ 10 and ≤ 100. The genomic positions of candidate insertion events were determined from the mapped locations of the paired-end reads, and TE families were classified based on sequence similarity to annotated rice transposable elements.

### Methylation-sensitive amplified polymorphism (MSAP) analysis

DNA methylation variation between WT and HPM was evaluated using methylation-sensitive amplified polymorphism (MSAP) analysis as previously described by Reyna-López et al. (1997), with minor modifications. Approximately 300 ng of genomic DNA was digested in parallel using the methylation-sensitive restriction enzymes *Hpa*II and *Msp*I in combination with *Eco*RI, ligated to corresponding adapters, and subjected to pre-selective and selective PCR amplification using 20 primer combinations. Amplification products were separated on 6% polyacrylamide gels, and only clear and reproducible bands were scored. Banding patterns were categorized following the conventional four-type MSAP scoring system (type I, both *Hpa*II and *Msp*I products present, unmethylated; type II, *Msp*I product only, CG-methylated; type III, *Hpa*II product only, CHG-methylated; type IV, neither product present); per-sample numbers of fragments in each type are provided in Supplementary Table S3. CG-related changes were scored from *Hpa*II band gains (hypomethylation) and losses (hypermethylation), and CHG-related changes from *Msp*I band gains and losses; the denominator for every reported percentage was the total number of scored fragments (1189). The complete raw banding scores are provided in Supplementary Table S4.

### Locus-specific bisulfite sequencing

To investigate the relationship between DNA methylation and gene expression, four genes showing increased transcript abundance in HPM based on microarray analysis were selected for locus-specific bisulfite sequencing. Genomic DNA extracted from two-leaf-stage seedlings of WT and HPM plants (pooled from ten plants per genotype) was treated with sodium bisulfite using the EZ DNA Methylation-Gold Kit (Zymo Research, USA) according to the manufacturer’s instructions. Bisulfite-converted DNA was used as the template for PCR amplification of selected promoter regions, using gene-specific primers listed in Supplementary Table S5. PCR products were cloned into a TA cloning vector and sequenced. Cytosine methylation levels were determined for CG, CHG, and CHH sequence contexts, and the methylation percentage was calculated as the proportion of methylated cytosines relative to the total number of cytosine sites analyzed within the amplified region. Bisulfite sequencing data were analyzed using CyMATE software (Hetzl et al. 2007). For each gene, 11–15 independent clones were sequenced per genotype, and the numbers of cytosine positions assessed were 31, 80, 62, and 71 for *LOC_Os03g08320*, *LOC_Os06g48870*, *LOC_Os02g48770*, and *LOC_Os07g48710*, respectively; the amplified promoter fragments (253, 470, 350, and 329 bp) were located upstream of the transcription start sites (TSS set as position 0). Overall and context-specific (CG, CHG, CHH) methylation levels were compared between WT and HPM for each gene, and representative methylation maps were generated using CyMATE. Bisulfite conversion efficiency was not assessed with spike-in standards; the manufacturer’s protocol was followed. The 11–15 clones per genotype were derived from a single pooled DNA preparation and, therefore, represent within-sample clonal sampling rather than independent biological replicates. Differences in methylation at individual cytosine positions were evaluated between WT and HPM using Fisher’s exact test (methylated vs unmethylated clones at each position); the numbers of significantly different positions are reported in Section. "DNA methylation alterations and their association with gene expression changes in HPM".

### Microarray analysis

Total RNA was extracted from WT and HPM seedlings using standard procedures; three independent biological replicates were analyzed for each genotype, with each replicate consisting of an independent RNA pool prepared from ten two-leaf-stage seedlings. RNA quality and concentration were assessed prior to microarray hybridization. Gene expression profiling was performed using the Affymetrix GeneChip Rice Genome Array at Gene Company Ltd. (Shanghai, China) according to the manufacturer’s instructions. Following hybridization and washing, microarrays were scanned using an Affymetrix GeneChip Scanner, and image data were processed using GeneChip Operating Software to generate CEL files. Raw intensity data were normalized using the Robust Multichip Average (RMA) algorithm (Irizarry et al. 2003). All six arrays passed the Affymetrix quality-control criteria, and the per-array quality metrics are provided in Supplementary Table S6; principal component analysis and sample correlation plots are provided in Supplementary Figure S1. Differential expression was assessed by one-way ANOVA (three biological replicates per genotype) followed by the pairwise comparison of HPM vs WT; Probes with a fold change ≥ 2 and an unadjusted *P* < 0.05 were retained as candidate differentially expressed probes. Benjamini–Hochberg correction was additionally applied to the *P* values of all 57,381 probes on the array; 305 of the candidate probes remained significant at a false discovery rate (FDR) < 0.05, and 10 at FDR < 0.01. The FDR value for each candidate probe is provided in Supplementary Table S10. The raw CEL files have been deposited in the Gene Expression Omnibus (GEO) under accession GSE345727 Gene annotations were obtained from the MSU Rice Genome Annotation Project (Release 7.0). Differentially expressed genes were subsequently classified according to their annotated functions, and transcription factor families were identified based on gene annotation information.

To validate the microarray results, the expression of 12 selected genes was examined by quantitative RT-PCR using GAPDH as the reference gene; relative expression levels were calculated by the 2^−ΔΔ*ct* method from three biological replicates and are shown as mean ± SD. The qRT-PCR primers are listed in Supplementary Table *S7*.

## Results

### Phenotypic alterations in the hydrostatic pressure-derived progeny

**Fig. 1:**
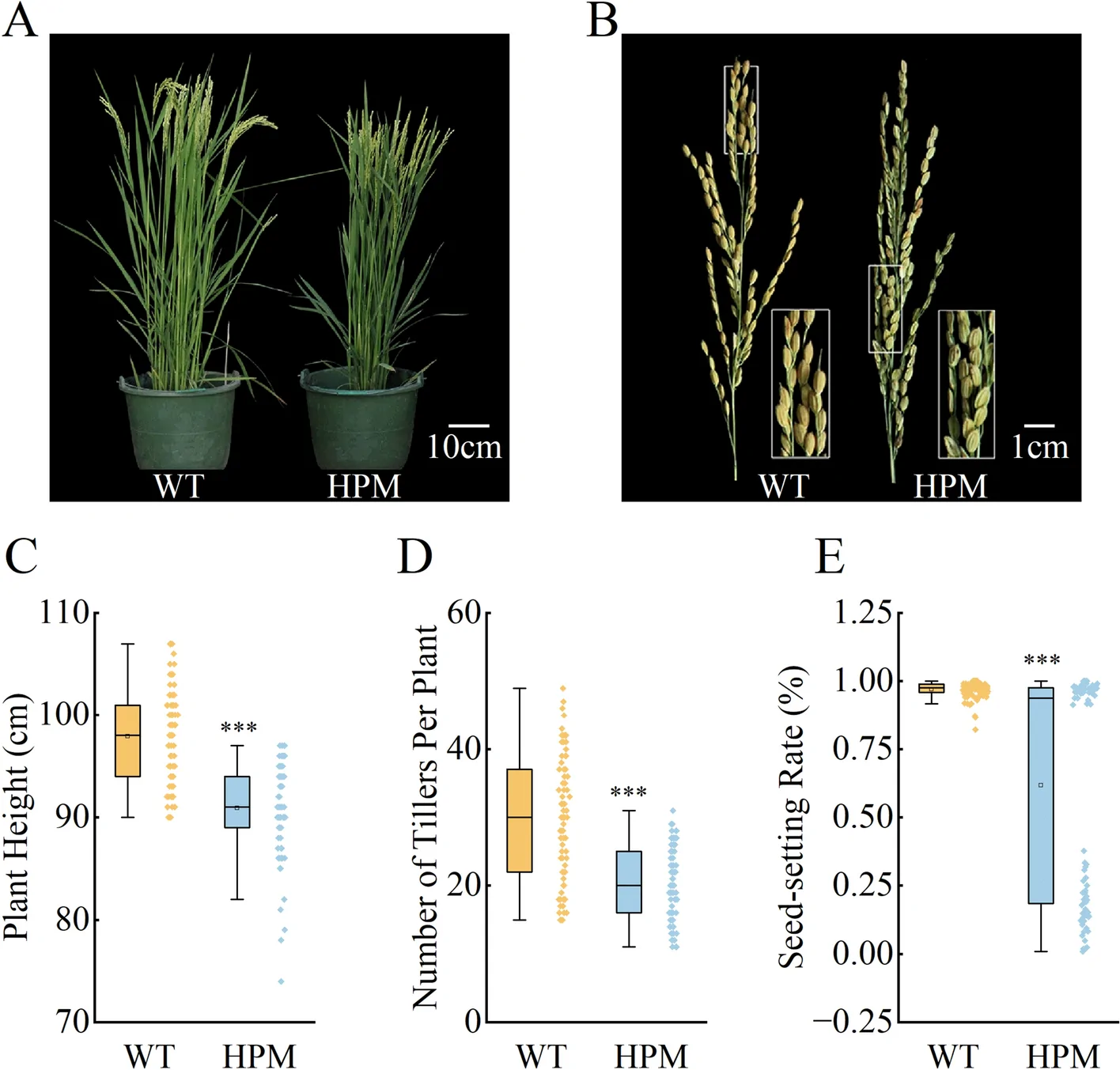
Phenotypic characterization of the HPM lineage. **A** Representative plants of WT and HPM at the heading stage. **B** Comparison of panicles and enlarged views showing seed-setting differences between WT and HPM. **C** Plant height, **D** tiller number per plant, and **E** seed-setting rate of WT and HPM. Plant height was measured on 80 plants per genotype, tiller number was recorded on 82 plants per genotype, and seed-setting rate was determined from 107 WT plants and 84 HPM plants (five representative panicles per plant). Box plots show the median, interquartile range, and outliers; individual data points are shown as overlaid dots. ***P < 0.001 (Welch’s t-test). For seed-setting rate (**E**), the non-parametric Mann–Whitney U test also yielded *P* = 3.56 × 10⁻⁸. *WT* wild type, *HPM* hydrostatic pressure-derived lineage

Phenotypic characteristics of the hydrostatic pressure-derived lineage (HPM) were compared with those of the wild-type cultivar JL307 (WT). Distinct morphological differences were observed between the two lineages at the heading stage (Fig. 1A). HPM plants exhibited a visibly reduced stature and produced fewer tillers than WT plants. In addition, HPM panicles contained substantially more unfilled grains than WT panicles, indicating reduced seed setting (Fig. 1B). Quantitative measurements further supported these observations (Fig. 1C–E). The average plant height of HPM was 90.93 ± 4.65 cm, significantly lower than that of WT (97.91 ± 4.57 cm; *P* = 1.90 × 10⁻^1^⁷) (Fig. 1C). The average tiller number decreased from 29.79 ± 9.10 in WT to 20.27 ± 5.30 in HPM (*P* = 2.12 × 10⁻^13^) (Fig. 1D). The most pronounced difference was observed in seed-setting rate. HPM exhibited a substantially lower mean seed-setting rate (61.63 ± 40.77%; median 93.80%, IQR 18.22–97.67%) than WT (97.05 ± 2.93%; median 97.53%, IQR 95.96–99.00%; Welch’s t-test *P* = 7.96 × 10⁻^12^; Mann–Whitney U test *P* = 3.56 × 10⁻⁸) (Fig. 1E). Notably, the HPM distribution was bimodal: 37 of 84 plants (44%) showed severely reduced seed-setting (< 50%), whereas the remaining plants were largely comparable to WT. These results indicate that the HPM lineage differs markedly from WT in plant height, tiller number, and seed-setting rate after nine generations of self-pollination (Supplementary Table S8).

### Transposon-associated genomic variation and candidate TE insertions in HPM

**Fig. 2:**
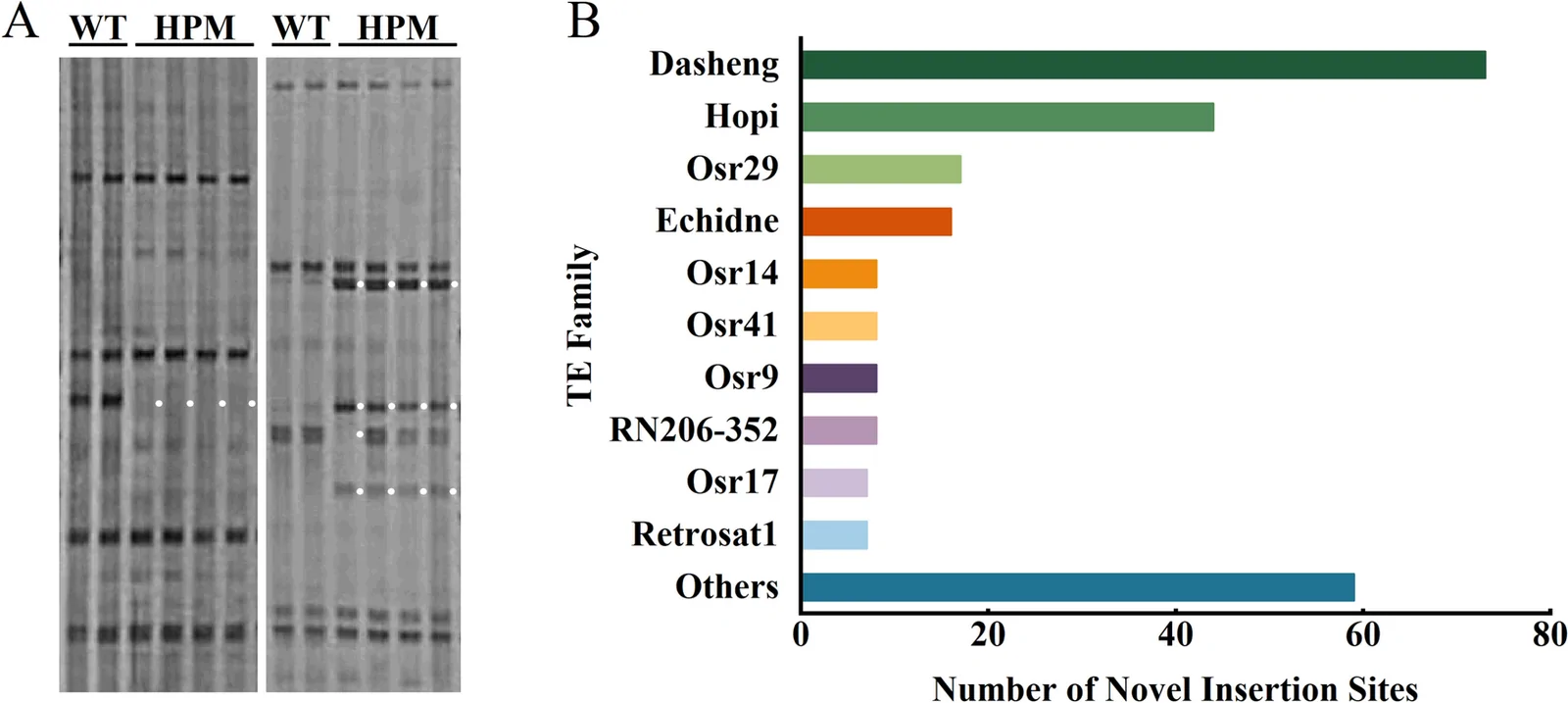
Transposon-associated genomic variation and candidate transposable element insertions in HPM. **A** Representative transposon display (TD) profiles of WT and four independent HPM plants. Arrows indicate polymorphic bands corresponding to putative transposon insertion or excision events. Approximately 1100 *mPing*-derived fragments generated from 29 primer combinations were scored. **B** Distribution of candidate HPM-specific transposable element (TE) insertion sites identified by whole-genome resequencing. A total of 255 candidate insertion sites were detected in HPM relative to WT. Dasheng, Hopi, Osr29, and Echidne accounted for the largest numbers of detected insertion sites. *WT* wild type, *HPM* hydrostatic pressure-derived lineage

To examine transposon-associated genomic variation in the hydrostatic pressure-derived lineage, transposon display (TD) analysis was performed using 29 primer combinations. Approximately 1100 *mPing*-derived amplified fragments were scored across four independent HPM plants. Compared with WT, both novel and missing bands were detected in all HPM plants, indicating the occurrence of transposon-associated genomic alterations (Fig. 2A). The frequencies of polymorphic TD bands ranged from 0.82% to 0.91% among the four HPM plants (Fig. 2A; Supplementary Table S2), indicating the presence of TE-associated polymorphisms in the ninth-generation lineage.

To further characterize TE mobilization at the genome-wide level, whole-genome resequencing data were analyzed for candidate HPM-specific TE insertion events (Supplementary Table S9). A total of approximately 255 candidate HPM-specific insertion sites were identified relative to WT. These insertion events were unevenly distributed among TE families, with Dasheng (73 insertions), Hopi (44 insertions), Osr29 (17 insertions), and Echidne (16 insertions) representing the most active families (Fig. 2B). Several additional TE families, including Osr14, Osr41, Osr9, RN206-352, Osr17, and Retrosat1, also showed multiple candidate insertions.

Notably, most of the newly inserted elements belonged to LTR retrotransposon families, particularly Dasheng and Hopi. Together, these findings reveal extensive transposon-associated genomic variation in the HPM lineage and indicate that transposon mobilization remained detectable after nine generations of self-pollination.

### DNA methylation alterations and their association with gene expression changes in HPM

**Fig. 3:**
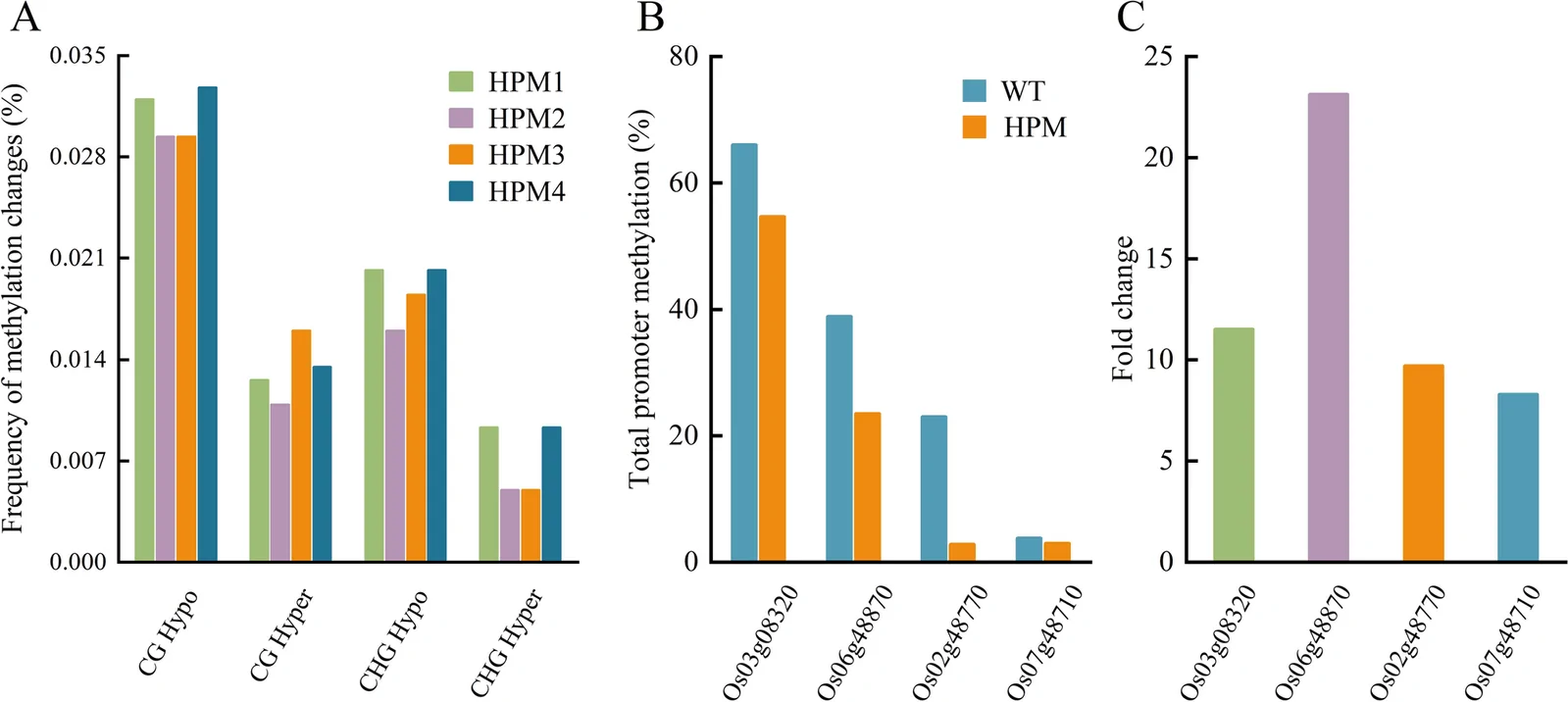
DNA methylation alterations and transcript abundance changes in HPM. **A** Frequencies of hypermethylation and hypomethylation events detected by MSAP analysis at CG and CHG sites in four independent HPM plants. MSAP analysis was performed using 20 selective primer combinations, generating 1189 reproducible fragments. **B** Total promoter methylation levels of four representative up-regulated genes determined by bisulfite sequencing. **C** Fold changes in transcript abundance of the corresponding genes identified by microarray analysis. *WT* wild type, *HPM* hydrostatic pressure-derived lineage

To investigate whether epigenetic modifications were associated with the phenotypic variation observed in HPM, DNA methylation patterns were analyzed using methylation-sensitive amplified polymorphism (MSAP). A total of 20 selective primer combinations generated 1,189 reproducible fragments across four independent HPM plants. Both hypermethylation and hypomethylation events were detected at CG and CHG sites (Fig. 3A). The frequencies of hypomethylation ranged from 2.94% to 3.28% at CG sites and from 1.60% to 2.02% at CHG sites, whereas hypermethylation frequencies ranged from 1.09% to 1.60% and from 0.50% to 0.93% at CG and CHG sites, respectively. In total, 4.63–5.72% of scored fragments exhibited methylation polymorphisms in each HPM plant (Fig. 3A, Supplementary Table S3). Overall, hypomethylation events were more frequent than hypermethylation events across all four HPM plants.

To further examine the relationship between DNA methylation and gene expression, four genes showing increased transcript abundance in the microarray analysis were selected for bisulfite sequencing of their promoter regions. These genes included *LOC_Os03g08320*, *LOC_Os06g48870*, *LOC_Os02g48770* and *LOC_Os07g48710*, whose transcript levels increased by 11.51-, 23.12-, 9.71- and 8.28-fold, respectively (Supplementary Table S10).

Bisulfite sequencing revealed reduced promoter methylation levels in HPM for three of the four genes, with reductions reaching statistical significance at individual cytosine positions for *LOC_Os03g08320* (5 of 31 positions), *LOC_Os06g48870* (8 of 80), and *LOC_Os02g48770* (2 of 62) by Fisher’s exact test (*P* < 0.05); the small change at *LOC_Os07g48710* (3.73% to 2.91%) was not statistically supported (0 of 71 positions) (Fig. 3B). The largest reduction was detected in *LOC_Os02g48770*, whose promoter methylation level decreased from 23.01% in WT to 2.81% in HPM. Substantial decreases were also observed for *LOC_Os06g48870* (38.85% to 23.48%) and *LOC_Os03g08320* (65.98% to 54.64%). In contrast, *LOC_Os07g48710* exhibited only a modest reduction in promoter methylation (3.73% to 2.91%). Detailed methylation profiles showed that methylation losses mainly occurred at CHG and CHH sites in several loci (Supplementary Figure S2). In parallel with these methylation changes, all four genes showed elevated transcript abundance in HPM (Fig. 3C). The concurrent occurrence of promoter methylation changes and increased transcript abundance was observed for all four examined genes (Fig. 3B, C). These locus-specific results are consistent with the general tendency toward reduced methylation in the HPM lineage revealed by MSAP, and support an association between methylation loss and transcriptional activation at the three loci with statistically supported changes.

### Transcriptional reprogramming in the HPM lineage

**Fig. 4:**
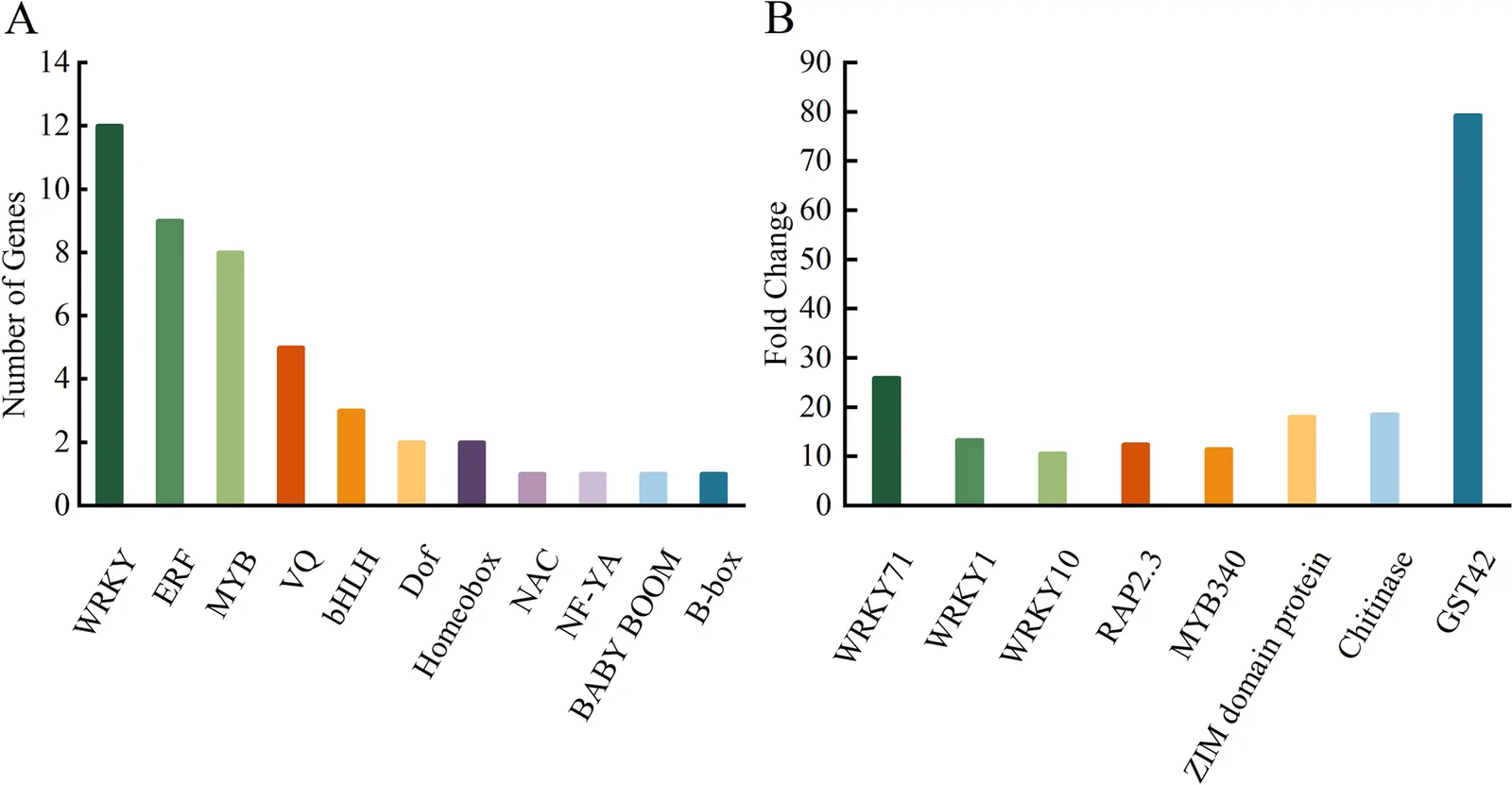
Differentially expressed transcription factor families and representative stress-responsive genes in HPM. **A** Distribution of transcription factor families represented among candidate differentially expressed genes identified by microarray analysis. Major families included WRKY, ERF/AP2, MYB, VQ, bHLH, Dof, NAC, and homeobox proteins. **B** Representative stress-responsive genes showing increased transcript abundance in HPM relative to WT. Expression fold changes were derived from Affymetrix microarray data. *WT* wild type, *HPM* hydrostatic pressure-derived lineage

To investigate whether the phenotypic and epigenetic alterations observed in HPM were accompanied by changes in gene expression, transcript abundance was compared between WT and HPM using the Affymetrix Rice Genome Array. A total of 1079 candidate differentially expressed probes (fold change ≥ 2, unadjusted *P* < 0.05) were identified between WT and HPM, of which 305 remained significant after Benjamini–Hochberg correction (FDR < 0.05) (Supplementary Table S10). Because functional annotation was performed on the full candidate list, the descriptions below are indicative rather than definitive. Functional annotation indicated that a substantial proportion of these probes corresponded to genes encoding transcriptional regulators and stress-responsive proteins. Among the transcription factor families represented, WRKY, MYB, ERF/AP2, VQ, bHLH, Dof, NAC, and homeobox proteins were among the most abundant (Fig. 4A). In total, 12 WRKY, 9 ERF/AP2, 8 MYB, and 5 VQ-associated genes exhibited altered expression in HPM.

Several stress-related genes showed marked increases in transcript abundance in HPM relative to WT (Fig. 4B). These included WRKY transcription factors (*WRKY71*, *WRKY1*, and *WRKY10*), AP2/ERF family members (*RAP2.3*), MYB-related transcription factors, ZIM domain-containing proteins, chitinases, and glutathione S-transferases. Among them, *WRKY71* showed a 25.98-fold increase in expression, whereas *GST42* exhibited a 79.34-fold increase. Genes associated with defense responses, reactive oxygen species detoxification, and cell wall-related processes were also represented among the up-regulated transcripts. Overall, the microarray analysis revealed extensive changes in transcript abundance between WT and HPM, involving multiple transcription factor families and stress-associated genes. The marked representation of stress-responsive transcription factor families and defense-related genes suggests that the transcriptional reprogramming of the HPM lineage involves stress-associated regulatory pathways.

To validate the microarray-based expression changes, we examined the transcript levels of 12 selected genes (including both up- and down-regulated genes) by quantitative RT-PCR. The qRT-PCR results were consistent with the direction of the expression changes detected by the microarray (Supplementary Figure S3).

## Discussion

### Hydrostatic pressure-associated long-term phenotypic variation

In the present study, a hydrostatic pressure-derived rice lineage (HPM) exhibited significant reductions in plant height, tiller number, and seed-setting rate compared with the wild-type cultivar JL307. Importantly, these phenotypic differences were still evident in the ninth selfed generation, suggesting that the pressure-associated variation persisted through at least nine generations of selfing.

Environmental stresses are increasingly recognized as potential sources of heritable phenotypic variation (Lämke and Bäurle 2017; Herman and Sultan 2011). Previous studies have shown that abiotic stresses, including drought, salinity, temperature extremes, and tissue culture conditions, can induce genetic and epigenetic changes that may persist beyond the initial stress exposure (Lang-Mladek et al. 2010; Molinier et al. 2006; Wibowo et al. 2016). Such stress-associated molecular alterations have been proposed to contribute to phenotypic diversification and environmental adaptation (Herman and Sultan 2011; Lisch 2013). However, compared with other abiotic stresses, relatively little is known about the long-term biological consequences of hydrostatic pressure in higher plants (Liu et al. 2008; Yan et al. 2007).

The continued expression of the HPM phenotype in the ninth generation suggests that hydrostatic pressure treatment was associated with molecular changes that remained detectable long after the initial stress event. The pronounced reduction in seed-setting rate further suggests that reproductive performance was affected in the HPM lineage. Although the molecular basis of these phenotypic alterations remains unresolved, the long-term maintenance of the phenotype is consistent with the possibility that genomic and/or epigenomic changes are associated with its persistence (Lämke and Bäurle 2017; Wibowo et al. 2016).

Taken together, the present results indicate that hydrostatic pressure treatment can be associated with long-lasting phenotypic variation in rice. The HPM lineage, therefore, provides a useful system for investigating the molecular processes potentially involved in stress-associated heritable variation. The phenotypic changes observed in the HPM lineage are consistent with earlier reports that hydrostatic pressure treatment of rice seeds can induce heritable alterations in agronomic traits, including plant height, panicle number, grain size, and fertility (Bai et al. 2003b). Because hydrostatic pressure can both generate heritable phenotypic variation and trigger the mobilization of the transposable element *mPing* (Lin et al. 2006), it may complement conventional mutagens in rice breeding, for example as a physical mutagen for generating novel germplasm or as a tool for transposon-based gene tagging.

### Transposon-associated genomic variation in the HPM lineage

Transposable elements (TEs) are major components of plant genomes and represent an important source of genetic variation (Lisch 2013). Under normal conditions, most TEs remain transcriptionally silent through epigenetic mechanisms, such as DNA methylation and chromatin modification (Law and Jacobsen 2010; Slotkin and Martienssen 2007). However, accumulating evidence indicates that environmental stresses can disrupt TE silencing and promote transposon mobilization, leading to genomic variation and, in some cases, altered gene regulation (Ito 2022; Ito et al. 2011; McClintock 1984).

In rice and other plant species, several transposon families have been reported to respond to environmental stimuli, including tissue culture, pathogen challenge, temperature stress, and other abiotic stresses (Ito 2022; McClintock 1984; Negi et al. 2016). Stress-associated TE mobilization can generate novel insertion sites, alter local chromatin environments, and potentially influence the expression of nearby genes (Lisch 2013; Ito 2022). Among these stress-responsive elements, several rice transposon families have been shown to exhibit increased mobility under environmental or genomic perturbations, suggesting that at least some rice transposon families can become mobilized following environmental or genomic perturbations (Naito et al. 2006; Nakazaki et al. 2003).

In the present study, transposon display detected polymorphic bands in all four independent HPM plants, with a TD band polymorphism rate of 0.82–0.91%, and whole-genome resequencing identified 255 candidate HPM-specific insertion sites, most notably in the LTR retrotransposon families Dasheng (73 insertions) and Hopi (44 insertions). The distribution of insertions across multiple TE families indicates that the genomic changes associated with hydrostatic pressure treatment were not restricted to a single transposon lineage. The two assays differ in scope by design: transposon display targeted *mPing*, the MITE previously associated with pressure treatment in this system and monitored by locus-specific PCR during line maintenance, whereas the resequencing survey used a TE reference collection that did not include *mPing* and, therefore, reported candidate insertions from other families, predominantly the LTR retrotransposons Dasheng and Hopi.

The predominance of retrotransposon-related insertions is consistent with the biology of LTR retrotransposons, which constitute the most abundant TE class in the rice genome (International Rice Genome Sequencing Project 2005) and whose activity can be enhanced under stress conditions (Ito et al. 2011; Ito 2022). Because retrotransposons propagate through a copy-and-paste mechanism that does not require excision, even a limited number of activation events can generate multiple new insertions and thereby increase genomic diversity (Lisch 2013). The preferential recovery of insertions in the LTR families Dasheng and Hopi in HPM is, therefore, consistent with the amplification of these families through retrotransposition bursts.

The TE mobilization observed in the HPM lineage is comparable to that reported in other stress systems. For example, tissue culture induces a transpositional burst of diverse transposable elements in rice callus, accompanied by complex genome-wide changes in DNA methylation (Hu et al. 2019), and various abiotic stresses have been reported to activate transposable elements in plants (Ito et al. 2011; Ito 2022; Negi et al. 2016).

Although the present data did not establish a direct causal relationship between individual TE insertions and the observed phenotypic alterations, the combined evidence from transposon display and resequencing analyses indicates that hydrostatic pressure treatment was associated with substantial transposon mobilization. Such TE-associated genomic variation may be associated with the establishment or maintenance of the phenotypic differences observed in HPM. More broadly, these findings support the view that transposable elements can serve as important generators of genetic variation during plant responses to environmental stress (Lisch 2013; Ito 2022; McClintock 1984).

### Epigenetic alterations are associated with transcriptional reprogramming in HPM.

In the present study, MSAP analysis revealed methylation alterations at the sampled CCGG sites in HPM plants. Across all four independent HPM plants, the frequency of hypomethylation events exceeded that of hypermethylation events in both CG and CHG contexts, indicating a general tendency toward reduced methylation in the HPM lineage. This observation is consistent with previous reports showing that environmental stresses are frequently accompanied by changes in DNA methylation patterns and broader epigenetic alterations in plants (Lämke and Bäurle 2017; Wibowo et al. 2016). It should be noted that MSAP assesses methylation variation at CCGG restriction sites and, therefore, does not provide genome-wide methylation levels; the conclusions drawn from the MSAP data are limited to the sampled loci.

Similarly, stress-associated changes in DNA methylation have been documented for drought, salinity, and temperature treatments (Ma et al. 2025; Zhang et al. 2018; Lodhi and Srivastava 2025), consistent with the methylation alterations detected in the HPM lineage. These parallels suggest that hydrostatic pressure may act through stress-associated mechanisms shared with other stimuli.

To further examine the relationship between DNA methylation and gene expression, four genes displaying strong transcriptional upregulation were selected for locus-specific bisulfite sequencing analysis. Three of the four genes exhibited substantial reductions in promoter methylation levels in HPM compared with WT. Notably, the small methylation change at *LOC_Os07g48710* (3.73% to 2.91%) was not significant at any individual cytosine position and is, therefore, considered to fall within clone sampling variation; only the three loci with statistically supported reductions are discussed as strong associations. Particularly notable decreases were detected in *LOC_Os02g48770* and *LOC_Os06g48870*, where total methylation levels were markedly reduced. These methylation changes were accompanied by significant increases in transcript abundance, suggesting a negative association between promoter methylation and gene expression (Law and Jacobsen 2010; Zhang et al. 2018). However, only four loci were examined, and, therefore, the extent to which this relationship applies more broadly across the genome remains unclear.

Microarray-based gene expression profiling further revealed extensive changes in transcript abundance in HPM. Numerous genes associated with stress responses, signal transduction, transcriptional regulation, transport processes, and defense-related functions were differentially expressed. Several transcription factor families, including WRKY, MYB, ERF/AP2, NAC, bHLH, and VQ-associated regulators, were prominently represented among the upregulated genes. These transcription factors are widely implicated in plant stress signaling networks and indicate substantial alterations in transcriptional regulation within the HPM lineage (Lämke and Bäurle 2017).

The coexistence of altered DNA methylation patterns and widespread transcriptional changes suggests a potential relationship between epigenetic variation and gene expression changes in HPM (Law and Jacobsen 2010; Zhang et al. 2018). Although the present study does not establish a direct causal relationship between methylation changes and the expression of all differentially expressed genes, the promoter-specific bisulfite sequencing results provide evidence that methylation loss can be associated with transcriptional activation at individual loci. However, only four loci were examined, and broader genome-wide relationships between DNA methylation and gene expression remain to be investigated. Together, these findings suggest that epigenetic alterations may be associated with altered gene expression patterns in the HPM lineage (Lämke and Bäurle 2017; Ma et al. 2025).

### An integrated framework for hydrostatic pressure-associated persistent variation in rice

The present study combined phenotypic analysis, transposon display, whole-genome resequencing, DNA methylation analysis, and microarray-based expression profiling to characterize a rice HPM lineage derived from hydrostatic pressure-treated seeds. Collectively, these data indicate that the HPM lineage displaying long-term phenotypic differences was also characterized by multiple layers of molecular variation, including transposon-associated genomic variation, altered DNA methylation patterns, and widespread changes in gene expression.

Hydrostatic pressure is an unusual environmental stimulus that has been reported to affect diverse cellular processes in plants (Liu et al. 2008; Yan et al. 2007). In the HPM lineage, multiple layers of molecular variation—transposon-associated genomic changes, altered DNA methylation patterns, and extensive transcriptional reprogramming—co-occur and remained detectable after nine generations of self-pollination. Because transcription factors are central regulators of developmental and environmental response pathways, their altered expression may reflect broad regulatory adjustments associated with the HPM phenotype (Lämke and Bäurle 2017), and the concurrent promoter methylation changes and increased transcript abundance at several loci are consistent with an association between epigenetic variation and transcriptional regulation (Law and Jacobsen 2010; Zhang et al. 2018).

Although transposon-associated variation, DNA methylation changes, and altered gene expression were detected simultaneously in HPM, the causal relationships among these molecular processes cannot be determined from the present data. It, therefore, remains unclear whether these changes represent independent consequences of hydrostatic pressure treatment or whether interactions among them were associated with the observed phenotype. Nevertheless, their co-occurrence is consistent with previous studies suggesting that environmental stress can be associated with interconnected genomic and epigenomic processes, including transposable element activity, epigenetic remodeling, and transcriptional reprogramming (Zhang et al. 2018; Lisch 2013; Ito 2022; McClintock 1984).

**Fig. 5:**
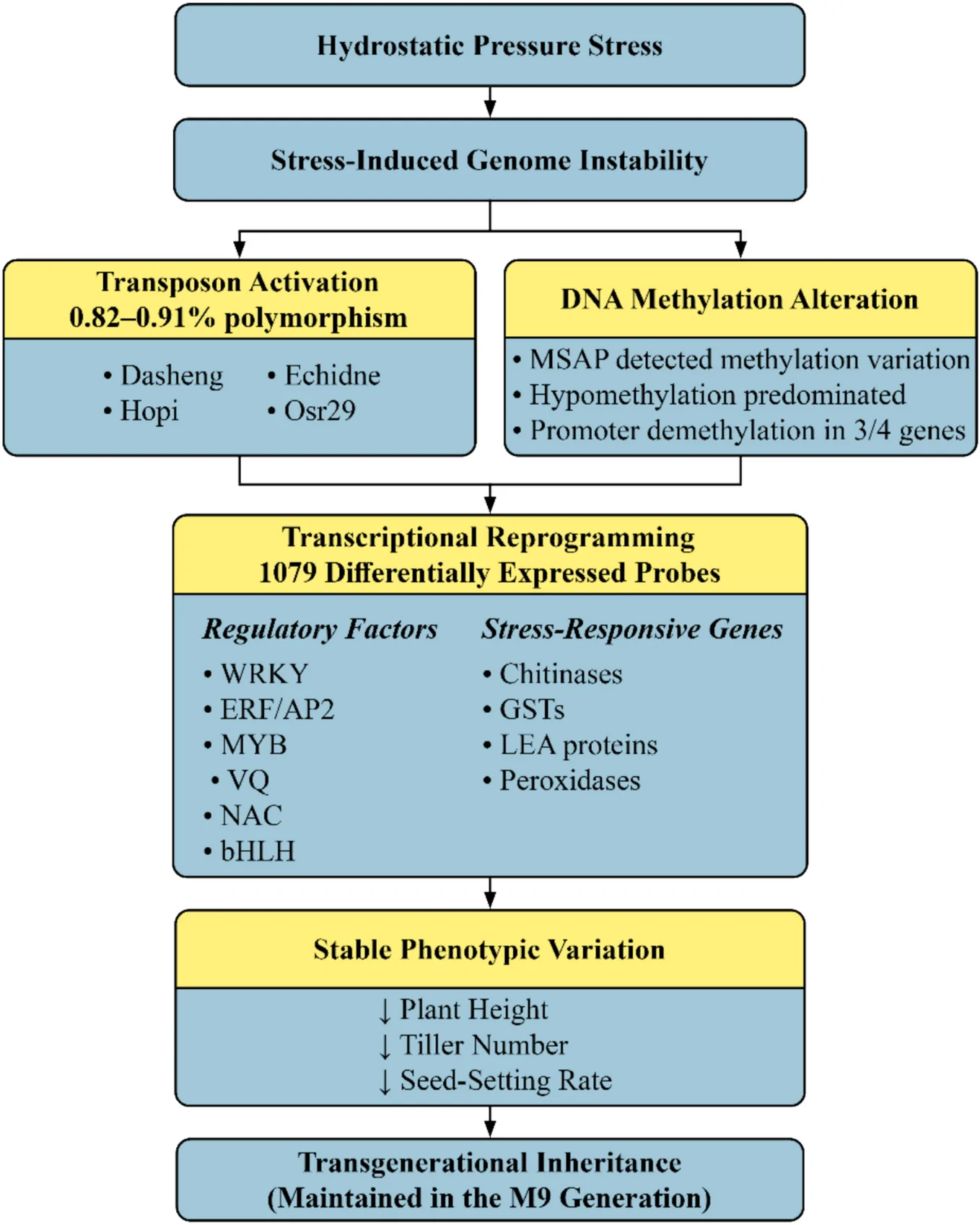
Conceptual framework summarizing molecular changes associated with hydrostatic pressure-derived variation in rice

Taken together, our findings indicate that hydrostatic pressure treatment can be associated with persistent, multi-level molecular variation—encompassing transposon mobilization, DNA methylation changes, and transcriptional reprogramming—in a rice lineage displaying long-term phenotypic differences (Fig. 5). To our knowledge, this is the first study to demonstrate such associations in a selfed lineage derived from hydrostatic pressure-treated seeds. These observations are broadly consistent with current models of stress-associated molecular memory and heritable variation in plants (Lämke and Bäurle 2017; Wibowo et al. 2016). Further studies employing genome-wide methylome analyses, detailed characterization of transposon insertion events, and functional validation of candidate genes will be required to clarify how hydrostatic pressure-associated molecular variation contributes to long-term phenotypic differences in rice.

Several limitations of this study should be acknowledged. First, because the analyzed material derives from a single lineage maintained by repeated self-pollination, the theoretical contributions of spontaneous mutation or cumulative selection during propagation cannot be formally excluded. Second, the present study is descriptive: although transposon-associated variation, DNA methylation changes, and transcriptional reprogramming co-occur in the HPM lineage, direct causal relationships between hydrostatic pressure treatment and these alterations, or between the individual molecular changes and the observed phenotype, cannot be established from our data. Third, certain equipment-specific parameters of the original pressure treatment (e.g., pressure chamber model, rates of pressure increase and release) were not recorded in the source study. Fourth, the relationship between promoter methylation and gene expression was examined at only four loci, and genome-wide associations remain to be investigated. Fifth, the phenotypic evaluation was conducted in a single growing season, and multi-year field trials would further strengthen the conclusions. Sixth, the WT sequencing depth was moderate (–13.2 ×), and low-frequency variants in the parental genotype may have been incompletely captured. Seventh, individual TE insertion events were not validated by band-excision sequencing or locus-specific PCR, and the zygosity of the insertions was not determined; the evidence for TE activation relies on the concordance between transposon display and whole-genome resequencing. Eighth, although the phenotypic differences persisted across nine generations of self-pollination, this persistence alone does not establish Mendelian inheritance; segregation analysis through reciprocal crosses and characterization of multiple independent lineages would be required to demonstrate heritability, and such analyses were not performed. Ninth, the differential expression analysis used an unadjusted P value threshold together with a fold-change cutoff, and the 1,079 probes reported here should, therefore, be regarded as candidates; 305 of them remained significant after Benjamini–Hochberg correction (FDR < 0.05), and individual genes would require independent validation. In addition, genetic variants other than TE insertions (e.g., single-nucleotide variants, small insertions/deletions, copy-number changes, and residual heterozygosity) were not analyzed, and their potential contribution to the observed phenotype cannot be excluded. These points warrant further investigation in future studies.

The hydrostatic pressure-derived HPM lineage was characterized by transposon-associated genomic variation, alterations in DNA methylation patterns, and changes in gene expression, including differential expression of multiple stress-responsive transcription factors and downstream genes. The co-occurrence of these molecular changes was associated with the long-term phenotypic differences observed in the HPM lineage. The figure summarizes associations identified in the present study and does not imply direct causal relationships among the individual molecular processes.

## Conclusion

In this study, we show that a rice lineage (HPM) derived from hydrostatic pressure-treated seeds displayed persistent reductions in plant height, tiller number, and seed-setting rate over nine generations of self-pollination, accompanied by transposon mobilization, DNA methylation alterations, and extensive transcriptional reprogramming. These findings demonstrate that hydrostatic pressure—an unusual physical stimulus—can be associated with persistent, multi-level molecular variation in rice, and they provide a useful experimental system for dissecting the molecular basis of stress-associated heritable variation. In addition, the pressure-derived lineage may offer material for exploring the use of hydrostatic pressure as a tool in plant breeding.

## Supplementary Information

Below is the link to the electronic supplementary material.

Supplementary file1 (DOCX 1240 KB)

Supplementary file2 (XLSX 50 KB)

Supplementary file3 (DOCX 564 KB)

Supplementary file4 (XLSX 10 KB)

Supplementary file5 (XLSX 18 KB)

Supplementary file6 (XLSX 73 KB)

## Data Availability

No datasets were generated or analysed during the current study.
