# Supplementary material for: Hydrostatic pressure-induced heritable variation in rice is associated with transposon mobilization, DNA methylation changes, and transcriptional reprogramming
Source: Plant Cell Rep. 2026 Oct 6;45(11):322. doi: 10.1007/s00299-026-04009-y (PMC13642388; doi:10.1007/s00299-026-04009-y)
Supplement: Supplementary file 1 [file 299_2026_4009_MOESM1_ESM.docx]

Supplementary Information

**A**

·

**B**

Figure S1. Principal component analysis (A) and sample correlation (B) of the six microarrays.

Figure S2. Cytosine methylation patterns in promoter regions of four representative genes analyzed by bisulfite sequencing.

DNA methylation profiles of LOC_Os03g08320, LOC_Os06g48870, LOC_Os02g48770, and LOC_Os07g48710 were visualized using CyMATE. Methylation status at CG, CHG, and CHH sites is shown for WT and HPM plants. Filled circles indicate methylated cytosines, whereas open circles indicate unmethylated cytosines.

Figure S3. Validation of microarray expression changes by quantitative RT-PCR. Relative expression levels of 12 selected genes (including up- and down-regulated genes) in WT and HPM are shown (GAPDH as reference; mean ± SD of three biological replicates).

| **Table S1. Primers used for transposon display (TD) analysis.** | | |
| --- | --- | --- |
| Primer | Step | Sequence (5' to 3') |
| MseI pre-selective primer | Pre-amplification | GATGAGTCCTGAGTAA |
| mPing primer I | Pre-amplification | GCTGACGAGTTTCACCAGGATG |
| mPing primer II | Selective amplification | TGTGCATGACACACCAGTG |
| M1 | Selective amplification | GATGAGTCCTGAGTAATCA |
| M2 | Selective amplification | GATGAGTCCTGAGTAATCC |
| M3 | Selective amplification | GATGAGTCCTGAGTAATGC |
| M4 | Selective amplification | GATGAGTCCTGAGTAATCG |
| M5 | Selective amplification | GATGAGTCCTGAGTAATGG |
| M6 | Selective amplification | GATGAGTCCTGAGTAACAT |
| M7 | Selective amplification | GATGAGTCCTGAGTAACAC |
| M8 | Selective amplification | GATGAGTCCTGAGTAACAG |
| M9 | Selective amplification | GATGAGTCCTGAGTAACTA |
| M10 | Selective amplification | GATGAGTCCTGAGTAACTC |
| M11 | Selective amplification | GATGAGTCCTGAGTAACTG |
| M12 | Selective amplification | GATGAGTCCTGAGTAACCA |
| M13 | Selective amplification | GATGAGTCCTGAGTAACCT |
| M14 | Selective amplification | GATGAGTCCTGAGTAACCG |
| M15 | Selective amplification | GATGAGTCCTGAGTAACGA |
| M16 | Selective amplification | GATGAGTCCTGAGTAACGT |
| M17 | Selective amplification | GATGAGTCCTGAGTAACGC |
| M18 | Selective amplification | GATGAGTCCTGAGTAACGG |
| M19 | Selective amplification | GATGAGTCCTGAGTAAGAT |
| M20 | Selective amplification | GATGAGTCCTGAGTAAGAC |
| M21 | Selective amplification | GATGAGTCCTGAGTAAGAG |
| M22 | Selective amplification | GATGAGTCCTGAGTAAGTA |
| M23 | Selective amplification | GATGAGTCCTGAGTAAGTC |
| M24 | Selective amplification | GATGAGTCCTGAGTAAGTG |
| M25 | Selective amplification | GATGAGTCCTGAGTAAGCA |
| M26 | Selective amplification | GATGAGTCCTGAGTAAGCT |
| M27 | Selective amplification | GATGAGTCCTGAGTAAGCC |
| M28 | Selective amplification | GATGAGTCCTGAGTAAGCG |
| M29 | Selective amplification | GATGAGTCCTGAGTAAGGT |

| **Table S2 Transposon display (TD) results in four independent HPM plants** | | | |
| --- | --- | --- | --- |
| Line | Total bands scored | Polymorphic bands | Polymorphism rate (%) |
| HPM-1 | 1100 | 9 | 0.82 |
| HPM-2 | 1100 | 10 | 0.91 |
| HPM-3 | 1100 | 10 | 0.91 |
| HPM-4 | 1100 | 10 | 0.91 |

| **Table S3. Summary of MSAP methylation statistics in WT and four independent HPM plants. For each plant, the numbers of fragments in each of the four MSAP types and of CG- and CHG-related hypomethylation and hypermethylation events are listed, with percentages relative to the total number of scored fragments (1,189).** | | | | | | | | | | | | |
| --- | --- | --- | --- | --- | --- | --- | --- | --- | --- | --- | --- | --- |
| Sample | TypeI_11 | TypeII_01 | TypeIII_10 | TypeIV_00 | CG_hypo_n | CG_hypo_pct | CG_hyper_n | CG_hyper_pct | CHG_hypo_n | CHG_hypo_pct | CHG_hyper_n | CHG_hyper_pct |
| WT | 870 | 245 | 40 | 34 |  |  |  |  |  |  |  |  |
| HPM1 | 895 | 233 | 38 | 23 | 38 | 3.2 | 15 | 1.26 | 24 | 2.02 | 11 | 0.93 |
| HPM2 | 894 | 234 | 38 | 23 | 35 | 2.94 | 13 | 1.09 | 19 | 1.6 | 6 | 0.5 |
| HPM3 | 886 | 245 | 40 | 18 | 35 | 2.94 | 19 | 1.6 | 22 | 1.85 | 6 | 0.5 |
| HPM4 | 891 | 237 | 42 | 19 | 39 | 3.28 | 16 | 1.35 | 24 | 2.02 | 11 | 0.93 |

Table S5 Bisulfite sequencing primers

| **Name** | **Sequence** |
| --- | --- |
| LOC_Os03g08320_F | ATATAGYAATTATGYTTAAAAGATTTG |
| LOC_Os03g08320_R | ACATAARAACCAARAAACTCT |
| LOC_Os06g48870_F | AAGAGTTGGATAAAGATTAAGATTTT |
| LOC_Os06g48870_R | CTTATTATTTCATRACTTATATATCT |
| LOC_Os02g48770_F | ATGTGAYAGTGTAGAGTATATATTTTT |
| LOC_Os02g48770_R | CACRARTACTTRTCAATTTATA |
| LOC_Os07g48710_F | AGAAAAAAAAATAATATTAAGTGGATG |
| LOC_Os07g48710_R | CATATRCCATCCATCRCCTATT |

| File | Threshold Test | Spike_AFFX-r2-Bs-dap_3-5-ratio | Spike_AFFX-r2-Bs-lys_3-5-ratio | Spike_AFFX-r2-Bs-phe_3-5-ratio | Spike_AFFX-r2-Bs-thr_3-5-ratio | Spike_AFFX-r2-Ec-bioB_3-5-ratio | Spike_AFFX-r2-Ec-bioC_3-5-ratio | Spike_AFFX-r2-Ec-bioD_3-5-ratio | Spike_AFFX-r2-P1-cre_3-5-ratio | pm_mean | mm_mean | all_probeset_mean | all_probeset_rle_mean | all_probeset_rle_stdev |
| --- | --- | --- | --- | --- | --- | --- | --- | --- | --- | --- | --- | --- | --- | --- |
| HPM-1_(Rice).rma | Within Bounds | 2.108209 | 0.172826 | -0.259285 | 1.837446 | 0.168046 | 0.25944 | 0.636191 | 0.185236 | 654.44751 | 254.528046 | 6.563014 | 0.154843 | 0.251799 |
| HPM-2_(Rice).rma | Within Bounds | 1.897911 | 0.396117 | -0.291204 | 1.638193 | 0.320724 | 0.301113 | 0.526495 | 0.136641 | 594.702393 | 246.347687 | 6.55929 | 0.159842 | 0.170239 |
| HPM-3_(Rice).rma | Within Bounds | 1.838705 | 0.224549 | -0.114529 | 1.704159 | 0.187031 | 0.368973 | 0.527709 | 0.241088 | 648.468628 | 254.751083 | 6.565989 | 0.167659 | 0.242297 |
| WT-1_(Rice).rma | Within Bounds | 2.409081 | 0.14085 | -0.276781 | 1.858772 | 0.031585 | 0.268584 | 0.567438 | 0.156164 | 858.250671 | 336.15863 | 6.545742 | 0.127616 | 0.147878 |
| WT-2_(Rice).rma | Within Bounds | 1.874911 | 0.050988 | -0.385038 | 1.549229 | 0.131301 | 0.271976 | 0.446871 | 0.132877 | 644.890686 | 243.578979 | 6.55159 | 0.125526 | 0.14194 |
| WT-3_(Rice).rma | Within Bounds | 1.776796 | 0.054659 | -0.551828 | 1.416901 | 0.162235 | 0.312019 | 0.51803 | 0.187995 | 721.997681 | 261.323059 | 6.554667 | 0.146449 | 0.16269 |

Table S6. Quality-control metrics for the six Affymetrix Rice Genome Arrays used in this study.

| Table S7. Primers used for quantitative RT-PCR validation of 12 selected genes | | |
| --- | --- | --- |
| Probe ID | Forward primer (5'-3') | Reverse primer (5'-3') |
| Os.40021.1.S1_a_at | 5' CACCAGCCACAAGCTACTCCA 3' | 5' ACCCTGACCCGCTCCACTT 3' |
| Os.52449.1.S1_at | 5' GCTCTTGCCATCCTTGTCC 3' | 5' TAGCCCGATCCACCGTAA 3' |
| Os.57401.1.S1_at | 5' TCGTACAAAGCGGGACTT 3' | 5' CCTGTATGAACCCTGAGATG 3' |
| Os.7610.1.S1_a_at | 5' AGGCATCAAGACAGAACAGC 3' | 5' GATACACGAAGGCAGCAGTA 3' |
| Os.13781.1.S2_at | 5' CACGCCAAAGCAAGAAGC 3' | 5' ACATACGCCCACAGTAAGAT 3' |
| Os.49042.1.A1_s_at | 5' GGGAAGAGGAGGACGATG 3' | 5' TCAAGCAACTCCCGAACA 3' |
| Os.25355.1.A1_at | 5' TCTCAGACTAAAGCGTCCAT 3' | 5' CTCATACCAAGTCCTCCAAA 3' |
| Os.17921.1.S1_at | 5' TCCTGAATAATCATGGGTTT 3' | 5' GAACAACTGGATGGCTGAA 3' |
| Os.15247.1.S1_s_at | 5' TCAGGAACGACGAGGAGC 3' | 5' AAACAAGCAAAGCGAGGC 3' |
| Os.6723.1.S1_s_at | 5' TCCAATCCACGGAGTCAT 3' | 5' ACCAGGCGAATCAAAGAG 3' |
| Os.18955.1.S1_at | 5' ATTGCCTTGATTCTTGGG 3' | 5' CCTCGTCGTATGAGACAGC 3' |
| Os.10356.1.S1_at | 5' GAAGGAGCACAGTGGAAGCA 3' | 5' CCCGCCGTAGAAGATGGT 3' |

| Table S8. Raw phenotypic data for WT and HPM plants. | | | | | | |
| --- | --- | --- | --- | --- | --- | --- |
| Plant | WT_height_cm | HPM_height_cm | WT_tillers | HPM_tillers | WT_seed_setting_% | HPM_seed_setting_% |
| 1 | 90 | 74 | 15 | 11 | 100 | 100 |
| 2 | 90 | 78 | 15 | 11 | 99.13 | 100 |
| 3 | 90 | 79 | 15 | 11 | 100 | 98.81 |
| 4 | 91 | 81 | 16 | 12 | 97.06 | 97.3 |
| 5 | 91 | 82 | 16 | 12 | 98.33 | 99.26 |
| 6 | 91 | 85 | 16 | 12 | 97.89 | 100 |
| 7 | 92 | 85 | 17 | 13 | 93.81 | 96.61 |
| 8 | 92 | 86 | 17 | 13 | 100 | 99.03 |
| 9 | 92 | 86 | 18 | 13 | 100 | 100 |
| 10 | 92 | 86 | 18 | 13 | 82.19 | 99.15 |
| 11 | 92 | 86 | 18 | 14 | 98.96 | 100 |
| 12 | 92 | 86 | 18 | 14 | 87.21 | 91.67 |
| 13 | 92 | 86 | 18 | 14 | 98.51 | 95.7 |
| 14 | 93 | 87 | 18 | 14 | 100 | 93.16 |
| 15 | 93 | 87 | 19 | 14 | 99.05 | 98.91 |
| 16 | 93 | 87 | 20 | 15 | 98.8 | 16.28 |
| 17 | 93 | 87 | 20 | 15 | 99.34 | 1.94 |
| 18 | 94 | 88 | 21 | 15 | 93.06 | 23.08 |
| 19 | 94 | 88 | 22 | 15 | 98.75 | 22.45 |
| 20 | 94 | 89 | 22 | 16 | 98.73 | 19.05 |
| 21 | 94 | 89 | 22 | 16 | 94.44 | 4.76 |
| 22 | 94 | 89 | 22 | 16 | 98.61 | 27.85 |
| 23 | 95 | 89 | 23 | 16 | 97.3 | 19.05 |
| 24 | 95 | 89 | 24 | 16 | 96.8 | 12.12 |
| 25 | 95 | 89 | 24 | 16 | 99.1 | 14.29 |
| 26 | 95 | 90 | 24 | 16 | 98.8 | 20 |
| 27 | 95 | 90 | 24 | 17 | 97.3 | 13.27 |
| 28 | 95 | 90 | 25 | 18 | 96.63 | 19.79 |
| 29 | 96 | 90 | 25 | 18 | 97.53 | 2.06 |
| 30 | 96 | 90 | 26 | 18 | 100 | 8.06 |
| 31 | 96 | 90 | 26 | 18 | 97.47 | 9.46 |
| 32 | 96 | 90 | 27 | 19 | 95.24 | 14.89 |
| 33 | 97 | 90 | 27 | 19 | 94.44 | 97.32 |
| 34 | 97 | 90 | 27 | 19 | 100 | 96.55 |
| 35 | 97 | 91 | 28 | 19 | 96.94 | 96 |
| 36 | 97 | 91 | 28 | 19 | 98.88 | 95.52 |
| 37 | 98 | 91 | 28 | 19 | 91.78 | 97.37 |
| 38 | 98 | 91 | 29 | 20 | 94.44 | 96.49 |
| 39 | 98 | 91 | 30 | 20 | 100 | 21.05 |
| 40 | 98 | 91 | 30 | 20 | 100 | 91.49 |
| 41 | 98 | 91 | 30 | 20 | 98.31 | 97.76 |
| 42 | 98 | 92 | 30 | 20 | 99.03 | 96.91 |
| 43 | 99 | 92 | 30 | 21 | 98.92 | 97.22 |
| 44 | 99 | 92 | 31 | 21 | 95.96 | 96.55 |
| 45 | 99 | 93 | 31 | 21 | 97.03 | 96.46 |
| 46 | 99 | 93 | 32 | 22 | 94.59 | 95.54 |
| 47 | 99 | 93 | 32 | 22 | 97.64 | 93.75 |
| 48 | 100 | 93 | 32 | 22 | 97.08 | 98.55 |
| 49 | 100 | 93 | 32 | 22 | 91.3 | 100 |
| 50 | 100 | 93 | 32 | 22 | 99.2 | 96.33 |
| 51 | 100 | 93 | 33 | 22 | 99 | 97.73 |
| 52 | 100 | 94 | 33 | 22 | 96.26 | 95.83 |
| 53 | 100 | 94 | 33 | 23 | 97.73 | 1.74 |
| 54 | 100 | 94 | 34 | 23 | 98 | 33.33 |
| 55 | 100 | 94 | 34 | 23 | 95.08 | 26.67 |
| 56 | 101 | 94 | 34 | 23 | 97.17 | 15.29 |
| 57 | 101 | 94 | 35 | 23 | 86.59 | 30.77 |
| 58 | 101 | 94 | 35 | 24 | 98.77 | 10.77 |
| 59 | 101 | 94 | 36 | 24 | 95.65 | 13.58 |
| 60 | 101 | 94 | 36 | 24 | 96.83 | 31.86 |
| 61 | 101 | 94 | 37 | 25 | 97.73 | 25 |
| 62 | 101 | 95 | 37 | 25 | 98.86 | 17.95 |
| 63 | 102 | 95 | 37 | 25 | 96.91 | 37.68 |
| 64 | 102 | 95 | 38 | 25 | 100 | 22.45 |
| 65 | 102 | 95 | 39 | 25 | 94.64 | 96.88 |
| 66 | 102 | 95 | 39 | 26 | 97.37 | 13.73 |
| 67 | 102 | 95 | 39 | 26 | 100 | 2.35 |
| 68 | 103 | 95 | 40 | 26 | 91.67 | 0.88 |
| 69 | 103 | 96 | 41 | 26 | 95.38 | 32.89 |
| 70 | 103 | 96 | 41 | 27 | 96.94 | 8.18 |
| 71 | 104 | 96 | 41 | 27 | 94.59 | 6.77 |
| 72 | 104 | 96 | 41 | 27 | 94.12 | 12.2 |
| 73 | 104 | 96 | 42 | 27 | 100 | 97.87 |
| 74 | 104 | 96 | 42 | 27 | 96.55 | 94.85 |
| 75 | 105 | 96 | 42 | 27 | 96.45 | 96.19 |
| 76 | 105 | 97 | 42 | 28 | 96.95 | 98.25 |
| 77 | 106 | 97 | 42 | 28 | 97.4 | 93.85 |
| 78 | 107 | 97 | 43 | 28 | 98.25 | 100 |
| 79 | 107 | 97 | 45 | 28 | 99.02 | 98.44 |
| 80 | 107 | 97 | 46 | 29 | 98.04 | 98.2 |
| 81 |  |  | 47 | 29 | 100 | 97.5 |
| 82 |  |  | 49 | 31 | 100 | 97.93 |
| 83 |  |  |  |  | 98.72 | 99.13 |
| 84 |  |  |  |  | 98.86 | 91.36 |
| 85 |  |  |  |  | 99.04 |  |
| 86 |  |  |  |  | 98.99 |  |
| 87 |  |  |  |  | 94.12 |  |
| 88 |  |  |  |  | 96 |  |
| 89 |  |  |  |  | 98 |  |
| 90 |  |  |  |  | 94.55 |  |
| 91 |  |  |  |  | 92 |  |
| 92 |  |  |  |  | 96.3 |  |
| 93 |  |  |  |  | 96.43 |  |
| 94 |  |  |  |  | 94.19 |  |
| 95 |  |  |  |  | 95.59 |  |
| 96 |  |  |  |  | 95.95 |  |
| 97 |  |  |  |  | 100 |  |
| 98 |  |  |  |  | 98.08 |  |
| 99 |  |  |  |  | 95.58 |  |
| 100 |  |  |  |  | 99.07 |  |
| 101 |  |  |  |  | 100 |  |
| 102 |  |  |  |  | 98.18 |  |
| 103 |  |  |  |  | 96.84 |  |
| 104 |  |  |  |  | 97.33 |  |
| 105 |  |  |  |  | 96.61 |  |
| 106 |  |  |  |  | 100 |  |
| 107 |  |  |  |  | 96.26 |  |

Supplementary Table S9 Novel TE insertion sites

| **Chr** | **Pos1** | **Pos2** | **TE family** |
| --- | --- | --- | --- |
| Chr1 | 670503 | 670503 | retrosat1:12:Llacaetal.1999unpublished: |
| Chr1 | 2007845 | 2007845 | osr25:210:McCarthyEM: |
| Chr1 | 6939278 | 6939278 | hopi:269:PanaudO: |
| Chr1 | 8235559 | 8235582 | dasheng:232:JiangN: |
| Chr1 | 8292221 | 8292221 | rn_66-340:2:A.Zuccolounpublished: |
| Chr1 | 9532042 | 9532448 | hopi:269:PanaudO: |
| Chr1 | 12724974 | 12724974 | dasheng:232:JiangN: |
| Chr1 | 13684790 | 13684790 | osr29:72:McCarthyEM: |
| Chr1 | 14697686 | 14697737 | hopi:269:PanaudO: |
| Chr1 | 16196103 | 16196103 | osr41:49:McCarthyEM: |
| Chr1 | 16441941 | 16441941 | rn_20-10:1:A.Zuccolounpublished: |
| Chr1 | 17699530 | 17699531 | rn_16-305:6:A.Zuccolounpublished: |
| Chr1 | 18545576 | 18545576 | dasheng:232:JiangN: |
| Chr1 | 19225918 | 19225918 | hopi:269:PanaudO: |
| Chr1 | 19553380 | 19553380 | dasheng:232:JiangN: |
| Chr1 | 20184297 | 20190626 | dasheng:232:JiangN: |
| Chr1 | 21235564 | 21235564 | hopi:269:PanaudO: |
| Chr1 | 22849360 | 22849360 | hopi:269:PanaudO: |
| Chr1 | 24389854 | 24389936 | osr9:2:McCarthyEM: |
| Chr1 | 26755912 | 26755912 | dasheng:232:JiangN: |
| Chr1 | 29356185 | 29356185 | hopi:269:PanaudO: |
| Chr1 | 30433435 | 30433435 | hopi:269:PanaudO: |
| Chr1 | 36713565 | 36713577 | hopi:269:PanaudO: |
| Chr1 | 41610852 | 41610852 | rn_44-393:4:A.Zuccolounpublished: |
| Chr1 | 41685661 | 41685661 | rn_455-235:1:A.Zuccolounpublished: |
| Chr1 | 41996610 | 41996610 | osr14:2:McCarthyEM: |
| Chr10 | 753383 | 753421 | hopi:269:PanaudO: |
| Chr10 | 5476610 | 5482778 | dasheng:232:JiangN: |
| Chr10 | 5881713 | 5881722 | osr29:72:McCarthyEM: |
| Chr10 | 6051701 | 6051701 | dasheng:232:JiangN: |
| Chr10 | 6186519 | 6186525 | dasheng:232:JiangN: |
| Chr10 | 11644910 | 11644910 | osr14:2:McCarthyEM: |
| Chr10 | 12452223 | 12452262 | dasheng:232:JiangN: |
| Chr10 | 13407602 | 13407623 | osr41:49:McCarthyEM: |
| Chr10 | 16435986 | 16435986 | dasheng:232:JiangN: |
| Chr10 | 16539022 | 16539022 | rn_206-352:19:A.Zuccolounpublished: |
| Chr10 | 16936189 | 16936969 | osr40:115:McCarthyEM: |
| Chr10 | 19864902 | 19864970 | rn_171-247:3:A.Zuccolounpublished: |
| Chr10 | 21494270 | 21494270 | retrosat1:12:Llacaetal.1999unpublished: |
| Chr11 | 1221255 | 1221255 | dasheng:232:JiangN: |
| Chr11 | 4886133 | 4886144 | hopi:269:PanaudO: |
| Chr11 | 6406162 | 6406181 | rn_402-75:2:A.Zuccolounpublished: |
| Chr11 | 9335887 | 9335887 | hopi:269:PanaudO: |
| Chr11 | 9746074 | 9746129 | rn_359-198:1:A.Zuccolounpublished: |
| Chr11 | 11294763 | 11294793 | echidne_osj:11:GuyotR.unpublished: |
| Chr11 | 11827760 | 11827760 | osr29:72:McCarthyEM: |
| Chr11 | 16006901 | 16006901 | osr41:49:McCarthyEM: |
| Chr11 | 16624047 | 16624048 | dasheng:232:JiangN: |
| Chr11 | 17228738 | 17228744 | osr14:2:McCarthyEM: |
| Chr11 | 18601690 | 18602155 | dasheng:232:JiangN: |
| Chr11 | 21007005 | 21007005 | osr41:49:McCarthyEM: |
| Chr11 | 21625298 | 21625298 | osr40:115:McCarthyEM: |
| Chr11 | 23565714 | 23565889 | osr6:2:McCarthyEM: |
| Chr11 | 24452231 | 24452552 | dasheng:232:JiangN: |
| Chr11 | 27276066 | 27276139 | hamster:4:GuyotR.unpublished: |
| Chr11 | 27779079 | 27785108 | rn_171-247:3:A.Zuccolounpublished: |
| Chr11 | 27916786 | 27917667 | rn_18-313:4:A.Zuccolounpublished: |
| Chr11 | 29009592 | 29010326 | osr14:2:McCarthyEM: |
| Chr12 | 45809 | 50039 | rn_500-252:2:A.Zuccolounpublished: |
| Chr12 | 3425915 | 3426697 | osr9:2:McCarthyEM: |
| Chr12 | 5448618 | 5448618 | rn_85-323:6:A.Zuccolounpublished: |
| Chr12 | 6607934 | 6607934 | osr29:72:McCarthyEM: |
| Chr12 | 7754166 | 7754196 | rn_206-352:19:A.Zuccolounpublished: |
| Chr12 | 8548155 | 8548155 | dasheng:232:JiangN: |
| Chr12 | 10087794 | 10087794 | osr29:72:McCarthyEM: |
| Chr12 | 10264479 | 10264479 | osr9:2:McCarthyEM: |
| Chr12 | 11569436 | 11575369 | dasheng:232:JiangN: |
| Chr12 | 12832395 | 12832430 | dasheng:232:JiangN: |
| Chr12 | 13631772 | 13631779 | osr29:72:McCarthyEM: |
| Chr12 | 16218017 | 16218017 | echidne_osj:11:GuyotR.unpublished: |
| Chr12 | 17507877 | 17508619 | echidne_osj:11:GuyotR.unpublished: |
| Chr12 | 19843620 | 19844374 | hopi:269:PanaudO: |
| Chr12 | 20330635 | 20330635 | dasheng:232:JiangN: |
| Chr12 | 20399010 | 20399020 | dasheng:232:JiangN: |
| Chr12 | 20997294 | 20997294 | osr9:2:McCarthyEM: |
| Chr12 | 22547105 | 22547122 | hopi:269:PanaudO: |
| Chr2 | 3070176 | 3070176 | osr12:2:McCarthyEM: |
| Chr2 | 4155746 | 4155746 | hopi:269:PanaudO: |
| Chr2 | 5961177 | 5961177 | dasheng:232:JiangN: |
| Chr2 | 7225055 | 7225055 | dasheng:232:JiangN: |
| Chr2 | 8330172 | 8330172 | echidne_osj:11:GuyotR.unpublished: |
| Chr2 | 8611566 | 8611566 | rn_112-64:1:A.Zuccolounpublished: |
| Chr2 | 9594862 | 9594883 | hopi:269:PanaudO: |
| Chr2 | 9743271 | 9746965 | hopi:269:PanaudO: |
| Chr2 | 10647994 | 10647994 | rn_85-323:6:A.Zuccolounpublished: |
| Chr2 | 14177530 | 14177530 | dasheng:232:JiangN: |
| Chr2 | 14414680 | 14414680 | rn_15-6:1:A.Zuccolounpublished: |
| Chr2 | 15897524 | 15897524 | osr9:2:McCarthyEM: |
| Chr2 | 16135248 | 16135248 | rn_364-201:1:A.Zuccolounpublished: |
| Chr2 | 16236919 | 16236919 | dasheng:232:JiangN: |
| Chr2 | 18865208 | 18866010 | rire5:8:: |
| Chr2 | 22308633 | 22308633 | echidne_osj:11:GuyotR.unpublished: |
| Chr2 | 25930954 | 25930954 | osr17:11:McCarthyEM: |
| Chr2 | 28064562 | 28064578 | hopi:269:PanaudO: |
| Chr2 | 30239723 | 30239723 | dasheng:232:JiangN: |
| Chr2 | 30259460 | 30259617 | dasheng:232:JiangN: |
| Chr2 | 31390115 | 31390252 | rn_4-189:7:A.Zuccolounpublished: |
| Chr2 | 32079070 | 32079070 | osr4:18:osr4: |
| Chr3 | 1392221 | 1392223 | retrosat1:12:Llacaetal.1999unpublished: |
| Chr3 | 2259074 | 2259074 | dasheng:232:JiangN: |
| Chr3 | 3574593 | 3574593 | dasheng:232:JiangN: |
| Chr3 | 3594411 | 3595291 | dasheng:232:JiangN: |
| Chr3 | 6350936 | 6350936 | rn_219-129:4:A.Zuccolounpublished: |
| Chr3 | 8334006 | 8334012 | dasheng:232:JiangN: |
| Chr3 | 12753200 | 12753203 | dasheng:232:JiangN: |
| Chr3 | 13264816 | 13264837 | osr17:11:McCarthyEM: |
| Chr3 | 14978377 | 14978388 | dasheng:232:JiangN: |
| Chr3 | 15033888 | 15039230 | rn_110-271:4:A.Zuccolounpublished: |
| Chr3 | 16447508 | 16447536 | rn_206-352:19:A.Zuccolounpublished: |
| Chr3 | 16896645 | 16896645 | dasheng:232:JiangN: |
| Chr3 | 18942799 | 18943600 | rn_83-55:4:A.Zuccolounpublished: |
| Chr3 | 20632800 | 20632800 | echidne_osj:11:GuyotR.unpublished: |
| Chr3 | 20803964 | 20804002 | osr29:72:McCarthyEM: |
| Chr3 | 21258848 | 21258848 | dasheng:232:JiangN: |
| Chr3 | 24612861 | 24612861 | rn_505-256:1:A.Zuccolounpublished: |
| Chr3 | 26433594 | 26433594 | osr12:2:McCarthyEM: |
| Chr3 | 26806279 | 26806279 | osr5:5:McCarthyEM: |
| Chr3 | 33828058 | 33828058 | hopi:269:PanaudO: |
| Chr4 | 2547059 | 2547059 | dasheng:232:JiangN: |
| Chr4 | 2675016 | 2675030 | osr29:72:McCarthyEM: |
| Chr4 | 2837082 | 2837082 | rn_573-292:3:A.Zuccolounpublished: |
| Chr4 | 2948561 | 2948561 | osr29:72:McCarthyEM: |
| Chr4 | 4527727 | 4527727 | hopi:269:PanaudO: |
| Chr4 | 4539577 | 4539577 | hopi:269:PanaudO: |
| Chr4 | 6657156 | 6657156 | osr15:21:McCarthyEM: |
| Chr4 | 7418411 | 7418411 | hopi:269:PanaudO: |
| Chr4 | 7859383 | 7866718 | dasheng:232:JiangN: |
| Chr4 | 8250370 | 8250370 | osr29:72:McCarthyEM: |
| Chr4 | 8397533 | 8397533 | hopi:269:PanaudO: |
| Chr4 | 8421078 | 8421079 | dasheng:232:JiangN: |
| Chr4 | 8884914 | 8885704 | osr29:72:McCarthyEM: |
| Chr4 | 10090984 | 10090984 | rn_129-74:1:A.Zuccolounpublished: |
| Chr4 | 11784399 | 11784399 | osr4:18:osr4: |
| Chr4 | 11806257 | 11806260 | osr4:18:osr4: |
| Chr4 | 13792896 | 13792896 | dasheng:232:JiangN: |
| Chr4 | 16860198 | 16860216 | rn_180-106:1:A.Zuccolounpublished: |
| Chr4 | 16902154 | 16902154 | osr41:49:McCarthyEM: |
| Chr4 | 19882630 | 19883415 | osr14:2:McCarthyEM: |
| Chr4 | 21914675 | 21914829 | hopi:269:PanaudO: |
| Chr4 | 22067301 | 22067301 | rn_83-55:4:A.Zuccolounpublished: |
| Chr4 | 28329524 | 28329524 | hopi:269:PanaudO: |
| Chr4 | 29845885 | 29845905 | rn_206-352:19:A.Zuccolounpublished: |
| Chr4 | 32667860 | 32667860 | rn_206-352:19:A.Zuccolounpublished: |
| Chr5 | 404445 | 404445 | hopi:269:PanaudO: |
| Chr5 | 2215143 | 2215647 | dasheng:232:JiangN: |
| Chr5 | 2627486 | 2628120 | rn_16-305:6:A.Zuccolounpublished: |
| Chr5 | 5721723 | 5721729 | hopi:269:PanaudO: |
| Chr5 | 6018729 | 6018746 | hopi:269:PanaudO: |
| Chr5 | 6033898 | 6033898 | rn_223-132:2:A.Zuccolounpublished: |
| Chr5 | 7099795 | 7099795 | osr41:49:McCarthyEM: |
| Chr5 | 8929555 | 8929555 | osr29:72:McCarthyEM: |
| Chr5 | 9578871 | 9578894 | osr29:72:McCarthyEM: |
| Chr5 | 10592976 | 10592986 | dasheng:232:JiangN: |
| Chr5 | 10740107 | 10740107 | hopi:269:PanaudO: |
| Chr5 | 12257902 | 12257902 | osr17:11:McCarthyEM: |
| Chr5 | 12768866 | 12768920 | rn_129-74:1:A.Zuccolounpublished: |
| Chr5 | 12914346 | 12914346 | rn_211-124:2:A.Zuccolounpublished: |
| Chr5 | 14270430 | 14270430 | rn_129-74:1:A.Zuccolounpublished: |
| Chr5 | 15097792 | 15097792 | echidne_osj:11:GuyotR.unpublished: |
| Chr5 | 15419970 | 15420764 | dasheng:232:JiangN: |
| Chr5 | 16564593 | 16564619 | echidne_osj:11:GuyotR.unpublished: |
| Chr5 | 17407059 | 17407135 | dasheng:232:JiangN: |
| Chr5 | 17545553 | 17545561 | retrosat1:12:Llacaetal.1999unpublished: |
| Chr5 | 17565678 | 17565678 | dasheng:232:JiangN: |
| Chr5 | 18178814 | 18178841 | osr4:18:osr4: |
| Chr5 | 26423498 | 26423498 | dasheng:232:JiangN: |
| Chr6 | 340923 | 340923 | hopi:269:PanaudO: |
| Chr6 | 958374 | 959112 | dasheng:232:JiangN: |
| Chr6 | 4450524 | 4450524 | echidne_osj:11:GuyotR.unpublished: |
| Chr6 | 8481200 | 8481200 | dasheng:232:JiangN: |
| Chr6 | 9811278 | 9811278 | osr17:11:McCarthyEM: |
| Chr6 | 10461187 | 10461187 | dasheng:232:JiangN: |
| Chr6 | 10596225 | 10596225 | dasheng:232:JiangN: |
| Chr6 | 10777538 | 10777538 | dasheng:232:JiangN: |
| Chr6 | 11175064 | 11175455 | dasheng:232:JiangN: |
| Chr6 | 12029977 | 12029977 | dasheng:232:JiangN: |
| Chr6 | 12495631 | 12503990 | dasheng:232:JiangN: |
| Chr6 | 13233655 | 13233655 | echidne_osj:11:GuyotR.unpublished: |
| Chr6 | 13679372 | 13679373 | osr43:1:McCarthyEM: |
| Chr6 | 14540333 | 14540380 | rn_129-74:1:A.Zuccolounpublished: |
| Chr6 | 16761467 | 16761483 | dasheng:232:JiangN: |
| Chr6 | 18373846 | 18373846 | retrosat1:12:Llacaetal.1999unpublished: |
| Chr6 | 19374840 | 19374840 | retrosat1:12:Llacaetal.1999unpublished: |
| Chr6 | 20520897 | 20520955 | rire10:23:Wang,R: |
| Chr6 | 21380603 | 21381013 | dasheng:232:JiangN: |
| Chr6 | 23067158 | 23067158 | dasheng:232:JiangN: |
| Chr6 | 24121707 | 24121707 | hopi:269:PanaudO: |
| Chr6 | 25469496 | 25469496 | echidne_osj:11:GuyotR.unpublished: |
| Chr6 | 25780397 | 25780397 | dasheng:232:JiangN: |
| Chr6 | 27183109 | 27183138 | hopi:269:PanaudO: |
| Chr6 | 30083637 | 30083637 | rn_208-123:2:A.Zuccolounpublished: |
| Chr7 | 938248 | 938248 | osr15:21:McCarthyEM: |
| Chr7 | 1500744 | 1500767 | rn_21-12:3:A.Zuccolounpublished: |
| Chr7 | 2443261 | 2443261 | rn_200-117:1:A.Zuccolounpublished: |
| Chr7 | 3195737 | 3195737 | rn_129-74:1:A.Zuccolounpublished: |
| Chr7 | 4627127 | 4627127 | osr41:49:McCarthyEM: |
| Chr7 | 5959752 | 5959752 | osr17:11:McCarthyEM: |
| Chr7 | 6805819 | 6806416 | rn_315-194:2:A.Zuccolounpublished: |
| Chr7 | 8719965 | 8719965 | osr17:11:McCarthyEM: |
| Chr7 | 8973563 | 8973563 | echidne_osj:11:GuyotR.unpublished: |
| Chr7 | 9260783 | 9261380 | dasheng:232:JiangN: |
| Chr7 | 9539208 | 9539219 | rn_206-352:19:A.Zuccolounpublished: |
| Chr7 | 9613207 | 9613207 | dasheng:232:JiangN: |
| Chr7 | 11297848 | 11297858 | osr9:2:McCarthyEM: |
| Chr7 | 11519256 | 11519256 | osr40:115:McCarthyEM: |
| Chr7 | 12943730 | 12943739 | osr29:72:McCarthyEM: |
| Chr7 | 14047664 | 14047664 | rn_106-362:1:A.Zuccolounpublished: |
| Chr7 | 14123305 | 14123305 | rn_129-74:1:A.Zuccolounpublished: |
| Chr7 | 16121642 | 16121666 | dasheng:232:JiangN: |
| Chr7 | 16607763 | 16607790 | rn_206-352:19:A.Zuccolounpublished: |
| Chr7 | 17112659 | 17112684 | dasheng:232:JiangN: |
| Chr7 | 17592356 | 17593028 | rn_305-180:1:A.Zuccolounpublished: |
| Chr7 | 19724110 | 19724110 | hopi:269:PanaudO: |
| Chr7 | 19820794 | 19820802 | echidne_osj:11:GuyotR.unpublished: |
| Chr7 | 21098672 | 21098672 | dasheng:232:JiangN: |
| Chr7 | 22363820 | 22364560 | rire5:8:: |
| Chr7 | 22747913 | 22747925 | echidne_osj:11:GuyotR.unpublished: |
| Chr7 | 22771370 | 22771402 | dasheng:232:JiangN: |
| Chr7 | 23539559 | 23539559 | osr14:2:McCarthyEM: |
| Chr7 | 23745461 | 23745461 | osr17:11:McCarthyEM: |
| Chr7 | 24840813 | 24840813 | hopi:269:PanaudO: |
| Chr7 | 25477953 | 25477953 | echidne_osj:11:GuyotR.unpublished: |
| Chr8 | 2634016 | 2634016 | rn_406-217:1:A.Zuccolounpublished: |
| Chr8 | 2743028 | 2743028 | dasheng:232:JiangN: |
| Chr8 | 5313221 | 5313221 | hopi:269:PanaudO: |
| Chr8 | 6843397 | 6843408 | rn_503-254:1:A.Zuccolounpublished: |
| Chr8 | 7083272 | 7083272 | rn_356-197:2:A.Zuccolounpublished: |
| Chr8 | 7511709 | 7511709 | dasheng:232:JiangN: |
| Chr8 | 8013471 | 8013495 | osr29:72:McCarthyEM: |
| Chr8 | 8777159 | 8777920 | dasheng:232:JiangN: |
| Chr8 | 9213453 | 9218462 | rn_360-199:1:A.Zuccolounpublished: |
| Chr8 | 9573996 | 9573996 | dasheng:232:JiangN: |
| Chr8 | 10019346 | 10019365 | osr9:2:McCarthyEM: |
| Chr8 | 10437445 | 10437455 | osr29:72:McCarthyEM: |
| Chr8 | 11647435 | 11653484 | hopi:269:PanaudO: |
| Chr8 | 12388152 | 12388152 | osr43:1:McCarthyEM: |
| Chr8 | 13014998 | 13014998 | dasheng:232:JiangN: |
| Chr8 | 13468788 | 13468788 | rn_363-200:1:A.Zuccolounpublished: |
| Chr8 | 14012784 | 14012784 | rn_366-202:1:A.Zuccolounpublished: |
| Chr8 | 15568142 | 15568899 | rn_206-352:19:A.Zuccolounpublished: |
| Chr8 | 16012707 | 16012707 | osr29:72:McCarthyEM: |
| Chr8 | 19247951 | 19247976 | echidne_osj:11:GuyotR.unpublished: |
| Chr8 | 23767788 | 23767788 | retrosat1:12:Llacaetal.1999unpublished: |
| Chr8 | 23955791 | 23955791 | hopi:269:PanaudO: |
| Chr8 | 25674127 | 25674127 | osr4:18:osr4: |
| Chr8 | 25732290 | 25732290 | osr14:2:McCarthyEM: |
| Chr8 | 25807361 | 25807365 | hopi:269:PanaudO: |
| Chr8 | 26489758 | 26489761 | dasheng:232:JiangN: |
| Chr8 | 26621737 | 26621737 | hopi:269:PanaudO: |
| Chr8 | 28416619 | 28417395 | dasheng:232:JiangN: |
| Chr9 | 2661700 | 2661752 | hopi:269:PanaudO: |
| Chr9 | 4981901 | 4987683 | dasheng:232:JiangN: |
| Chr9 | 5475179 | 5475179 | hopi:269:PanaudO: |
| Chr9 | 6424633 | 6424633 | hopi:269:PanaudO: |
| Chr9 | 7482477 | 7482477 | rn_21-12:3:A.Zuccolounpublished: |
| Chr9 | 7585093 | 7585093 | osr41:49:McCarthyEM: |
| Chr9 | 9066267 | 9066267 | osr14:2:McCarthyEM: |
| Chr9 | 10985128 | 10985147 | hopi:269:PanaudO: |
| Chr9 | 11093666 | 11093666 | rn_106-362:1:A.Zuccolounpublished: |
| Chr9 | 11971752 | 11971752 | dasheng:232:JiangN: |
| Chr9 | 14745575 | 14745575 | osr42:1:McCarthyEM: |
| Chr9 | 15856568 | 15856568 | rn_265-150:1:A.Zuccolounpublished: |
| Chr9 | 16240859 | 16240864 | dasheng:232:JiangN: |
| Chr9 | 19164545 | 19164545 | dasheng:232:JiangN: |
| Chr9 | 19529779 | 19529779 | hopi:269:PanaudO: |
| Chr9 | 21587702 | 21587710 | osr9:2:McCarthyEM: |

Table S10 Table S10. Candidate differentially expressed genes between WT and HPM identified by microarray analysis (fold change ≥ 2, unadjusted P < 0.05; 1,079 probes; FDR values obtained by Benjamini–Hochberg correction are given)

| **Probeset ID** | **Fold-Change(HPM vs. WT)** | **Transcript Assignments** | **p-value(Group)** | **FDR (Benjamini-Hochberg)** |
| --- | --- | --- | --- | --- |
| Os.10498.1.S1_at | -482.256 | AK065202 // Oryza sativa Japonica Group cDNA clone:J013002F01, full insert seque | 7.83E-08 | 0.0000384 |
| Os.7732.1.S1_at | -293.492 | AF309380 // Oryza sativa (japonica cultivar-group) clone C2421_1A putative gluta | 0.000016 | 0.004143 |
| Os.5774.1.S1_at | -188.785 | AK242732 // Oryza sativa Japonica Group cDNA, clone: J090047M16, full insert seq | 0.0000861 | 0.01633 |
| Os.27780.2.S1_a_at | -128.292 | AK064891 // Oryza sativa Japonica Group cDNA clone:J013000K19, full insert seque | 0.0000206 | 0.004143 |
| Os.38995.1.S1_at | -107.756 | LOC_Os03g26229.1 // 40S ribosomal protein S9-1, putative, expressed // rgap // 0 | 0.0000462 | 0.006571 |
| Os.26976.1.A1_at | -89.3535 | CB631211 // /DB_XREF=OSIIEb08J17.r /CLONE=OSIIEb08J17 // gb // --- // --- | 0.000182686 | 0.01633 |
| OsAffx.30674.1.S1_at | -85.2231 | AK058401 // Oryza sativa Japonica Group cDNA clone:001-015-B06, full insert sequ | 0.0000335 | 0.01214 |
| Os.27780.2.S2_a_at | -61.57 | AK064891 // Oryza sativa Japonica Group cDNA clone:J013000K19, full insert seque | 0.0000473 | 0.01018 |
| Os.26910.1.A1_at | -61.0403 | AB462325 // Oryza sativa Japonica Group Pikm2-TS mRNA for NBS-LRR class disease | 0.0000078 | 0.004143 |
| OsAffx.28375.1.S1_at | -43.9239 | AK242778 // Oryza sativa Japonica Group cDNA, clone: J090054J19, full insert seq | 0.0000198 | 0.004143 |
| Os.52807.1.S1_at | -31.0777 | AK069391 // Oryza sativa Japonica Group cDNA clone:J023012L02, full insert seque | 0.0000368 | 0.006026 |
| OsAffx.28375.1.S1_s_at | -16.8912 | LOC_Os07g10120.1 // expressed protein // rgap // 11 // --- | 0.000397853 | 0.02124 |
| OsAffx.5795.1.S1_at | -16.79 | LOC_Os08g12680.1 // zinc finger domain, LSD1 subclass family protein, expressed | 0.0424307 | 0.2443 |
| Os.27343.1.A1_at | -14.0186 | BGIOSSBCE025181.1 // --- // bgi_ris // 0 // chr11:22911515-22912422(-) /// LOC_O | 0.0000703 | 0.01018 |
| Os.46510.1.S1_at | -13.7502 | AK069829 // Oryza sativa Japonica Group cDNA clone:J023028J10, full insert seque | 0.00058862 | 0.0287 |
| Os.12222.1.S1_a_at | -13.6129 | AK062244 // Oryza sativa Japonica Group cDNA clone:001-047-F02, full insert sequ | 0.00000696 | 0.02124 |
| Os.18191.1.S1_at | -12.4971 | CT832272 // Oryza sativa (indica cultivar-group) cDNA clone:OSIGCRA115O04, full | 0.0000106 | 0.02828 |
| Os.50202.1.S1_at | -12.2248 | AK060993 // Oryza sativa Japonica Group cDNA clone:006-203-D08, full insert sequ | 0.00149305 | 0.09576 |
| Os.46591.1.S1_at | -11.6465 | LOC_Os10g35530.1 // hydrolase, alpha/beta fold family domain containing protein, | 0.0000153 | 0.004143 |
| Os.46523.2.S1_at | -11.5766 | AK069346 // Oryza sativa Japonica Group cDNA clone:J023010E21, full insert seque | 0.000624759 | 0.02291 |
| Os.46127.1.S1_at | -11.4516 | BGIOSSBCE030322.1 // --- // bgi_ris // 10 // --- /// LOC_Os10g35260.1 // Rf1, mi | 0.000174648 | 0.01865 |
| Os.46057.1.S1_at | -10.7308 | AK101819 // Oryza sativa Japonica Group cDNA clone:J033067E15, full insert seque | 0.0000929 | 0.01211 |
| Os.53674.1.S1_at | -10.324 | LOC_Os11g39350.1 // expressed protein // rgap // 11 // --- /// LOC_Os11g39350.2 | 0.000442571 | 0.02124 |
| OsAffx.31246.1.S1_at | -9.29499 | LOC_Os11g31500.1 // ATP binding protein, putative // rgap // 11 // --- /// NM_00 | 0.000460278 | 0.02124 |
| Os.24933.1.S1_at | -7.62682 | AB462324 // Oryza sativa Japonica Group Pikm1-TS mRNA for NBS-LRR class disease | 0.00138692 | 0.05591 |
| Os.46799.1.S1_at | -7.61186 | AK102374 // Oryza sativa Japonica Group cDNA clone:J033091N12, full insert seque | 0.0000161 | 0.01214 |
| Os.46591.1.S1_x_at | -7.6016 | LOC_Os10g35530.1 // hydrolase, alpha/beta fold family domain containing protein, | 0.0000294 | 0.01578 |
| Os.40210.1.S1_at | -7.42835 | AK241980 // Oryza sativa Japonica Group cDNA, clone: J075097J20, full insert seq | 0.0000783 | 0.01578 |
| Os.9251.2.S1_at | -7.3567 | LOC_Os10g35960.2 // NAD-dependent malic enzyme, mitochondrial precursor, putativ | 0.00101815 | 0.0272 |
| Os.3571.2.S1_s_at | -7.04673 | AK121184 // Oryza sativa Japonica Group cDNA clone:J023084M13, full insert seque | 0.000120056 | 0.03449 |
| Os.46799.1.S2_at | -6.82652 | NM_001071610 // Oryza sativa (japonica cultivar-group) Os10g0518400 (Os10g051840 | 0.000168657 | 0.01633 |
| Os.31723.1.S1_at | -6.74907 | AK106856 // Oryza sativa Japonica Group cDNA clone:002-118-B09, full insert sequ | 0.0018051 | 0.03259 |
| Os.46523.3.S1_x_at | -6.63998 | AK069346 // Oryza sativa Japonica Group cDNA clone:J023010E21, full insert seque | 0.000133857 | 0.01578 |
| OsAffx.28753.1.S1_at | -6.58438 | BGIOSSFCE023585.1 // --- // bgi_ris // 11 // --- /// LOC_Os07g34750.1 // express | 0.000358427 | 0.03147 |
| Os.50834.1.S1_at | -6.51996 | AK058864 // Oryza sativa Japonica Group cDNA clone:001-005-H10, full insert sequ | 0.0000361 | 0.01593 |
| Os.46654.1.S1_at | -5.57694 | LOC_Os10g35210.1 // expressed protein // rgap // 9 // --- | 0.000938045 | 0.03931 |
| OsAffx.6388.1.A1_x_at | -5.4839 | BGIOSSBCE012003.1 // --- // bgi_ris // 9 // --- /// BGIOSSBCE027804.1 // --- // | 0.00398297 | 0.07545 |
| Os.39845.2.A1_at | -5.32694 | AK241163 // Oryza sativa Japonica Group cDNA, clone: J065116A07, full insert seq | 0.000135888 | 0.05224 |
| Os.42235.1.S1_at | -5.17201 | BGIOSSBCE030546.1 // --- // bgi_ris // 0 // chr10:20580945-20587901(-) /// BGIOS | 0.0000815 | 0.01578 |
| Os.51588.1.S1_x_at | -5.10461 | LOC_Os07g01300.1 // expressed protein // rgap // 11 // --- /// NM_001065221 // O | 0.000584635 | 0.07431 |
| OsAffx.30041.1.S1_at | -5.06131 | AK240779 // Oryza sativa Japonica Group cDNA, clone: J053075F05, full insert seq | 0.00532068 | 0.04652 |
| Os.53137.1.S1_at | -5.02055 | AK070975 // Oryza sativa Japonica Group cDNA clone:J023073F15, full insert seque | 0.00932436 | 0.05149 |
| Os.17052.1.S1_s_at | -4.93577 | AK100611 // Oryza sativa Japonica Group cDNA clone:J023107L23, full insert seque | 0.00445743 | 0.06657 |
| Os.51588.1.S1_at | -4.86767 | LOC_Os07g01300.1 // expressed protein // rgap // 11 // --- /// NM_001065221 // O | 0.0014975 | 0.06924 |
| Os.49984.1.S1_at | -4.78768 | AK119484 // Oryza sativa Japonica Group cDNA clone:001-134-C04, full insert sequ | 0.0209889 | 0.0698 |
| Os.45974.1.A1_at | -4.77682 | CB636434 // /DB_XREF=OSJNEa01P01.r /CLONE=OSJNEa01P01 // gb // --- // --- | 0.00315336 | 0.04034 |
| Os.46591.2.S1_x_at | -4.73784 | BGIOSSBCE030345.1 // --- // bgi_ris // 10 // --- /// LOC_Os10g35530.1 // hydrola | 0.0000858 | 0.01787 |
| Os.50448.1.S1_at | -4.66888 | AK121239 // Oryza sativa Japonica Group cDNA clone:J023096E02, full insert seque | 0.0001522 | 0.06979 |
| Os.40028.1.A1_at | -4.61776 | BGIOSSBCE029689.1 // --- // bgi_ris // 0 // chr10:12778820-12782804(+) /// CT835 | 0.000261819 | 0.02007 |
| Os.49908.1.S1_at | -4.59384 | NM_001074777 // Oryza sativa (japonica cultivar-group) Os11g0605900 (Os11g060590 | 0.00309537 | 0.03629 |
| Os.57368.1.S1_at | -4.45332 | AK111390 // Oryza sativa Japonica Group cDNA clone:002-182-C04, full insert sequ | 0.00210684 | 0.0446 |
| OsAffx.8732.1.S1_at | -4.39418 | XM_354127.1 // --- // gb // --- // --- | 0.00527944 | 0.0482 |
| OsAffx.31381.1.S1_at | -4.36149 | LOC_Os11g39200.1 // hAT dimerisation domain containing protein // rgap // 11 // | 0.00113461 | 0.02741 |
| Os.53948.1.S1_at | -4.35396 | AK100970 // Oryza sativa Japonica Group cDNA clone:J023143B18, full insert seque | 0.000744146 | 0.09007 |
| Os.51487.1.S1_at | -4.34554 | AK062933 // Oryza sativa Japonica Group cDNA clone:001-109-A11, full insert sequ | 0.0023658 | 0.0725 |
| OsAffx.7237.1.S1_at | -4.3388 | BGIOSSFCE032555.1 // --- // bgi_ris // 11 // --- /// LOC_Os11g28670.1 // hypothe | 0.000459206 | 0.02124 |
| OsAffx.4700.1.S1_at | -4.12298 | BGIOSSFCE019138.1 // --- // bgi_ris // 11 // --- /// LOC_Os06g03200.1 // hypothe | 0.00050299 | 0.04018 |
| Os.53944.1.A1_s_at | -4.00207 | AK100962 // Oryza sativa Japonica Group cDNA clone:J023141M07, full insert seque | 0.00813197 | 0.09868 |
| OsAffx.31389.1.S1_at | -3.94999 | BGIOSSFCE009028.1 // --- // bgi_ris // 11 // --- /// LOC_Os11g39550.1 // Leucine | 0.000697149 | 0.02218 |
| Os.39845.3.S1_a_at | -3.91918 | AK241163 // Oryza sativa Japonica Group cDNA, clone: J065116A07, full insert seq | 0.00095921 | 0.05484 |
| Os.46510.2.S1_x_at | -3.90253 | AK069829 // Oryza sativa Japonica Group cDNA clone:J023028J10, full insert seque | 0.00183984 | 0.03481 |
| Os.20045.1.S1_at | -3.81903 | AK102826 // Oryza sativa Japonica Group cDNA clone:J033109C10, full insert seque | 0.00107253 | 0.0446 |
| Os.54295.1.S1_at | -3.79015 | LOC_Os09g26540.2 // expressed protein // rgap // 10 // --- /// NM_001069792 // O | 0.00386802 | 0.07628 |
| Os.14861.1.S1_s_at | -3.78068 | AK073060 // Oryza sativa Japonica Group cDNA clone:J033021G01, full insert seque | 0.00114872 | 0.02741 |
| OsAffx.27945.1.S1_s_at | -3.77703 | AK111390 // Oryza sativa Japonica Group cDNA clone:002-182-C04, full insert sequ | 0.00467167 | 0.05085 |
| Os.47902.1.S1_at | -3.76499 | LOC_Os11g39290.1 // Leucine Rich Repeat family protein, expressed // rgap // 9 / | 0.0000543 | 0.01593 |
| Os.6845.1.S1_at | -3.75843 | LOC_Os10g36160.1 // LTPL159 - Protease inhibitor/seed storage/LTP family protein | 0.0369587 | 0.09183 |
| Os.10180.1.S1_at | -3.75509 | AK069452 // Oryza sativa Japonica Group cDNA clone:J023019F19, full insert seque | 0.00557368 | 0.0446 |
| Os.23270.1.S1_at | -3.7325 | AK072155 // Oryza sativa Japonica Group cDNA clone:J013132P19, full insert seque | 0.00547101 | 0.05118 |
| Os.6664.1.S1_at | -3.71922 | CT829999 // Oryza sativa (indica cultivar-group) cDNA clone:OSIGCPI015F23, full | 0.00438824 | 0.04341 |
| Os.52187.1.S1_at | -3.70286 | AK065527 // Oryza sativa Japonica Group cDNA clone:J013032P14, full insert seque | 0.0149296 | 0.1123 |
| Os.56252.1.S1_at | -3.68313 | LOC_Os01g57540.1 // protein kinase, putative, expressed // rgap // 11 // --- /// | 0.00860119 | 0.05747 |
| Os.37213.1.S1_at | -3.65985 | LOC_Os07g29310.1 // OsSAUR30 - Auxin-responsive SAUR gene family member, express | 0.00281186 | 0.03614 |
| Os.42235.1.S1_x_at | -3.65325 | BGIOSSBCE030546.1 // --- // bgi_ris // 0 // chr10:20580945-20587901(-) /// BGIOS | 0.00053554 | 0.03825 |
| Os.2493.2.S1_x_at | -3.53606 | LOC_Os10g33230.1 // RNA recognition motif containing protein, putative, expresse | 0.00140602 | 0.04789 |
| Os.54596.1.S1_x_at | -3.51715 | AK106103 // Oryza sativa Japonica Group cDNA clone:001-207-C08, full insert sequ | 0.004867 | 0.08012 |
| Os.17522.1.S1_s_at | -3.51281 | LOC_Os10g37290.1 // DEFL6 - Defensin and Defensin-like DEFL family, expressed // | 0.000963796 | 0.02613 |
| OsAffx.6407.1.S1_at | -3.42788 | AK100611 // Oryza sativa Japonica Group cDNA clone:J023107L23, full insert seque | 0.0161978 | 0.07189 |
| OsAffx.16183.1.S1_at | -3.3831 | LOC_Os07g09950.1 // OsFBX222 - F-box domain containing protein, expressed // rga | 0.00968855 | 0.05404 |
| Os.53606.1.S1_at | -3.36164 | AK073534 // Oryza sativa Japonica Group cDNA clone:J033042E22, full insert seque | 0.00458696 | 0.04018 |
| Os.22665.1.S1_at | -3.35278 | AY425991 // Oryza sativa (japonica cultivar-group) homeodomain leucine-zipper pr | 0.00583633 | 0.06634 |
| Os.19028.1.S1_at | -3.33597 | AK242269 // Oryza sativa Japonica Group cDNA, clone: J075186H09, full insert seq | 0.010869 | 0.1374 |
| OsAffx.27382.1.S1_at | -3.31116 | BGIOSSBCE018552.1 // --- // bgi_ris // 11 // --- | 0.000886433 | 0.02398 |
| Os.11748.2.S2_at | -3.30098 | AK101661 // Oryza sativa Japonica Group cDNA clone:J033057K08, full insert seque | 0.00264392 | 0.03808 |
| Os.54753.1.S1_at | -3.29632 | LOC_Os06g49920.1 // expressed protein // rgap // 11 // --- /// NM_001065100 // O | 0.0147064 | 0.1132 |
| OsAffx.20044.1.S1_at | -3.29087 | BGIOSSFCE035277.1 // --- // bgi_ris // 11 // --- /// LOC_Os12g40080.1 // B3 DNA | 0.00437272 | 0.04162 |
| Os.57140.1.S1_at | -3.25554 | AK111007 // Oryza sativa Japonica Group cDNA clone:002-174-F06, full insert sequ | 0.00206901 | 0.03449 |
| Os.52949.1.S1_at | -3.24583 | AK070072 // Oryza sativa Japonica Group cDNA clone:J023043N14, full insert seque | 0.00309384 | 0.07545 |
| OsAffx.31383.1.S1_at | -3.22984 | LOC_Os11g39300.1 // conserved hypothetical protein // rgap // 11 // --- /// NM_0 | 0.00885564 | 0.1372 |
| Os.55085.1.S1_at | -3.22544 | NM_001059104 // Oryza sativa (japonica cultivar-group) Os04g0372400 (Os04g037240 | 0.00246589 | 0.03931 |
| Os.52240.1.S1_at | -3.22352 | AK065824 // Oryza sativa Japonica Group cDNA clone:J013041A20, full insert seque | 0.001471 | 0.03103 |
| OsAffx.29475.2.S1_s_at | -3.19257 | BGIOSSBCE025909.1 // --- // bgi_ris // 9 // --- /// LOC_Os08g32540.1 // cyclin, | 0.00388592 | 0.06984 |
| Os.37238.1.S1_at | -3.19123 | AB288027 // Oryza sativa Japonica Group OsHAP2A mRNA for HAP2 subunit of HAP com | 0.00946993 | 0.07431 |
| Os.51138.1.S1_at | -3.15795 | AK061082 // Oryza sativa Japonica Group cDNA clone:006-206-F07, full insert sequ | 0.00559229 | 0.04402 |
| Os.1880.1.A1_at | -3.15173 | AK068081 // Oryza sativa Japonica Group cDNA clone:J013131A19, full insert seque | 0.00162932 | 0.0421 |
| Os.52487.1.S1_at | -3.15157 | NM_001058612 // Oryza sativa (japonica cultivar-group) Os04g0126500 (Os04g012650 | 0.00579298 | 0.05427 |
| Os.50973.1.S1_at | -3.14013 | LOC_Os02g20040.1 // loricrin, putative, expressed // rgap // 11 // --- /// NM_00 | 0.0014746 | 0.02935 |
| Os.52814.1.S1_at | -3.13894 | AK069714 // Oryza sativa Japonica Group cDNA clone:J023030K16, full insert seque | 0.00537643 | 0.07315 |
| Os.17055.1.A1_a_at | -3.136 | CT828119 // Oryza sativa (indica cultivar-group) cDNA clone:OSIGCRA129O14, full | 0.000437792 | 0.04827 |
| Os.37913.1.S1_at | -3.08419 | NM_001059164 // Oryza sativa (japonica cultivar-group) Os04g0387900 (Os04g038790 | 0.000102522 | 0.01214 |
| Os.30993.1.S1_at | -3.05863 | LOC_Os07g12390.1 // expressed protein // rgap // 11 // --- /// NM_001065749 // O | 0.00259315 | 0.03614 |
| Os.34631.1.S1_at | -3.03289 | AK070693 // Oryza sativa Japonica Group cDNA clone:J023056A16, full insert seque | 0.0010506 | 0.03937 |
| Os.27357.1.A1_at | -3.02969 | AK243407 // Oryza sativa Japonica Group cDNA, clone: J100065I12, full insert seq | 0.0498545 | 0.1968 |
| Os.52172.1.S1_at | -3.02256 | AK102311 // Oryza sativa Japonica Group cDNA clone:J033090D11, full insert seque | 0.0122591 | 0.0557 |
| Os.39933.1.S1_at | -3.0197 | AK240881 // Oryza sativa Japonica Group cDNA, clone: J065027H08, full insert seq | 0.0000618 | 0.01929 |
| Os.18207.1.A1_s_at | -3.01248 | LOC_Os06g20879.1 // expressed protein // rgap // 11 // --- /// LOC_Os06g20879.2 | 0.000122735 | 0.01935 |
| Os.27244.1.A1_s_at | -3.00077 | AK072320 // Oryza sativa Japonica Group cDNA clone:J023029D20, full insert seque | 0.00103576 | 0.06238 |
| Os.1798.1.S1_at | -2.99532 | LOC_Os01g05080.1 // thylakoid lumenal protein, putative, expressed // rgap // 11 | 0.00177486 | 0.03977 |
| Os.17055.2.A1_a_at | -2.99014 | CT828119 // Oryza sativa (indica cultivar-group) cDNA clone:OSIGCRA129O14, full | 0.000288173 | 0.03825 |
| Os.4194.1.S1_at | -2.9768 | LOC_Os10g40430.1 // LTPL139 - Protease inhibitor/seed storage/LTP family protein | 0.0274221 | 0.1023 |
| Os.54084.1.S1_at | -2.97509 | AK101768 // Oryza sativa Japonica Group cDNA clone:J033064G18, full insert seque | 0.000777299 | 0.03353 |
| Os.19046.1.S1_at | -2.96643 | AK069810 // Oryza sativa Japonica Group cDNA clone:J023027G23, full insert seque | 0.00269496 | 0.04402 |
| Os.57340.1.S1_at | -2.951 | AK111331 // Oryza sativa Japonica Group cDNA clone:002-181-E04, full insert sequ | 0.0017452 | 0.03228 |
| OsAffx.31256.1.S1_at | -2.94603 | BGIOSSBCE032219.1 // --- // bgi_ris // 10 // --- /// BGIOSSFCE032735.1 // --- // | 0.0026443 | 0.04018 |
| Os.29923.1.S1_at | -2.92707 | AK109653 // Oryza sativa Japonica Group cDNA clone:002-140-D12, full insert sequ | 0.000600122 | 0.09041 |
| OsAffx.7337.1.S1_at | -2.90532 | LOC_Os11g38330.1 // ZOS11-06 - C2H2 zinc finger protein, expressed // rgap // 9 | 0.000378605 | 0.01935 |
| OsAffx.27945.1.S1_at | -2.90011 | BGIOSSFCE029933.1 // --- // bgi_ris // 11 // --- | 0.0234445 | 0.07258 |
| Os.18664.1.S1_a_at | -2.89969 | AK318559 // Oryza sativa Japonica Group cDNA, clone: J075168P06, full insert seq | 0.0259245 | 0.07694 |
| Os.54310.1.S1_at | -2.88192 | AK103254 // Oryza sativa Japonica Group cDNA clone:J033123O13, full insert seque | 0.000166613 | 0.03427 |
| Os.50848.1.S1_at | -2.86381 | AK058960 // Oryza sativa Japonica Group cDNA clone:001-020-B11, full insert sequ | 0.000827636 | 0.04199 |
| Os.54264.1.S1_at | -2.86004 | AK102912 // Oryza sativa Japonica Group cDNA clone:J033113K17, full insert seque | 0.00214796 | 0.03808 |
| Os.49164.2.S1_x_at | -2.85718 | AK064023 // Oryza sativa Japonica Group cDNA clone:001-125-A05, full insert sequ | 0.00020299 | 0.03191 |
| Os.7690.1.S1_at | -2.84891 | AK061321 // Oryza sativa Japonica Group cDNA clone:006-302-F02, full insert sequ | 0.00399182 | 0.1028 |
| Os.49439.1.S1_at | -2.84879 | LOC_Os03g46040.1 // expressed protein // rgap // 11 // --- /// NM_001057366 // O | 0.00742983 | 0.12 |
| Os.6268.1.S1_at | -2.81284 | AK070597 // Oryza sativa Japonica Group cDNA clone:J023058C13, full insert seque | 0.000692035 | 0.04703 |
| Os.54310.1.S1_s_at | -2.79289 | AK103254 // Oryza sativa Japonica Group cDNA clone:J033123O13, full insert seque | 0.00202375 | 0.04018 |
| Os.52321.1.S1_at | -2.78969 | AK066385 // Oryza sativa Japonica Group cDNA clone:J013067H21, full insert seque | 0.0149098 | 0.1249 |
| Os.8523.1.S1_at | -2.78521 | AK067827 // Oryza sativa Japonica Group cDNA clone:J013120O20, full insert seque | 0.000870718 | 0.03562 |
| Os.5818.1.S1_at | -2.78444 | AK066135 // Oryza sativa Japonica Group cDNA clone:J013053F22, full insert seque | 0.00367511 | 0.04224 |
| Os.12111.1.S1_at | -2.7743 | CT831803 // Oryza sativa (indica cultivar-group) cDNA clone:OSIGCSA008D23, full | 0.00141413 | 0.04679 |
| OsAffx.32166.1771.S1_s_at | -2.76642 | LOC_Os02g50540.1 // expressed protein // rgap // 9 // --- | 0.00238069 | 0.2207 |
| Os.24551.3.S1_at | -2.76065 | AK099976 // Oryza sativa Japonica Group cDNA clone:J013128K19, full insert seque | 0.0160055 | 0.09007 |
| Os.49697.1.S1_at | -2.74334 | AK072599 // Oryza sativa Japonica Group cDNA clone:J023127J21, full insert seque | 0.00026836 | 0.07545 |
| OsAffx.11074.1.S1_s_at | -2.72847 | AK102033 // Oryza sativa Japonica Group cDNA clone:J033081B10, full insert seque | 0.00324877 | 0.03825 |
| Os.39933.1.S1_x_at | -2.72159 | AK240881 // Oryza sativa Japonica Group cDNA, clone: J065027H08, full insert seq | 0.00307552 | 0.04115 |
| Os.14894.1.A1_at | -2.72016 | AK242083 // Oryza sativa Japonica Group cDNA, clone: J075139L02, full insert seq | 0.000288692 | 0.01865 |
| OsAffx.18534.1.S1_at | -2.7179 | BGIOSSFCE030839.1 // --- // bgi_ris // 11 // --- /// LOC_Os10g36340.2 // express | 0.000466874 | 0.08835 |
| Os.34167.2.S1_at | -2.6964 | LOC_Os01g26970.1 // expressed protein // rgap // 10 // --- /// LOC_Os01g26970.2 | 0.000388599 | 0.0255 |
| Os.55290.1.S1_at | -2.68372 | AK107808 // Oryza sativa Japonica Group cDNA clone:002-133-E10, full insert sequ | 0.000159656 | 0.0334 |
| Os.26862.1.S1_s_at | -2.68004 | AK240637 // Oryza sativa Japonica Group cDNA, clone: J013047L22, full insert seq | 0.00175698 | 0.04018 |
| Os.54839.1.A1_at | -2.67515 | CT831531 // Oryza sativa (indica cultivar-group) cDNA clone:OSIGCSN085N12, full | 0.0087667 | 0.05591 |
| Os.11106.1.S1_at | -2.67318 | AK072958 // Oryza sativa Japonica Group cDNA clone:J023149H22, full insert seque | 0.0165327 | 0.09882 |
| Os.23916.1.A1_at | -2.67153 | AK065926 // Oryza sativa Japonica Group cDNA clone:J013050P04, full insert seque | 0.00938978 | 0.05118 |
| Os.49252.1.S1_x_at | -2.65835 | AK069129 // Oryza sativa Japonica Group cDNA clone:J023007E02, full insert seque | 0.00647853 | 0.05591 |
| Os.47130.1.S1_at | -2.6529 | LOC_Os03g39910.1 // XH domain containing protein, expressed // rgap // 11 // --- | 0.00268498 | 0.03648 |
| Os.53569.1.S1_at | -2.6478 | AK073329 // Oryza sativa Japonica Group cDNA clone:J033024B22, full insert seque | 0.00294067 | 0.03825 |
| Os.1070.1.S1_at | -2.64736 | LOC_Os01g03020.1 // tRNA synthetase class I, putative, expressed // rgap // 11 / | 0.00470826 | 0.162 |
| Os.15944.1.S1_a_at | -2.64661 | AK071551 // Oryza sativa Japonica Group cDNA clone:J023106C12, full insert seque | 0.00534623 | 0.0433 |
| OsAffx.15533.1.S1_at | -2.63526 | BGIOSSBCE020031.1 // --- // bgi_ris // 11 // --- /// CT836289 // Oryza sativa (i | 0.00316893 | 0.05264 |
| Os.40388.2.S1_s_at | -2.62747 | AK066152 // Oryza sativa Japonica Group cDNA clone:J013055N04, full insert seque | 0.0144031 | 0.07636 |
| Os.12239.1.S1_at | -2.62544 | AK103890 // Oryza sativa Japonica Group cDNA clone:J033150D17, full insert seque | 0.00054702 | 0.0272 |
| OsAffx.15857.1.S1_at | -2.62388 | BGIOSSFCE021062.1 // --- // bgi_ris // 11 // --- /// LOC_Os06g40230.1 // hypothe | 0.0239254 | 0.07316 |
| Os.27236.2.A1_at | -2.61963 | LOC_Os05g05530.1 // expressed protein // rgap // 0 // chr5:2748299-2752895(-) // | 0.0238877 | 0.1057 |
| Os.10427.1.S1_a_at | -2.61415 | AK241652 // Oryza sativa Japonica Group cDNA, clone: J065191C03, full insert seq | 0.00076311 | 0.03825 |
| OsAffx.31767.1.S1_x_at | -2.61191 | AK070975 // Oryza sativa Japonica Group cDNA clone:J023073F15, full insert seque | 0.019795 | 0.0698 |
| OsAffx.27671.1.S1_at | -2.61033 | BGIOSSFCE020103.1 // --- // bgi_ris // 11 // --- /// LOC_Os06g16590.1 // hypothe | 0.0240397 | 0.07418 |
| Os.54652.1.S1_at | -2.60165 | AK106380 // Oryza sativa Japonica Group cDNA clone:002-102-D10, full insert sequ | 0.00466284 | 0.04827 |
| Os.34818.1.S1_at | -2.60071 | AK120670 // Oryza sativa Japonica Group cDNA clone:J013160F14, full insert seque | 0.000257318 | 0.02124 |
| Os.5818.1.S1_x_at | -2.60031 | AK066135 // Oryza sativa Japonica Group cDNA clone:J013053F22, full insert seque | 0.00130803 | 0.03191 |
| Os.54579.1.S1_s_at | -2.59554 | CT833721 // Oryza sativa (indica cultivar-group) cDNA clone:OSIGCRA115E19, full | 0.0325894 | 0.08423 |
| OsAffx.18825.1.S1_x_at | -2.59513 | AK242269 // Oryza sativa Japonica Group cDNA, clone: J075186H09, full insert seq | 0.0299995 | 0.1578 |
| Os.24611.1.S1_at | -2.59472 | AK067651 // Oryza sativa Japonica Group cDNA clone:J013113P22, full insert seque | 0.00875286 | 0.05085 |
| OsAffx.7520.1.S1_at | -2.57821 | LOC_Os12g08020.1 // hypothetical protein // rgap // 11 // --- /// NM_001072845 / | 0.0206522 | 0.0691 |
| Os.11969.1.S1_at | -2.57533 | LOC_Os05g01530.1 // expressed protein // rgap // 11 // --- | 0.000578176 | 0.02124 |
| Os.31837.1.A1_at | -2.56422 | AK106659 // Oryza sativa Japonica Group cDNA clone:002-113-G10, full insert sequ | 0.00062717 | 0.03427 |
| OsAffx.15233.1.S1_at | -2.56089 | AK240881 // Oryza sativa Japonica Group cDNA, clone: J065027H08, full insert seq | 0.00124278 | 0.02774 |
| Os.50255.1.S1_at | -2.55893 | AK120346 // Oryza sativa Japonica Group cDNA clone:J013063M12, full insert seque | 0.000226003 | 0.0297 |
| Os.57319.1.S1_at | -2.55586 | LOC_Os12g24084.1 // expressed protein // rgap // 11 // --- /// NM_001073204 // O | 0.00112919 | 0.03931 |
| Os.50426.1.S1_at | -2.55358 | AK121159 // Oryza sativa Japonica Group cDNA clone:J023079N03, full insert seque | 0.0022338 | 0.07637 |
| OsAffx.15233.1.S1_x_at | -2.54696 | AK240881 // Oryza sativa Japonica Group cDNA, clone: J065027H08, full insert seq | 0.00068755 | 0.02636 |
| Os.18482.1.S1_at | -2.54224 | LOC_Os06g42164.1 // expressed protein // rgap // 11 // --- | 0.00185543 | 0.03859 |
| Os.37954.1.S1_at | -2.5397 | AK103749 // Oryza sativa Japonica Group cDNA clone:J033142I04, full insert seque | 0.000761669 | 0.0251 |
| Os.26992.1.S1_at | -2.53456 | AK100303 // Oryza sativa Japonica Group cDNA clone:J023078B22, full insert seque | 0.0118136 | 0.09496 |
| Os.8456.1.S1_at | -2.52344 | LOC_Os05g10210.1 // HAD superfamily phosphatase, putative, expressed // rgap // | 0.0372981 | 0.1465 |
| Os.18630.1.S1_at | -2.52014 | AK068659 // Oryza sativa Japonica Group cDNA clone:J013161J11, full insert seque | 0.0160311 | 0.06223 |
| Os.53944.1.A1_at | -2.50956 | AK100962 // Oryza sativa Japonica Group cDNA clone:J023141M07, full insert seque | 0.0110926 | 0.05673 |
| Os.11231.1.S1_at | -2.50786 | LOC_Os05g27090.1 // basic helix-loop-helix domain containing protein, expressed | 0.00204245 | 0.03825 |
| Os.49908.2.A1_at | -2.50612 | CT831546 // Oryza sativa (indica cultivar-group) cDNA clone:OSIGCEA002L02, full | 0.0000528 | 0.01593 |
| Os.49283.1.S1_at | -2.50257 | LOC_Os02g15540.1 // expressed protein // rgap // 11 // --- /// NM_001052992 // O | 0.000431017 | 0.03614 |
| Os.10564.1.A1_at | -2.49933 | AU078002 // /DB_XREF=AU078002 /CLONE=g137c7 // gb // --- // --- | 0.00135914 | 0.0334 |
| Os.56237.1.S1_at | -2.49692 | AK109669 // Oryza sativa Japonica Group cDNA clone:002-145-A09, full insert sequ | 0.00363022 | 0.04018 |
| Os.9485.1.S1_at | -2.49425 | AK120662 // Oryza sativa Japonica Group cDNA clone:J013158K14, full insert seque | 0.00270629 | 0.04402 |
| Os.50578.1.S1_at | -2.49222 | AK070683 // Oryza sativa Japonica Group cDNA clone:J023062D08, full insert seque | 0.00800527 | 0.05484 |
| Os.35335.1.A1_s_at | -2.48905 | AB188836 // Oryza sativa Japonica Group OsSK3 mRNA for shikimate kinase 3, compl | 0.00144824 | 0.03228 |
| Os.5809.1.S1_s_at | -2.48834 | AK070289 // Oryza sativa Japonica Group cDNA clone:J023050F01, full insert seque | 0.00213759 | 0.05629 |
| OsAffx.16386.1.S1_at | -2.48019 | CT837550 // Oryza sativa (indica cultivar-group) cDNA clone:OSIGCSN082O06, full | 0.00172297 | 0.03977 |
| Os.47879.1.A1_at | -2.4777 | AK111367 // Oryza sativa Japonica Group cDNA clone:002-182-A01, full insert sequ | 0.000740835 | 0.04997 |
| Os.7270.1.S1_x_at | -2.47589 | AK066385 // Oryza sativa Japonica Group cDNA clone:J013067H21, full insert seque | 0.031957 | 0.1668 |
| Os.38320.2.S1_at | -2.46656 | AK066256 // Oryza sativa Japonica Group cDNA clone:J013057C10, full insert seque | 0.00658182 | 0.05856 |
| Os.46382.4.S1_at | -2.46225 | BGIOSSBCE030337.1 // --- // bgi_ris // 10 // --- /// BGIOSSFCE030771.1 // --- // | 0.00183711 | 0.05085 |
| Os.52018.1.S1_at | -2.44721 | AK064542 // Oryza sativa Japonica Group cDNA clone:002-112-A08, full insert sequ | 0.00215928 | 0.0357 |
| Os.51917.1.S1_at | -2.4469 | LOC_Os08g15920.1 // expressed protein // rgap // 11 // --- /// NM_001067922 // O | 0.00764404 | 0.06093 |
| Os.46127.1.S1_x_at | -2.4365 | BGIOSSBCE030322.1 // --- // bgi_ris // 11 // --- /// LOC_Os10g35260.1 // Rf1, mi | 0.00138727 | 0.03427 |
| Os.49126.1.S1_at | -2.43128 | AK069411 // Oryza sativa Japonica Group cDNA clone:J023013G15, full insert seque | 0.00545798 | 0.0452 |
| Os.27441.1.S1_at | -2.42037 | AK288620 // Oryza sativa Japonica Group cDNA, clone: J090054E12, full insert seq | 0.00023024 | 0.03481 |
| Os.9756.1.S1_a_at | -2.42013 | AK099305 // Oryza sativa Japonica Group cDNA clone:J023140I16, full insert seque | 0.00100976 | 0.04404 |
| Os.52428.1.S1_x_at | -2.41588 | AK067059 // Oryza sativa Japonica Group cDNA clone:J013090O06, full insert seque | 0.00314487 | 0.03825 |
| Os.14285.2.S1_at | -2.41537 | AK063201 // Oryza sativa Japonica Group cDNA clone:001-112-C08, full insert sequ | 0.00267414 | 0.03614 |
| Os.51069.1.S1_s_at | -2.41272 | LOC_Os06g19550.1 // oxidoreductase, short chain dehydrogenase/reductase family, | 0.00793777 | 0.0791 |
| Os.44984.2.S1_at | -2.41032 | BGIOSSBCE012242.1 // --- // bgi_ris // 9 // --- | 0.000457603 | 0.02124 |
| Os.33602.1.S2_at | -2.40171 | AK099933 // Oryza sativa Japonica Group cDNA clone:J013119E02, full insert seque | 0.00626406 | 0.07089 |
| Os.49351.2.S1_x_at | -2.39913 | AK071696 // Oryza sativa Japonica Group cDNA clone:J023109P16, full insert seque | 0.00597113 | 0.04841 |
| Os.13996.1.S1_at | -2.3961 | LOC_Os07g40860.1 // retrotransposon protein, putative, unclassified, expressed / | 0.00369666 | 0.08057 |
| OsAffx.23824.1.S1_x_at | -2.38782 | BGIOSSBCE003517.1 // --- // bgi_ris // 0 // chr1:33237790-33242310(+) /// LOC_Os | 0.012046 | 0.05591 |
| Os.27107.1.S1_at | -2.38243 | AK065556 // Oryza sativa Japonica Group cDNA clone:J013028C04, full insert seque | 0.00510037 | 0.04353 |
| Os.35012.1.S1_at | -2.37719 | AK069284 // Oryza sativa Japonica Group cDNA clone:J023010F15, full insert seque | 0.00555477 | 0.04849 |
| Os.5061.1.S1_s_at | -2.3737 | LOC_Os04g09720.2 // OsSCP22 - Putative Serine Carboxypeptidase homologue, expres | 0.00422361 | 0.06096 |
| Os.46862.1.S1_at | -2.37103 | AK106927 // Oryza sativa Japonica Group cDNA clone:002-119-B11, full insert sequ | 0.00284495 | 0.04018 |
| Os.46610.1.S1_x_at | -2.36602 | AK241879 // Oryza sativa Japonica Group cDNA, clone: J065218I11, full insert seq | 0.0035898 | 0.06107 |
| Os.54918.1.S1_at | -2.35607 | NM_001070604 // Oryza sativa (japonica cultivar-group) Os10g0123200 (Os10g012320 | 0.0122259 | 0.0929 |
| Os.38190.2.A1_at | -2.35186 | CB636550 // /DB_XREF=OSJNEa02C20.r /CLONE=OSJNEa02C20 // gb // --- // --- | 0.000525832 | 0.06003 |
| Os.27137.1.S1_at | -2.35094 | AK067144 // Oryza sativa Japonica Group cDNA clone:J013096F13, full insert seque | 0.00257809 | 0.03614 |
| OsAffx.5660.1.S1_at | -2.34856 | BGIOSSFCE024527.1 // --- // bgi_ris // 11 // --- /// LOC_Os07g47490.1 // OsGH3.1 | 0.00814802 | 0.0863 |
| Os.27790.2.A1_x_at | -2.34679 | AK119263 // Oryza sativa Japonica Group cDNA clone:001-130-A03, full insert sequ | 0.000390326 | 0.0448 |
| Os.46924.1.S1_at | -2.34194 | LOC_Os07g40690.1 // NAD dependent epimerase/dehydratase family protein, putative | 0.00230286 | 0.05715 |
| Os.32682.1.S1_at | -2.34152 | LOC_Os07g02960.1 // expressed protein // rgap // 11 // --- /// LOC_Os07g02960.2 | 0.00832202 | 0.05118 |
| Os.57129.1.S1_at | -2.33919 | AK110969 // Oryza sativa Japonica Group cDNA clone:002-174-B09, full insert sequ | 0.00118361 | 0.03228 |
| Os.25222.1.S1_at | -2.33799 | AK068168 // Oryza sativa Japonica Group cDNA clone:J013129H24, full insert seque | 0.00292253 | 0.05715 |
| Os.56140.1.S1_x_at | -2.3377 | NM_001057470 // Oryza sativa (japonica cultivar-group) Os03g0686700 (Os03g068670 | 0.0176213 | 0.06799 |
| Os.14006.1.S1_at | -2.3336 | LOC_Os08g09230.1 // starch synthase III, putative, expressed // rgap // 10 // -- | 0.00230042 | 0.06077 |
| Os.4530.1.S1_at | -2.33003 | AK099481 // Oryza sativa Japonica Group cDNA clone:J013025G09, full insert seque | 0.0104124 | 0.05232 |
| OsAffx.15267.1.S1_at | -2.32807 | BGIOSSBCE018997.1 // --- // bgi_ris // 9 // --- | 0.00400933 | 0.03931 |
| OsAffx.30971.1.S1_at | -2.32622 | AK289085 // Oryza sativa Japonica Group cDNA, clone: J090095J19, full insert seq | 0.00292329 | 0.03809 |
| Os.46873.1.S1_a_at | -2.32224 | AK069758 // Oryza sativa Japonica Group cDNA clone:J023033H12, full insert seque | 0.0012334 | 0.02741 |
| Os.25305.1.A1_s_at | -2.31976 | AK066577 // Oryza sativa Japonica Group cDNA clone:J013069I21, full insert seque | 0.0230576 | 0.0719 |
| Os.7526.1.S1_at | -2.31699 | NM_001074097 // Oryza sativa (japonica cultivar-group) Os11g0235400 (Os11g023540 | 0.00574823 | 0.05623 |
| Os.50721.1.S1_at | -2.31661 | AJ532520 // Oryza sativa Z274 snoRNA. // gb // 11 // --- | 0.0441632 | 0.1002 |
| Os.7743.2.S2_at | -2.31535 | AK106768 // Oryza sativa Japonica Group cDNA clone:002-115-F05, full insert sequ | 0.000638393 | 0.02124 |
| Os.57351.1.S1_at | -2.31002 | AK111356 // Oryza sativa Japonica Group cDNA clone:002-181-H01, full insert sequ | 0.0382044 | 0.09112 |
| Os.2353.2.S1_at | -2.30979 | AF193802 // Oryza sativa zinc finger transcription factor WRKY1 mRNA, complete c | 0.00630102 | 0.0612 |
| Os.4708.1.S1_at | -2.30756 | AK241797 // Oryza sativa Japonica Group cDNA, clone: J065208F10, full insert seq | 0.00874367 | 0.05031 |
| Os.39993.1.S1_at | -2.30427 | LOC_Os03g60639.1 // expressed protein // rgap // 11 // --- | 0.00212812 | 0.03449 |
| OsAffx.14635.1.S1_x_at | -2.30025 | LOC_Os05g11260.2 // expressed protein // rgap // 11 // --- | 0.000910804 | 0.03629 |
| Os.22678.1.A1_s_at | -2.29816 | AK068298 // Oryza sativa Japonica Group cDNA clone:J013146L01, full insert seque | 0.00274707 | 0.03562 |
| Os.25311.1.A1_at | -2.29065 | AK108784 // Oryza sativa Japonica Group cDNA clone:002-151-A10, full insert sequ | 0.0264565 | 0.1199 |
| Os.26838.1.S1_at | -2.28933 | CT828814 // Oryza sativa (indica cultivar-group) cDNA clone:OSIGCRA222E02, full | 0.00528782 | 0.07037 |
| Os.49409.1.S1_at | -2.28276 | AK100360 // Oryza sativa Japonica Group cDNA clone:J023083L23, full insert seque | 0.00117346 | 0.02961 |
| OsAffx.13735.1.S1_at | -2.27956 | BGIOSSFCE013425.1 // --- // bgi_ris // 11 // --- /// LOC_Os04g09720.1 // OsSCP22 | 0.016175 | 0.08923 |
| Os.53535.1.S1_at | -2.27162 | AK073173 // Oryza sativa Japonica Group cDNA clone:J033023N06, full insert seque | 0.00191913 | 0.03284 |
| Os.5678.1.S1_x_at | -2.27125 | AK064884 // Oryza sativa Japonica Group cDNA clone:J013000K07, full insert seque | 0.00136457 | 0.03465 |
| Os.35778.1.S1_at | -2.27004 | LOC_Os01g56380.1 // decarboxylase, putative, expressed // rgap // 11 // --- /// | 0.00641391 | 0.04542 |
| Os.46826.1.S1_at | -2.26774 | LOC_Os10g35680.1 // acetyltransferase, GNAT family, putative, expressed // rgap | 0.000524534 | 0.05588 |
| Os.31681.1.S1_at | -2.26578 | LOC_Os01g05480.1 // expressed protein // rgap // 11 // --- /// LOC_Os01g05480.2 | 0.00282621 | 0.03825 |
| Os.56110.1.S1_at | -2.26402 | AK109271 // Oryza sativa Japonica Group cDNA clone:002-188-A07, full insert sequ | 0.00590071 | 0.04679 |
| Os.49629.1.S1_at | -2.26085 | AK069419 // Oryza sativa Japonica Group cDNA clone:J023019B04, full insert seque | 0.0027433 | 0.03562 |
| Os.17844.1.S1_at | -2.25583 | AK100428 // Oryza sativa Japonica Group cDNA clone:J023088D18, full insert seque | 0.000852044 | 0.02369 |
| Os.50340.1.S1_at | -2.25332 | AK120796 // Oryza sativa Japonica Group cDNA clone:J023012D20, full insert seque | 0.004334 | 0.06334 |
| Os.54088.1.S1_at | -2.25318 | LOC_Os12g10810.1 // expressed protein // rgap // 11 // --- /// NM_001072934 // O | 0.000585838 | 0.0357 |
| Os.12081.2.S1_s_at | -2.25021 | AK111632 // Oryza sativa Japonica Group cDNA clone:J013116G01, full insert seque | 0.000704692 | 0.02298 |
| Os.26877.1.A1_at | -2.24917 | LOC_Os10g38930.1 // shikimate/quinate 5-dehydrogenase, putative, expressed // rg | 0.00224222 | 0.06277 |
| OsAffx.5461.1.S1_at | -2.24754 | LOC_Os07g27390.1 // retrotransposon protein, putative, unclassified, expressed / | 0.00119098 | 0.03481 |
| Os.57506.1.S1_at | -2.24399 | AK287607 // Oryza sativa Japonica Group cDNA, clone: J065057L02, full insert seq | 0.0481004 | 0.1069 |
| Os.49942.1.S1_at | -2.24393 | LOC_Os04g41000.1 // expressed protein // rgap // 11 // --- /// NM_001059677 // O | 0.00870206 | 0.06189 |
| Os.57121.1.S1_at | -2.24172 | AK110958 // Oryza sativa Japonica Group cDNA clone:002-174-A09, full insert sequ | 0.00946083 | 0.06219 |
| Os.50612.1.A1_at | -2.24115 | AK122092 // Oryza sativa Japonica Group cDNA clone:J033124E22, full insert seque | 0.00252802 | 0.04016 |
| Os.27541.1.A1_at | -2.23656 | CB635942 // --- // gb // --- // --- | 0.03863 | 0.09474 |
| Os.10497.1.S1_s_at | -2.23598 | BGIOSSBCE022732.1 // --- // bgi_ris // 11 // --- /// LOC_Os07g23640.1 // retrotr | 0.018486 | 0.07375 |
| OsAffx.2125.1.S1_at | -2.23582 | BGIOSSFCE001860.1 // --- // bgi_ris // 0 // chr1:17225614-17227652(-) /// LOC_Os | 0.0108866 | 0.05329 |
| Os.52587.1.S1_at | -2.23326 | AK068189 // Oryza sativa Japonica Group cDNA clone:J013135F21, full insert seque | 0.00113503 | 0.0287 |
| Os.50601.1.S1_x_at | -2.2332 | AK065412 // Oryza sativa Japonica Group cDNA clone:J013021C10, full insert seque | 0.0121187 | 0.1052 |
| Os.54882.1.S1_at | -2.2327 | NM_001052472 // Oryza sativa (japonica cultivar-group) Os02g0155000 (Os02g015500 | 0.0000077 | 0.02059 |
| Os.8276.1.S1_at | -2.23154 | AK108963 // Oryza sativa Japonica Group cDNA clone:002-153-D05, full insert sequ | 0.010289 | 0.0697 |
| Os.48491.1.S1_at | -2.22092 | AK106259 // Oryza sativa Japonica Group cDNA clone:002-100-F05, full insert sequ | 0.0022853 | 0.03449 |
| Os.25120.1.S1_at | -2.21978 | AK243497 // Oryza sativa Japonica Group cDNA, clone: J100074E09, full insert seq | 0.000953409 | 0.02828 |
| Os.50443.1.S1_at | -2.21864 | AK121210 // Oryza sativa Japonica Group cDNA clone:J023089C08, full insert seque | 0.00901783 | 0.05199 |
| OsAffx.29875.1.S1_s_at | -2.21446 | LOC_Os09g16520.1 // cytochrome b5-like Heme/Steroid binding domain containing pr | 0.000144533 | 0.01655 |
| OsAffx.7458.1.S1_x_at | -2.21331 | BGIOSSBCE033102.1 // --- // bgi_ris // 11 // --- /// LOC_Os12g02020.1 // conserv | 0.0105846 | 0.05967 |
| Os.29008.1.S1_at | -2.21257 | LOC_Os01g08370.1 // symbiosis-related disease resistance protein, putative, expr | 0.000454912 | 0.04879 |
| Os.53931.1.S1_at | -2.20984 | CT831117 // Oryza sativa (indica cultivar-group) cDNA clone:OSIGCSN074E03, full | 0.00785723 | 0.0694 |
| Os.31837.2.S1_x_at | -2.20931 | AK106659 // Oryza sativa Japonica Group cDNA clone:002-113-G10, full insert sequ | 0.00406391 | 0.03977 |
| Os.50364.1.S1_at | -2.20925 | AK120886 // Oryza sativa Japonica Group cDNA clone:J023028P22, full insert seque | 0.00275187 | 0.1061 |
| Os.27237.1.A1_a_at | -2.20855 | AK070073 // Oryza sativa Japonica Group cDNA clone:J023043F15, full insert seque | 0.0132399 | 0.1012 |
| Os.54548.1.S1_at | -2.20832 | LOC_Os02g54560.1 // SCP-like extracellular protein, expressed // rgap // 11 // - | 0.00578294 | 0.0995 |
| Os.9978.1.S1_at | -2.20697 | AK121200 // Oryza sativa Japonica Group cDNA clone:J023087I02, full insert seque | 0.00285663 | 0.05293 |
| Os.52511.1.S1_at | -2.20681 | AK067592 // Oryza sativa Japonica Group cDNA clone:J013110N20, full insert seque | 0.00932256 | 0.05085 |
| Os.166.2.S1_at | -2.20622 | AK107838 // Oryza sativa Japonica Group cDNA clone:002-133-H07, full insert sequ | 0.000534387 | 0.02369 |
| Os.16006.1.S1_s_at | -2.20557 | AK240782 // Oryza sativa Japonica Group cDNA, clone: J053076M17, full insert seq | 0.0031994 | 0.03825 |
| Os.49440.1.S1_at | -2.20443 | AK101626 // Oryza sativa Japonica Group cDNA clone:J033052M16, full insert seque | 0.00579928 | 0.04467 |
| Os.54840.1.S1_at | -2.20378 | LOC_Os08g09730.1 // OsFBX273 - F-box domain containing protein, expressed // rga | 0.0183322 | 0.06634 |
| Os.26955.1.S1_at | -2.202 | AK101328 // Oryza sativa Japonica Group cDNA clone:J033034G12, full insert seque | 0.013419 | 0.06867 |
| Os.26561.1.S1_at | -2.20098 | AK100400 // Oryza sativa Japonica Group cDNA clone:J023086N14, full insert seque | 0.00073247 | 0.09358 |
| Os.51245.1.S1_at | -2.20085 | LOC_Os02g32450.1 // expressed protein // rgap // 11 // --- /// NM_001053526 // O | 0.000677794 | 0.04698 |
| Os.26561.1.S2_at | -2.1965 | AK100400 // Oryza sativa Japonica Group cDNA clone:J023086N14, full insert seque | 0.000428979 | 0.07304 |
| OsAffx.22461.1.S1_at | -2.19627 | LOC_Os01g54150.1 // retrotransposon protein, putative, unclassified // rgap // 0 | 0.00128518 | 0.03628 |
| Os.23712.1.S1_at | -2.19616 | LOC_Os08g17784.1 // purple acid phosphatase, putative, expressed // rgap // 11 / | 0.000518149 | 0.1421 |
| OsAffx.24769.1.S1_s_at | -2.1952 | AK121386 // Oryza sativa Japonica Group cDNA clone:J023127K17, full insert seque | 0.0252244 | 0.2182 |
| Os.49058.1.A1_at | -2.1938 | AU031529 // /DB_XREF=AU031529 /CLONE=E61792_2Z // gb // --- // --- /// CR288652 | 0.00549792 | 0.04402 |
| Os.7996.1.S1_at | -2.1912 | AK070691 // Oryza sativa Japonica Group cDNA clone:J023058J05, full insert seque | 0.00311907 | 0.04402 |
| OsAffx.7664.1.S1_s_at | -2.19098 | AK067455 // Oryza sativa Japonica Group cDNA clone:J013105I07, full insert seque | 0.00500207 | 0.04841 |
| Os.54058.1.S1_at | -2.18974 | AK101620 // Oryza sativa Japonica Group cDNA clone:J033052J02, full insert seque | 0.00312675 | 0.04684 |
| Os.51986.1.S1_at | -2.18796 | AK064471 // Oryza sativa Japonica Group cDNA clone:002-110-H05, full insert sequ | 0.0167206 | 0.06301 |
| Os.22578.1.A1_at | -2.1864 | LOC_Os07g10130.1 // squalene synthetase, putative, expressed // rgap // 9 // --- | 0.0140916 | 0.05914 |
| Os.34424.1.S1_at | -2.18579 | AK071457 // Oryza sativa Japonica Group cDNA clone:J023090J21, full insert seque | 0.00122099 | 0.02768 |
| Os.6094.2.A1_s_at | -2.18391 | LOC_Os11g38330.1 // ZOS11-06 - C2H2 zinc finger protein, expressed // rgap // 11 | 0.000492034 | 0.02905 |
| Os.45902.1.A1_at | -2.18366 | BGIOSSFCE016898.1 // --- // bgi_ris // 0 // chr12:9977959-10011703(-) /// CT8280 | 0.0232597 | 0.07227 |
| Os.49948.1.S1_at | -2.18309 | AK119419 // Oryza sativa Japonica Group cDNA clone:001-132-G10, full insert sequ | 0.0197128 | 0.08741 |
| Os.52219.1.A1_at | -2.18287 | AK065704 // Oryza sativa Japonica Group cDNA clone:J013037C19, full insert seque | 0.0016543 | 0.0329 |
| OsAffx.31243.1.S1_at | -2.18174 | AK243369 // Oryza sativa Japonica Group cDNA, clone: J100062D04, full insert seq | 0.00613173 | 0.04741 |
| Os.9394.1.S1_at | -2.1804 | AK065231 // Oryza sativa Japonica Group cDNA clone:J013002H11, full insert seque | 0.00091516 | 0.06892 |
| Os.6345.1.S1_at | -2.17947 | BGIOSSBCE020579.1 // --- // bgi_ris // 9 // --- /// BGIOSSFCE020842.1 // --- // | 0.000816939 | 0.1948 |
| OsAffx.7824.1.S1_s_at | -2.1763 | AK064852 // Oryza sativa Japonica Group cDNA clone:J013000H05, full insert seque | 0.00216198 | 0.03442 |
| Os.7253.1.S1_at | -2.17516 | LOC_Os12g28770.1 // POEI19 - Pollen Ole e I allergen and extensin family protein | 0.00230871 | 0.0526 |
| Os.53740.1.S1_at | -2.17471 | BGIOSSFSE035710.1 // --- // bgi_ris // 11 // --- /// LOC_Os11g09850.2 // express | 0.00143292 | 0.03629 |
| Os.11583.1.S1_at | -2.17402 | AK068792 // Oryza sativa Japonica Group cDNA clone:J013163E23, full insert seque | 0.00275643 | 0.04257 |
| Os.20239.1.S1_at | -2.17216 | LOC_Os08g42100.1 // ACT domain containing protein, expressed // rgap // 11 // -- | 0.0115944 | 0.05856 |
| Os.18262.1.S1_at | -2.17055 | AK100899 // Oryza sativa Japonica Group cDNA clone:J023131G20, full insert seque | 0.00317577 | 0.05979 |
| Os.43636.1.S1_at | -2.16964 | LOC_Os01g25060.1 // transposon protein, putative, CACTA, En/Spm sub-class // rga | 0.000978499 | 0.0287 |
| Os.17844.1.S1_x_at | -2.16944 | AK100428 // Oryza sativa Japonica Group cDNA clone:J023088D18, full insert seque | 0.000519589 | 0.02124 |
| Os.56260.1.S1_x_at | -2.16774 | LOC_Os08g09710.1 // OsFBX271 - F-box domain containing protein, expressed // rga | 0.00351307 | 0.05085 |
| Os.53542.1.S1_at | -2.16414 | CT832028 // Oryza sativa (indica cultivar-group) cDNA clone:OSIGCPI020I24, full | 0.0163173 | 0.06761 |
| OsAffx.29837.1.S1_s_at | -2.16317 | LOC_Os09g13820.1 // pollen signalling protein with adenylyl cyclase activity, pu | 0.000681851 | 0.03449 |
| Os.15012.1.S2_a_at | -2.16223 | AK102573 // Oryza sativa Japonica Group cDNA clone:J033097M22, full insert seque | 0.000904049 | 0.02828 |
| Os.51189.1.S2_at | -2.16091 | AK061458 // Oryza sativa Japonica Group cDNA clone:006-307-H09, full insert sequ | 0.0477902 | 0.217 |
| Os.9552.1.S1_s_at | -2.16091 | LOC_Os12g44040.4 // transposon protein, putative, unclassified, expressed // rga | 0.00877589 | 0.05118 |
| Os.6812.1.S1_at | -2.15971 | LOC_Os10g21670.1 // dehydration stress-induced protein, putative, expressed // r | 0.0394146 | 0.1468 |
| OsAffx.31739.1.S1_at | -2.15942 | BGIOSSFCE034321.1 // --- // bgi_ris // 11 // --- /// LOC_Os12g14310.1 // hypothe | 0.012112 | 0.05845 |
| Os.47784.1.S1_at | -2.15779 | LOC_Os06g45340.1 // peptidyl-prolyl cis-trans isomerase, FKBP-type, putative, ex | 0.000872026 | 0.0239 |
| OsAffx.15781.4.S1_x_at | -2.15724 | LOC_Os04g21740.1 // conserved hypothetical protein // rgap // 11 // --- /// LOC_ | 0.0294021 | 0.08021 |
| Os.53725.1.S1_at | -2.15606 | AK099535 // Oryza sativa Japonica Group cDNA clone:J013031N15, full insert seque | 0.00789261 | 0.05395 |
| Os.50340.2.S1_x_at | -2.15508 | AK059623 // Oryza sativa Japonica Group cDNA clone:001-030-H05, full insert sequ | 0.00860475 | 0.08021 |
| Os.50290.1.S1_at | -2.15503 | AK065834 // Oryza sativa Japonica Group cDNA clone:J013036J09, full insert seque | 0.00586648 | 0.06747 |
| Os.57024.1.S1_at | -2.15363 | AK110761 // Oryza sativa Japonica Group cDNA clone:002-170-H07, full insert sequ | 0.0173279 | 0.06415 |
| Os.46507.1.S1_at | -2.1516 | AK070040 // Oryza sativa Japonica Group cDNA clone:J023036G13, full insert seque | 0.040471 | 0.1375 |
| Os.54254.1.S1_x_at | -2.14936 | AK066199 // Oryza sativa Japonica Group cDNA clone:J013052B17, full insert seque | 0.0054864 | 0.04631 |
| Os.51681.1.S1_at | -2.14782 | LOC_Os06g38430.1 // expressed protein // rgap // 11 // --- /// NM_001064476 // O | 0.0010469 | 0.03449 |
| Os.11563.1.S1_at | -2.14706 | LOC_Os11g43980.1 // peroxidase precursor, putative, expressed // rgap // 11 // - | 0.0412191 | 0.2614 |
| Os.50426.2.S1_x_at | -2.14636 | AK101623 // Oryza sativa Japonica Group cDNA clone:J033052L15, full insert seque | 0.00496653 | 0.08759 |
| Os.51189.1.S1_at | -2.14597 | AK100583 // Oryza sativa Japonica Group cDNA clone:J023105O13, full insert seque | 0.00414883 | 0.03937 |
| Os.46710.2.S1_at | -2.14551 | AK122036 // Oryza sativa Japonica Group cDNA clone:J033112O18, full insert seque | 0.00623454 | 0.04505 |
| Os.6732.1.S1_a_at | -2.14532 | AK071497 // Oryza sativa Japonica Group cDNA clone:J023098M19, full insert seque | 0.00125466 | 0.05408 |
| Os.18255.1.S1_at | -2.14513 | AK072230 // Oryza sativa Japonica Group cDNA clone:J023002D21, full insert seque | 0.00385723 | 0.03863 |
| Os.50262.1.S1_at | -2.14047 | AK120412 // Oryza sativa Japonica Group cDNA clone:J013092B14, full insert seque | 0.0152485 | 0.0719 |
| Os.18572.1.S1_at | -2.13893 | AK069804 // Oryza sativa Japonica Group cDNA clone:J023026J07, full insert seque | 0.00482025 | 0.09575 |
| Os.51964.1.S1_at | -2.13754 | AK064422 // Oryza sativa Japonica Group cDNA clone:002-110-B12, full insert sequ | 0.00258379 | 0.03481 |
| Os.55393.2.S1_at | -2.13657 | AK107998 // Oryza sativa Japonica Group cDNA clone:002-135-G03, full insert sequ | 0.00499546 | 0.04513 |
| Os.11454.2.S1_at | -2.13656 | AK106698 // Oryza sativa Japonica Group cDNA clone:002-114-D11, full insert sequ | 0.00209099 | 0.07892 |
| Os.49625.1.S1_at | -2.13214 | AK061480 // Oryza sativa Japonica Group cDNA clone:006-308-G10, full insert sequ | 0.00180713 | 0.03745 |
| Os.50578.1.S1_x_at | -2.12553 | AK070683 // Oryza sativa Japonica Group cDNA clone:J023062D08, full insert seque | 0.000488573 | 0.02567 |
| Os.5140.1.S1_at | -2.1223 | LOC_Os10g35130.1 // expressed protein // rgap // 11 // --- /// NM_001071475 // O | 0.0036454 | 0.03937 |
| OsAffx.31528.1.S1_x_at | -2.11881 | LOC_Os11g25530.1 // hypothetical protein // rgap // 11 // --- /// LOC_Os11g47750 | 0.0145917 | 0.06048 |
| Os.19018.2.S1_at | -2.11826 | AK111577 // Oryza sativa Japonica Group cDNA clone:J013075F18, full insert seque | 0.00216169 | 0.03449 |
| Os.11604.2.S1_at | -2.11656 | LOC_Os10g42190.2 // leucine rich repeat containing protein, expressed // rgap // | 0.0192208 | 0.08539 |
| OsAffx.8749.1.S1_at | -2.11373 | BGIOSSBCE021608.1 // --- // bgi_ris // 0 // chr7:181769-182171(-) /// LOC_Os07g0 | 0.0113971 | 0.08879 |
| Os.25340.1.A1_at | -2.11219 | AK072856 // Oryza sativa Japonica Group cDNA clone:J023135F23, full insert seque | 0.0124936 | 0.06687 |
| Os.50496.1.S1_at | -2.11116 | LOC_Os04g52950.1 // nitrate-induced NOI protein, expressed // rgap // 11 // --- | 0.0202619 | 0.0691 |
| Os.52179.1.S1_at | -2.11077 | AK065469 // Oryza sativa Japonica Group cDNA clone:J013009B01, full insert seque | 0.000869013 | 0.02961 |
| Os.9321.1.S1_a_at | -2.1099 | AK068840 // Oryza sativa Japonica Group cDNA clone:J013160B01, full insert seque | 0.00751179 | 0.05868 |
| Os.50614.1.S1_at | -2.10963 | LOC_Os08g27580.2 // expressed protein // rgap // 11 // --- /// LOC_Os08g27580.4 | 0.0137876 | 0.06879 |
| Os.36118.1.S1_at | -2.10942 | AK064449 // Oryza sativa Japonica Group cDNA clone:002-110-F04, full insert sequ | 0.0210062 | 0.0722 |
| Os.47348.1.A1_at | -2.10911 | AK068893 // Oryza sativa Japonica Group cDNA clone:J023001G24, full insert seque | 0.0128948 | 0.06619 |
| OsAffx.15281.1.S1_at | -2.10856 | AK061306 // Oryza sativa Japonica Group cDNA clone:006-301-H08, full insert sequ | 0.0136544 | 0.06761 |
| OsAffx.3349.1.S1_at | -2.1085 | AK242576 // Oryza sativa Japonica Group cDNA, clone: J090009A15, full insert seq | 0.000589269 | 0.03449 |
| Os.53079.1.S1_at | -2.10796 | AK070730 // Oryza sativa Japonica Group cDNA clone:J023063K10, full insert seque | 0.0120498 | 0.06981 |
| Os.7097.2.S1_a_at | -2.10702 | AK072228 // Oryza sativa Japonica Group cDNA clone:J013169I21, full insert seque | 0.0259326 | 0.07878 |
| OsAffx.16407.2.S1_x_at | -2.10261 | LOC_Os02g27830.1 // conserved hypothetical protein // rgap // 11 // --- /// Os02 | 0.00847831 | 0.04951 |
| Os.15012.1.S1_a_at | -2.10173 | AK102573 // Oryza sativa Japonica Group cDNA clone:J033097M22, full insert seque | 0.0056087 | 0.05927 |
| OsAffx.26722.2.S1_s_at | -2.10097 | AK288062 // Oryza sativa Japonica Group cDNA, clone: J075154N16, full insert seq | 0.0160174 | 0.1192 |
| Os.23660.1.S1_at | -2.10012 | AK121072 // Oryza sativa Japonica Group cDNA clone:J023061N13, full insert seque | 0.00113944 | 0.03393 |
| Os.51928.1.S1_at | -2.09961 | AK064299 // Oryza sativa Japonica Group cDNA clone:002-107-A07, full insert sequ | 0.0106572 | 0.08021 |
| Os.38301.2.A1_at | -2.09953 | AK068507 // Oryza sativa Japonica Group cDNA clone:J013154C12, full insert seque | 0.00190858 | 0.03825 |
| Os.50821.1.S1_at | -2.09894 | AK058781 // Oryza sativa Japonica Group cDNA clone:001-002-C10, full insert sequ | 0.013151 | 0.06841 |
| OsAffx.3291.1.S1_at | -2.09839 | LOC_Os03g21870.1 // DUF623 domain containing protein, expressed // rgap // 11 // | 0.00427966 | 0.04619 |
| Os.23796.1.S1_at | -2.09719 | AK105562 // Oryza sativa Japonica Group cDNA clone:001-128-B10, full insert sequ | 0.00291699 | 0.03931 |
| OsAffx.27601.1.S1_at | -2.09619 | LOC_Os06g13100.3 // CREG1 precursor, putative, expressed // rgap // 11 // --- | 0.0334222 | 0.08544 |
| Os.50260.2.A1_x_at | -2.09614 | AK120396 // Oryza sativa Japonica Group cDNA clone:J013075J05, full insert seque | 0.0267402 | 0.1117 |
| Os.48846.1.S1_at | -2.09599 | AK059073 // Oryza sativa Japonica Group cDNA clone:001-021-H09, full insert sequ | 0.0467312 | 0.1411 |
| Os.12951.1.S1_at | -2.09487 | LOC_Os10g41590.1 // zinc finger, C3HC4 type domain containing protein, expressed | 0.00739911 | 0.05283 |
| Os.7262.1.S1_at | -2.09375 | AK066187 // Oryza sativa Japonica Group cDNA clone:J013058P10, full insert seque | 0.000895265 | 0.02774 |
| Os.10502.1.S1_at | -2.0923 | AK071662 // Oryza sativa Japonica Group cDNA clone:J023106O11, full insert seque | 0.00696536 | 0.04681 |
| Os.56260.1.S1_at | -2.09228 | LOC_Os08g09710.1 // OsFBX271 - F-box domain containing protein, expressed // rga | 0.0018445 | 0.05118 |
| Os.50228.1.S1_at | -2.09202 | AK120208 // Oryza sativa Japonica Group cDNA clone:J013038K24, full insert seque | 0.0354861 | 0.09007 |
| Os.28411.1.S1_at | -2.0916 | AK064853 // Oryza sativa Japonica Group cDNA clone:J013000H06, full insert seque | 0.00031986 | 0.04018 |
| OsAffx.31428.1.S1_s_at | -2.08826 | AK102163 // Oryza sativa Japonica Group cDNA clone:J033086H01, full insert seque | 0.0251679 | 0.07902 |
| Os.23521.1.A1_at | -2.08625 | CA759870 // /DB_XREF=BR060005B10D02.ab1 /CLONE=BR060005B10D02.ab1 // gb // --- / | 0.0366747 | 0.09404 |
| Os.6325.2.A1_x_at | -2.08392 | LOC_Os01g39770.1 // calcineurin B, putative, expressed // rgap // 11 // --- /// | 0.000213458 | 0.03809 |
| Os.50516.1.S1_at | -2.08346 | AK121572 // Oryza sativa Japonica Group cDNA clone:J033035N11, full insert seque | 0.000798129 | 0.03943 |
| Os.17347.1.S1_at | -2.08302 | AK105854 // Oryza sativa Japonica Group cDNA clone:001-203-H03, full insert sequ | 0.0209833 | 0.1042 |
| Os.27334.1.S1_at | -2.08233 | AK102828 // Oryza sativa Japonica Group cDNA clone:J033109D12, full insert seque | 0.00330157 | 0.06044 |
| Os.16645.1.A1_at | -2.08227 | LOC_Os12g10640.1 // uncharacterized protein ycf45, putative, expressed // rgap / | 0.00675395 | 0.06277 |
| Os.33834.1.S1_at | -2.08213 | AK073863 // Oryza sativa Japonica Group cDNA clone:J033071F17, full insert seque | 0.0301698 | 0.08613 |
| Os.50886.1.S1_at | -2.08201 | CT830507 // Oryza sativa (indica cultivar-group) cDNA clone:OSIGCPI001B07, full | 0.00629233 | 0.05085 |
| OsAffx.15177.1.S1_at | -2.0814 | BGIOSSFCE018873.1 // --- // bgi_ris // 11 // --- /// LOC_Os05g50260.1 // polygal | 0.00671291 | 0.04967 |
| Os.22884.1.A1_at | -2.07829 | AK242751 // Oryza sativa Japonica Group cDNA, clone: J090050I20, full insert seq | 0.0059754 | 0.0698 |
| Os.49802.1.S1_at | -2.07579 | LOC_Os07g23030.1 // retrotransposon protein, putative, Ty3-gypsy subclass, expre | 0.000456163 | 0.02154 |
| Os.51232.1.S1_at | -2.07487 | AK062294 // Oryza sativa Japonica Group cDNA clone:001-100-F02, full insert sequ | 0.0294024 | 0.08952 |
| Os.55363.1.S1_at | -2.07486 | LOC_Os07g40250.1 // sex determination protein tasselseed-2, putative, expressed | 0.000243693 | 0.04361 |
| Os.27645.1.S1_x_at | -2.0742 | AK121726 // Oryza sativa Japonica Group cDNA clone:J033082C04, full insert seque | 0.00128144 | 0.0297 |
| Os.17180.1.S1_at | -2.07294 | CT834446 // Oryza sativa (indica cultivar-group) cDNA clone:OSIGCPI223D19, full | 0.0127568 | 0.2608 |
| Os.50627.1.S1_at | -2.07222 | LOC_Os04g58050.1 // expressed protein // rgap // 11 // --- /// NM_001060795 // O | 0.00114565 | 0.03228 |
| Os.23613.1.S1_at | -2.0696 | LOC_Os06g07020.1 // ZOS6-01 - C2H2 zinc finger protein, expressed // rgap // 10 | 0.0490067 | 0.1074 |
| OsAffx.15781.5.S1_x_at | -2.06917 | LOC_Os04g12190.1 // hAT dimerisation domain-containing protein, putative // rgap | 0.00110725 | 0.02774 |
| Os.17405.1.S1_a_at | -2.06294 | AK069916 // Oryza sativa Japonica Group cDNA clone:J023038C16, full insert seque | 0.00156884 | 0.05483 |
| Os.26576.1.S1_at | -2.06291 | LOC_Os03g56730.1 // expressed protein // rgap // 11 // --- /// LOC_Os03g56730.2 | 0.00390095 | 0.03877 |
| Os.26970.1.S1_at | -2.06271 | LOC_Os03g35849.1 // expressed protein // rgap // 11 // --- /// NM_001057017 // O | 0.00947769 | 0.05211 |
| OsAffx.22295.2.S1_at | -2.06266 | BGIOSSBCE023209.1 // --- // bgi_ris // 11 // --- /// BGIOSSFCE023503.1 // --- // | 0.00754012 | 0.04804 |
| Os.7026.1.S1_x_at | -2.06261 | AK069283 // Oryza sativa Japonica Group cDNA clone:J023012J07, full insert seque | 0.0110241 | 0.06687 |
| Os.52302.2.S1_s_at | -2.05984 | AK101028 // Oryza sativa Japonica Group cDNA clone:J023150G18, full insert seque | 0.0034603 | 0.03931 |
| Os.51681.1.S1_x_at | -2.05931 | LOC_Os06g38430.1 // expressed protein // rgap // 11 // --- /// NM_001064476 // O | 0.00210168 | 0.03562 |
| OsAffx.2518.1.A1_s_at | -2.05868 | NM_001052472 // Oryza sativa (japonica cultivar-group) Os02g0155000 (Os02g015500 | 0.0210826 | 0.08544 |
| Os.7137.2.S1_x_at | -2.05833 | AK067623 // Oryza sativa Japonica Group cDNA clone:J013108L20, full insert seque | 0.0118545 | 0.06859 |
| Os.49396.1.S2_at | -2.05778 | AK066435 // Oryza sativa Japonica Group cDNA clone:J013063I04, full insert seque | 0.00538638 | 0.04746 |
| Os.38268.2.S1_s_at | -2.05768 | AK071428 // Oryza sativa Japonica Group cDNA clone:J023092F23, full insert seque | 0.00417724 | 0.03943 |
| Os.29008.1.S1_x_at | -2.05431 | AK110770 // Oryza sativa Japonica Group cDNA clone:002-171-A10, full insert sequ | 0.00786996 | 0.06761 |
| Os.52172.1.S1_x_at | -2.05414 | AK102311 // Oryza sativa Japonica Group cDNA clone:J033090D11, full insert seque | 0.0110474 | 0.05394 |
| Os.7137.1.S1_at | -2.05402 | LOC_Os01g66980.1 // expressed protein // rgap // 11 // --- /// LOC_Os01g66980.2 | 0.0190511 | 0.07005 |
| OsAffx.17030.1.S1_at | -2.05332 | BGIOSSFCE025596.1 // --- // bgi_ris // 11 // --- /// LOC_Os08g16670.1 // OsFBX28 | 0.000569639 | 0.06182 |
| Os.26537.1.S1_a_at | -2.05238 | AK070499 // Oryza sativa Japonica Group cDNA clone:J023054O08, full insert seque | 0.0209381 | 0.07491 |
| Os.46308.1.S2_s_at | -2.04909 | AK099883 // Oryza sativa Japonica Group cDNA clone:J013111P14, full insert seque | 0.00201274 | 0.03825 |
| Os.33025.1.S1_at | -2.04797 | AK102055 // Oryza sativa Japonica Group cDNA clone:J033082C21, full insert seque | 0.0276629 | 0.07797 |
| Os.52086.1.S1_at | -2.04787 | NM_001061388 // Oryza sativa (japonica cultivar-group) Os05g0193600 (Os05g019360 | 0.000469838 | 0.0433 |
| Os.33610.3.S1_x_at | -2.04735 | AK072454 // Oryza sativa Japonica Group cDNA clone:J023114O11, full insert seque | 0.00221493 | 0.04018 |
| OsAffx.11749.1.S1_x_at | -2.04611 | LOC_Os01g65970.1 // transcription factor HBP-1b, putative // rgap // 11 // --- / | 0.00887483 | 0.1001 |
| Os.54116.1.S1_at | -2.04595 | AK102041 // Oryza sativa Japonica Group cDNA clone:J033081H13, full insert seque | 0.0159594 | 0.06502 |
| Os.49347.1.S1_at | -2.04566 | AK102157 // Oryza sativa Japonica Group cDNA clone:J033086F18, full insert seque | 0.00689077 | 0.04652 |
| Os.51062.1.S1_at | -2.04491 | AK060473 // Oryza sativa Japonica Group cDNA clone:001-012-E10, full insert sequ | 0.00928365 | 0.05085 |
| Os.57126.1.S1_at | -2.04397 | LOC_Os08g38760.1 // expressed protein // rgap // 11 // --- /// NM_001068670 // O | 0.0217607 | 0.0719 |
| OsAffx.5016.1.S1_at | -2.04383 | BGIOSSFCE020754.1 // --- // bgi_ris // 11 // --- /// LOC_Os06g35020.1 // hypothe | 0.00141481 | 0.03677 |
| Os.38301.1.S1_s_at | -2.04293 | AK068507 // Oryza sativa Japonica Group cDNA clone:J013154C12, full insert seque | 0.00787347 | 0.04849 |
| OsAffx.7787.1.S1_at | -2.04083 | BGIOSSBCE034378.1 // --- // bgi_ris // 11 // --- /// BGIOSSFCE034926.1 // --- // | 0.0148048 | 0.1287 |
| Os.52637.1.S1_x_at | -2.04015 | AK065967 // Oryza sativa Japonica Group cDNA clone:J013049E01, full insert seque | 0.00348171 | 0.07253 |
| Os.48879.1.S1_x_at | -2.03843 | AK068648 // Oryza sativa Japonica Group cDNA clone:J013159B10, full insert seque | 0.0380335 | 0.1013 |
| Os.27122.1.S1_a_at | -2.03611 | AK063388 // Oryza sativa Japonica Group cDNA clone:001-114-G11, full insert sequ | 0.00282369 | 0.04257 |
| OsAffx.13360.1.S1_at | -2.03584 | LOC_Os03g45280.1 // dehydrin, putative, expressed // rgap // 11 // --- | 0.0364788 | 0.1978 |
| Os.33868.1.S1_at | -2.03014 | AK073714 // Oryza sativa Japonica Group cDNA clone:J033055I03, full insert seque | 0.00361947 | 0.112 |
| Os.11155.1.S1_at | -2.02984 | AB104733 // Oryza sativa Japonica Group OsTUB8 mRNA for beta-tubulin, complete c | 0.0126616 | 0.06277 |
| Os.17273.1.S1_at | -2.02959 | LOC_Os05g27010.1 // peptide transporter PTR3-A, putative, expressed // rgap // 1 | 0.0120079 | 0.1096 |
| OsAffx.28395.1.S1_at | -2.02844 | BGIOSSFCE022576.1 // --- // bgi_ris // 11 // --- /// LOC_Os07g10860.1 // conserv | 0.0245519 | 0.09007 |
| Os.53542.1.S1_x_at | -2.02826 | CT832028 // Oryza sativa (indica cultivar-group) cDNA clone:OSIGCPI020I24, full | 0.0134336 | 0.06747 |
| Os.50739.1.S1_at | -2.02631 | AY220688 // Oryza sativa box C/D snoRNA J50.1, complete sequence. // gb // 11 // | 0.00186173 | 0.04055 |
| Os.22825.1.A1_at | -2.02546 | BGIOSSFCE011937.1 // --- // bgi_ris // 0 // chr3:29742113-29748891(-) /// LOC_Os | 0.000835632 | 0.02567 |
| OsAffx.14181.1.S1_at | -2.02333 | BGIOSSBCE014167.1 // --- // bgi_ris // 11 // --- /// LOC_Os04g37940.1 // express | 0.0219061 | 0.0782 |
| Os.17695.1.S1_at | -2.02277 | LOC_Os06g45330.1 // endonuclease V, putative, expressed // rgap // 9 // --- | 0.0123877 | 0.07684 |
| Os.18164.1.S1_at | -2.02202 | NM_001058742 // Oryza sativa (japonica cultivar-group) Os04g0192800 (Os04g019280 | 0.0288744 | 0.08323 |
| Os.46308.1.S1_s_at | -2.02069 | AK099883 // Oryza sativa Japonica Group cDNA clone:J013111P14, full insert seque | 0.00272644 | 0.03629 |
| Os.47737.1.S1_at | -2.02069 | LOC_Os02g48450.1 // xylem cysteine proteinase 2 precursor, putative, expressed / | 0.0398923 | 0.1114 |
| Os.26862.3.S1_a_at | -2.01784 | AK240637 // Oryza sativa Japonica Group cDNA, clone: J013047L22, full insert seq | 0.0208775 | 0.07545 |
| OsAffx.24874.1.S1_at | -2.01747 | AK287814 // Oryza sativa Japonica Group cDNA, clone: J065180F24, full insert seq | 0.0000582 | 0.04669 |
| Os.54008.1.S1_at | -2.01678 | AK101257 // Oryza sativa Japonica Group cDNA clone:J033032G23, full insert seque | 0.00280414 | 0.04402 |
| Os.27362.1.S2_at | -2.01627 | LOC_Os08g08592.1 // expressed protein // rgap // 11 // --- /// LOC_Os08g08592.2 | 0.0233285 | 0.0953 |
| OsAffx.12160.1.S1_at | -2.01531 | AK241090 // Oryza sativa Japonica Group cDNA, clone: J065075P05, full insert seq | 0.0165787 | 0.06301 |
| Os.22932.1.S1_at | -2.01452 | NM_001059976 // Oryza sativa (japonica cultivar-group) Os04g0540500 (Os04g054050 | 0.0322074 | 0.08371 |
| OsAffx.6270.1.S1_at | -2.01144 | BGIOSSFCE027683.1 // --- // bgi_ris // 11 // --- /// LOC_Os09g14450.1 // pollen | 0.0173255 | 0.07463 |
| Os.27569.1.S1_a_at | -2.01138 | AK120086 // Oryza sativa Japonica Group cDNA clone:J013020J11, full insert seque | 0.00570945 | 0.05003 |
| Os.20295.1.S1_at | -2.01056 | AK066454 // Oryza sativa Japonica Group cDNA clone:J013065K03, full insert seque | 0.00038432 | 0.02857 |
| Os.24213.1.S1_at | -2.00826 | AK100251 // Oryza sativa Japonica Group cDNA clone:J023060O04, full insert seque | 0.00281251 | 0.0446 |
| Os.27512.1.A1_at | -2.00755 | AK069909 // Oryza sativa Japonica Group cDNA clone:J023037G05, full insert seque | 0.0048528 | 0.04398 |
| Os.15307.1.S1_at | -2.00672 | AK070668 // Oryza sativa Japonica Group cDNA clone:J023057B05, full insert seque | 0.0036274 | 0.03825 |
| Os.45041.1.S1_x_at | -2.0065 | AK099933 // Oryza sativa Japonica Group cDNA clone:J013119E02, full insert seque | 0.00106304 | 0.04703 |
| Os.53572.1.S1_at | -2.00434 | AK073356 // Oryza sativa Japonica Group cDNA clone:J033028K22, full insert seque | 0.0424792 | 0.09613 |
| Os.52646.1.S1_at | -2.00192 | AK068517 // Oryza sativa Japonica Group cDNA clone:J013154G13, full insert seque | 0.00713326 | 0.1226 |
| OsAffx.12647.1.S1_at | -2.00037 | BGIOSSFCE008387.1 // --- // bgi_ris // 11 // --- /// LOC_Os02g54930.1 // hypothe | 0.0207799 | 0.06924 |
| Os.27156.1.S1_at | 2.00266 | LOC_Os07g12800.1 // expressed protein // rgap // 11 // --- /// LOC_Os07g12800.2 | 0.00296154 | 0.1409 |
| Os.34151.1.S2_a_at | 2.00328 | AK071254 // Oryza sativa Japonica Group cDNA clone:J023087G23, full insert seque | 0.0133861 | 0.5202 |
| Os.8726.1.S2_at | 2.00653 | AK068243 // Oryza sativa Japonica Group cDNA clone:J013149G12, full insert seque | 0.0090487 | 0.05376 |
| Os.48105.1.A1_at | 2.00767 | LOC_Os06g15620.1 // GASR7 - Gibberellin-regulated GASA/GAST/Snakin family protei | 0.0315895 | 0.099 |
| Os.23405.1.S1_at | 2.00948 | AK100710 // Oryza sativa Japonica Group cDNA clone:J023115F20, full insert seque | 0.0275272 | 0.3703 |
| Os.10423.1.S1_at | 2.01308 | AB074972 // Oryza sativa Japonica Group OsEIL2 mRNA for ethylene-insensitive-3-l | 0.00894308 | 0.1418 |
| Os.11301.1.S1_at | 2.01765 | AK070778 // Oryza sativa Japonica Group cDNA clone:J023069E14, full insert seque | 0.011939 | 0.3899 |
| Os.17998.1.S1_at | 2.01809 | AK073577 // Oryza sativa Japonica Group cDNA clone:J033047C15, full insert seque | 0.0036102 | 0.2718 |
| Os.11773.1.S1_at | 2.01905 | AB190436 // Oryza sativa Japonica Group OsWRKY53 mRNA for transcription factor O | 0.0264717 | 0.2024 |
| Os.316.1.S1_at | 2.02232 | AK099079 // Oryza sativa Japonica Group cDNA clone:J023001I07, full insert seque | 0.0166803 | 0.4397 |
| OsAffx.12869.1.S1_s_at | 2.02339 | LOC_Os03g14950.1 // PLA IIIA/PLP7, putative, expressed // rgap // 11 // --- | 0.00353754 | 0.2863 |
| Os.24699.1.S1_at | 2.02422 | LOC_Os02g47190.1 // MYB family transcription factor, putative, expressed // rgap | 0.0300827 | 0.4251 |
| Os.11549.1.S1_at | 2.02458 | AK069456 // Oryza sativa Japonica Group cDNA clone:J023019E08, full insert seque | 0.0164346 | 0.4258 |
| Os.11781.1.S1_at | 2.02678 | AK072062 // Oryza sativa Japonica Group cDNA clone:J013109E06, full insert seque | 0.00249555 | 0.05715 |
| Os.21932.1.S1_at | 2.02702 | CT833247 // Oryza sativa (indica cultivar-group) cDNA clone:OSIGCPI241J15, full | 0.0170199 | 0.2552 |
| Os.53936.1.S1_at | 2.02733 | AK100934 // Oryza sativa Japonica Group cDNA clone:J023136E13, full insert seque | 0.0320772 | 0.2468 |
| Os.25588.1.S1_a_at | 2.02796 | AY323480 // Oryza sativa (japonica cultivar-group) putative myb protein (MYB) mR | 0.00423933 | 0.1521 |
| Os.27614.1.S1_at | 2.0308 | AK071504 // Oryza sativa Japonica Group cDNA clone:J023094A01, full insert seque | 0.00811058 | 0.06986 |
| Os.3388.1.S1_x_at | 2.03101 | AK111798 // Oryza sativa Japonica Group cDNA clone:J023105H16, full insert seque | 0.00687513 | 0.08279 |
| Os.8789.1.S1_at | 2.03417 | AK064971 // Oryza sativa Japonica Group cDNA clone:J013001B15, full insert seque | 0.00126208 | 0.3792 |
| Os.52766.1.S1_at | 2.03659 | LOC_Os01g41310.1 // OsFBX18 - F-box domain containing protein, expressed // rgap | 0.00655145 | 0.04679 |
| Os.27642.1.S1_at | 2.03744 | CT831427 // Oryza sativa (indica cultivar-group) cDNA clone:OSIGCSA005A07, full | 0.0225789 | 0.2864 |
| Os.165.1.S1_at | 2.03865 | CT828610 // Oryza sativa (indica cultivar-group) cDNA clone:OSIGCSN025O07, full | 0.0301645 | 0.1578 |
| Os.21858.1.S1_at | 2.04077 | AK071600 // Oryza sativa Japonica Group cDNA clone:J023105E08, full insert seque | 0.00583814 | 0.2389 |
| Os.10315.1.S1_at | 2.04296 | LOC_Os11g25454.1 // cytokinin-N-glucosyltransferase 1, putative, expressed // rg | 0.00307091 | 0.2655 |
| Os.30376.1.S1_at | 2.04542 | LOC_Os01g02130.1 // expressed protein // rgap // 11 // --- /// NM_001048342 // O | 0.0218187 | 0.2977 |
| Os.56045.1.S1_at | 2.04913 | AK109168 // Oryza sativa Japonica Group cDNA clone:002-155-H03, full insert sequ | 0.0133119 | 0.2217 |
| Os.6023.1.S1_s_at | 2.05101 | AK071306 // Oryza sativa Japonica Group cDNA clone:J023088C20, full insert seque | 0.00226973 | 0.1151 |
| Os.38235.2.S1_at | 2.05109 | LOC_Os04g52260.1 // LTPL124 - Protease inhibitor/seed storage/LTP family protein | 0.00854071 | 0.1815 |
| OsAffx.14324.1.S1_at | 2.05155 | AK241934 // Oryza sativa Japonica Group cDNA, clone: J075082P08, full insert seq | 0.0358548 | 0.1665 |
| Os.37430.1.S1_at | 2.05274 | LOC_Os11g04720.2 // OsRR10 type-A response regulator, expressed // rgap // 11 / | 0.0445488 | 0.2689 |
| Os.10172.1.S1_at | 2.05394 | LOC_Os02g41510.1 // MYB family transcription factor, putative, expressed // rgap | 0.00556539 | 0.3533 |
| Os.14592.1.S1_at | 2.05514 | AK060274 // Oryza sativa Japonica Group cDNA clone:001-005-G07, full insert sequ | 0.00122659 | 0.03803 |
| Os.26133.1.S1_at | 2.05574 | AK070986 // Oryza sativa Japonica Group cDNA clone:J023078F08, full insert seque | 0.00345993 | 0.3699 |
| Os.9273.1.S1_x_at | 2.05889 | AK241092 // Oryza sativa Japonica Group cDNA, clone: J065076A08, full insert seq | 0.020875 | 0.1944 |
| Os.20151.1.S1_at | 2.05952 | LOC_Os03g09900.1 // membrane protein, putative, expressed // rgap // 11 // --- / | 0.000738371 | 0.5636 |
| Os.37849.1.A1_s_at | 2.06396 | AK068289 // Oryza sativa Japonica Group cDNA clone:J013147E18, full insert seque | 0.00690281 | 0.2038 |
| Os.30634.1.S1_at | 2.06611 | LOC_Os01g51690.1 // OsWRKY59 - Superfamily of TFs having WRKY and zinc finger do | 0.0365027 | 0.3351 |
| Os.6723.1.S1_s_at | 2.07022 | AK073251 // Oryza sativa Japonica Group cDNA clone:J033022H17, full insert seque | 0.0164 | 0.8386 |
| OsAffx.4107.1.S1_s_at | 2.07433 | LOC_Os04g46350.1 // homeobox associated leucine zipper, putative, expressed // r | 0.00594316 | 0.17 |
| Os.57356.1.S1_at | 2.07525 | AK111362 // Oryza sativa Japonica Group cDNA clone:002-181-H07, full insert sequ | 0.013418 | 0.07228 |
| Os.26787.3.S1_at | 2.07538 | LOC_Os06g36270.1 // receptor-like protein kinase 5 precursor, putative, expresse | 0.0272297 | 0.4252 |
| Os.8957.1.S1_at | 2.07659 | AK071523 // Oryza sativa Japonica Group cDNA clone:J023100N13, full insert seque | 0.0040235 | 0.1406 |
| Os.4863.2.S1_at | 2.07991 | AK072497 // Oryza sativa Japonica Group cDNA clone:J023125G17, full insert seque | 0.0187692 | 0.1697 |
| Os.49539.1.S1_at | 2.08024 | AK109319 // Oryza sativa Japonica Group cDNA clone:002-188-G11, full insert sequ | 0.0013789 | 0.05951 |
| Os.25588.2.S1_x_at | 2.08324 | EU009207 // Oryza sativa (indica cultivar-group) putative MYB transcription fact | 0.00152059 | 0.1656 |
| Os.7115.1.S1_a_at | 2.08522 | AK288688 // Oryza sativa Japonica Group cDNA, clone: J090060H19, full insert seq | 0.0191931 | 0.2936 |
| Os.49517.1.S1_at | 2.08643 | AK120642 // Oryza sativa Japonica Group cDNA clone:J013154O20, full insert seque | 0.00184959 | 0.4104 |
| Os.51926.1.S1_x_at | 2.08749 | AK064292 // Oryza sativa Japonica Group cDNA clone:002-105-H07, full insert sequ | 0.0102934 | 0.08879 |
| Os.28798.1.S1_at | 2.08764 | CT833215 // Oryza sativa (indica cultivar-group) cDNA clone:OSIGCSA047I02, full | 0.00969125 | 0.4412 |
| OsAffx.31481.1.S1_at | 2.08768 | LOC_Os11g44890.1 // expressed protein // rgap // 11 // --- /// Os11g0672500 // - | 0.00213641 | 0.03629 |
| Os.7862.1.S1_at | 2.0909 | AK111568 // Oryza sativa Japonica Group cDNA clone:J013072L11, full insert seque | 0.000540452 | 0.1106 |
| OsAffx.2891.1.S1_s_at | 2.096 | LOC_Os02g37330.1 // heavy metal associated domain containing protein, expressed | 0.00039454 | 0.1705 |
| Os.7335.1.S1_at | 2.09644 | AK069475 // Oryza sativa Japonica Group cDNA clone:J023020F21, full insert seque | 0.000150908 | 0.1961 |
| Os.11808.1.S1_at | 2.09814 | CT830405 // Oryza sativa (indica cultivar-group) cDNA clone:OSIGCRA115G10, full | 0.000878544 | 0.4392 |
| Os.1665.1.S1_a_at | 2.09989 | AB051107 // Oryza sativa RIP1 mRNA for ribosome inactivating protein 1, complete | 0.0358574 | 0.2205 |
| Os.11301.1.S1_x_at | 2.10504 | AK070778 // Oryza sativa Japonica Group cDNA clone:J023069E14, full insert seque | 0.00701967 | 0.1665 |
| Os.10747.1.S1_s_at | 2.10634 | AK241364 // Oryza sativa Japonica Group cDNA, clone: J065152E11, full insert seq | 0.0137646 | 0.322 |
| Os.17805.1.S1_at | 2.1137 | AK119283 // Oryza sativa Japonica Group cDNA clone:001-130-C08, full insert sequ | 0.0179399 | 0.182 |
| Os.4020.1.S1_at | 2.11426 | AK059636 // Oryza sativa Japonica Group cDNA clone:001-031-A09, full insert sequ | 0.0384726 | 0.1554 |
| Os.27724.2.A1_a_at | 2.11438 | LOC_Os02g43340.1 // hydrolase, alpha/beta fold family domain containing protein, | 0.00878715 | 0.07355 |
| Os.34913.1.S1_at | 2.11446 | AK069648 // Oryza sativa Japonica Group cDNA clone:J023026P15, full insert seque | 0.019792 | 0.4225 |
| Os.51506.1.S1_at | 2.11507 | LOC_Os03g20600.1 // expressed protein // rgap // 11 // --- /// NM_001056501 // O | 0.01338 | 0.2711 |
| Os.56892.1.S1_at | 2.11912 | LOC_Os09g35850.1 // RNA recognition motif containing protein, putative, expresse | 0.00409977 | 0.04684 |
| Os.48875.1.S1_at | 2.12691 | AB180689 // Oryza sativa Japonica Group OsGS1;3 mRNA for cytosolic glutamine syn | 0.0160956 | 0.1996 |
| OsAffx.24682.1.S1_s_at | 2.12865 | AK059045 // Oryza sativa Japonica Group cDNA clone:001-021-D08, full insert sequ | 0.00338446 | 0.3217 |
| Os.43043.1.S1_at | 2.13045 | CT834995 // Oryza sativa (indica cultivar-group) cDNA clone:OSIGCSN047D16, full | 0.0175397 | 0.07463 |
| Os.53428.1.S1_at | 2.13198 | LOC_Os09g26370.1 // DUF581 domain containing protein, expressed // rgap // 11 // | 0.00495101 | 0.06619 |
| Os.11962.1.S1_at | 2.13315 | AF402804 // Oryza sativa (japonica cultivar-group) clone E3743_1A putative gluta | 0.000733287 | 0.1405 |
| Os.681.1.S1_at | 2.13461 | LOC_Os01g11860.1 // DJ-1 family protein, putative, expressed // rgap // 11 // -- | 0.00599781 | 0.2834 |
| OsAffx.27508.7.S1_s_at | 2.13712 | AK110901 // Oryza sativa Japonica Group cDNA clone:002-173-B02, full insert sequ | 0.00162491 | 0.3094 |
| Os.27786.1.S1_at | 2.13753 | AK120297 // Oryza sativa Japonica Group cDNA clone:J013051O08, full insert seque | 0.000809079 | 0.2314 |
| Os.26889.1.S1_at | 2.14172 | AK071141 // Oryza sativa Japonica Group cDNA clone:J023082D02, full insert seque | 0.00814707 | 0.3905 |
| Os.51246.1.S1_at | 2.14294 | LOC_Os11g11780.1 // cysteine-rich receptor-like protein kinase 21 precursor, put | 0.0236918 | 0.3679 |
| Os.54282.1.S1_x_at | 2.14761 | AK103049 // Oryza sativa Japonica Group cDNA clone:J033117G22, full insert seque | 0.042784 | 0.4114 |
| OsAffx.13437.1.S1_at | 2.14956 | CT835290 // Oryza sativa (indica cultivar-group) cDNA clone:OSIGCSN089D14, full | 0.00440755 | 0.1428 |
| Os.53162.1.S1_s_at | 2.15137 | AK071118 // Oryza sativa Japonica Group cDNA clone:J023082L01, full insert seque | 0.0482516 | 0.2317 |
| Os.6581.1.S1_at | 2.1536 | LOC_Os10g43060.1 // expressed protein // rgap // 10 // --- | 0.00640025 | 0.4512 |
| OsAffx.16971.1.S1_at | 2.15539 | BGIOSSBCE025088.1 // --- // bgi_ris // 11 // --- /// LOC_Os08g13090.1 // BTB/POZ | 0.00248123 | 0.05798 |
| Os.11250.1.S1_at | 2.15842 | LOC_Os02g13800.1 // HSF-type DNA-binding domain containing protein, expressed // | 0.00896106 | 0.1693 |
| Os.11987.1.S1_at | 2.15849 | CT831856 // Oryza sativa (indica cultivar-group) cDNA clone:OSIGCPI220N19, full | 0.0152392 | 0.06096 |
| Os.8994.1.S1_at | 2.16075 | AK069135 // Oryza sativa Japonica Group cDNA clone:J023007E22, full insert seque | 0.00548575 | 0.1556 |
| Os.9660.1.S1_at | 2.16093 | CT828611 // Oryza sativa (indica cultivar-group) cDNA clone:OSIGCRA108H01, full | 0.00856144 | 0.06526 |
| Os.21243.1.S1_at | 2.16177 | AK065365 // Oryza sativa Japonica Group cDNA clone:J013003K19, full insert seque | 0.00186361 | 0.0592 |
| Os.11816.1.S1_at | 2.16268 | LOC_Os04g37490.1 // oxidoreductase, aldo/keto reductase family protein, putative | 0.010031 | 0.1908 |
| Os.34020.2.S1_at | 2.16379 | LOC_Os01g10960.1 // peptide-N4-asparagine amidase A, putative // rgap // 0 // ch | 0.000227951 | 0.3431 |
| Os.19069.1.S2_x_at | 2.16884 | NM_001074901 // Oryza sativa (japonica cultivar-group) Os11g0645600 (Os11g064560 | 0.00263462 | 0.0446 |
| Os.322.1.S1_at | 2.16922 | AK066232 // Oryza sativa Japonica Group cDNA clone:J013057M05, full insert seque | 0.000697061 | 0.1895 |
| Os.33787.1.S1_at | 2.17122 | AK073030 // Oryza sativa Japonica Group cDNA clone:J023144D17, full insert seque | 0.0355893 | 0.2646 |
| Os.21318.1.S1_at | 2.17128 | AK069316 // Oryza sativa Japonica Group cDNA clone:J023013C22, full insert seque | 0.00352647 | 0.04741 |
| Os.12603.1.S1_at | 2.17515 | AK104148 // Oryza sativa Japonica Group cDNA clone:006-208-B05, full insert sequ | 0.00277978 | 0.1314 |
| Os.9073.1.S1_at | 2.17545 | LOC_Os01g57470.1 // EF hand family protein, putative, expressed // rgap // 11 // | 0.00169574 | 0.2585 |
| Os.27624.1.S1_at | 2.17747 | LOC_Os03g14590.1 // calcium-binding EF hand family protein, putative, expressed | 0.0295827 | 0.1499 |
| Os.52791.1.S1_at | 2.17933 | AK069270 // Oryza sativa Japonica Group cDNA clone:J023013P18, full insert seque | 0.00636987 | 0.4091 |
| Os.26903.1.S1_at | 2.18021 | AK111840 // Oryza sativa Japonica Group cDNA clone:J023139I03, full insert seque | 0.00760231 | 0.12 |
| Os.28531.1.S1_at | 2.18484 | LOC_Os01g64100.1 // glycosyl hydrolase, putative, expressed // rgap // 11 // --- | 0.0168349 | 0.1891 |
| Os.53592.1.S1_s_at | 2.18637 | AK073461 // Oryza sativa Japonica Group cDNA clone:J033040O17, full insert seque | 0.00442918 | 0.09676 |
| Os.51661.1.S1_at | 2.19096 | LOC_Os01g64330.1 // retrotransposon protein, putative, unclassified, expressed / | 0.000996371 | 0.04435 |
| Os.38235.1.S1_at | 2.19247 | CT829944 // Oryza sativa (indica cultivar-group) cDNA clone:OSIGCSN008A11, full | 0.00276058 | 0.2293 |
| Os.16933.1.S1_at | 2.19295 | LOC_Os01g61990.1 // ankyrin repeat-containing protein, putative, expressed // rg | 0.0200219 | 0.2571 |
| Os.4863.1.S1_at | 2.19297 | AK072497 // Oryza sativa Japonica Group cDNA clone:J023125G17, full insert seque | 0.0189717 | 0.241 |
| Os.20501.1.S1_at | 2.19757 | AK120859 // Oryza sativa Japonica Group cDNA clone:J023024E09, full insert seque | 0.0119026 | 0.3206 |
| Os.4866.1.S1_at | 2.19983 | LOC_Os01g31980.1 // MATE efflux family protein, putative, expressed // rgap // 1 | 0.00646914 | 0.2956 |
| Os.11468.1.S1_at | 2.20167 | CT829884 // Oryza sativa (indica cultivar-group) cDNA clone:OSIGCPI202N12, full | 0.00792739 | 0.2974 |
| Os.11331.1.S1_at | 2.20591 | AK062655 // Oryza sativa Japonica Group cDNA clone:001-105-E11, full insert sequ | 0.0287084 | 0.1181 |
| Os.7890.1.S1_x_at | 2.2099 | AF094775 // Oryza sativa chlorophyll a/b-binding protein presursor (Cab27) mRNA, | 0.0389569 | 0.2556 |
| Os.27387.2.S1_x_at | 2.2151 | BGIOSSBCE001217.1 // --- // bgi_ris // 0 // chr1:10007445-10014252(-) /// BGIOSS | 0.0472516 | 0.2731 |
| Os.27177.1.S1_at | 2.21512 | LOC_Os03g16390.1 // avr9/Cf-9 rapidly elicited protein, putative, expressed // r | 0.000356904 | 0.1376 |
| Os.55337.1.S1_at | 2.21752 | LOC_Os03g25680.1 // expressed protein // rgap // 11 // --- /// NM_001056744 // O | 0.00106153 | 0.4288 |
| Os.11945.1.S1_at | 2.22118 | LOC_Os08g29660.1 // OsWRKY69 - Superfamily of TFs having WRKY and zinc finger do | 0.0207073 | 0.4578 |
| Os.7935.2.S1_x_at | 2.22496 | CT833769 // Oryza sativa (indica cultivar-group) cDNA clone:OSIGCEA018M12, full | 0.00301667 | 0.2878 |
| Os.4717.1.S1_at | 2.22781 | AK101662 // Oryza sativa Japonica Group cDNA clone:J033057L03, full insert seque | 0.0223797 | 0.0769 |
| OsAffx.29580.1.S1_at | 2.23219 | CT835962 // Oryza sativa (indica cultivar-group) cDNA clone:OSIGCRA108K23, full | 0.00490101 | 0.08347 |
| Os.53795.1.S1_at | 2.23469 | CT831232 // Oryza sativa (indica cultivar-group) cDNA clone:OSIGCRA129J17, full | 0.0000546 | 0.3766 |
| Os.4893.1.S1_at | 2.2351 | AK100184 // Oryza sativa Japonica Group cDNA clone:J023031H12, full insert seque | 0.034676 | 0.2157 |
| OsAffx.28023.2.S1_at | 2.23642 | BGIOSSFCE021067.1 // --- // bgi_ris // 11 // --- /// LOC_Os06g40420.1 // hypothe | 0.00246757 | 0.03449 |
| Os.55273.1.S1_at | 2.23712 | NM_001059309 // Oryza sativa (japonica cultivar-group) Os04g0419100 (Os04g041910 | 0.00000373 | 0.34 |
| Os.39617.1.S1_at | 2.23784 | AK120390 // Oryza sativa Japonica Group cDNA clone:J013073P15, full insert seque | 0.000282297 | 0.149 |
| Os.17445.1.S1_s_at | 2.2404 | LOC_Os03g27080.1 // calmodulin-binding transcription activator 2, putative, expr | 0.00531723 | 0.3006 |
| Os.45749.1.A1_at | 2.24371 | LOC_Os01g20910.1 // zinc finger, C3HC4 type domain containing protein, expressed | 0.018987 | 0.08065 |
| Os.3387.1.S1_at | 2.2452 | LOC_Os10g33810.1 // myb-related protein Myb4, putative, expressed // rgap // 11 | 0.00126866 | 0.3375 |
| Os.30608.3.S1_x_at | 2.25312 | AK072410 // Oryza sativa Japonica Group cDNA clone:J023083A20, full insert seque | 0.0351206 | 0.4146 |
| Os.27946.1.S1_at | 2.25525 | LOC_Os04g51320.1 // transcription factor TF2, putative, expressed // rgap // 11 | 0.00179108 | 0.2063 |
| Os.5736.1.S1_s_at | 2.25719 | AK068588 // Oryza sativa Japonica Group cDNA clone:J013152P10, full insert seque | 0.011862 | 0.1071 |
| Os.53792.1.S1_at | 2.26171 | LOC_Os11g44330.1 // expressed protein // rgap // 11 // --- /// NM_001074949 // O | 0.00614529 | 0.04741 |
| Os.52728.1.S1_at | 2.26424 | LOC_Os04g37880.1 // expressed protein // rgap // 11 // --- /// NM_001059482 // O | 0.0032077 | 0.0654 |
| OsAffx.14364.1.S1_s_at | 2.26462 | BGIOSSFCE015363.1 // --- // bgi_ris // 11 // --- /// LOC_Os04g51320.1 // transcr | 0.000803535 | 0.2567 |
| Os.7664.1.S1_at | 2.26643 | CT830358 // Oryza sativa (indica cultivar-group) cDNA clone:OSIGCSA031L22, full | 0.0270424 | 0.3123 |
| Os.33742.1.S1_at | 2.26828 | LOC_Os11g32750.1 // hydrolase, NUDIX family, domain containing protein, expresse | 0.0253527 | 0.1785 |
| Os.53440.1.S1_at | 2.27627 | AK073663 // Oryza sativa Japonica Group cDNA clone:J033046A21, full insert seque | 0.0142917 | 0.1303 |
| Os.17746.1.S1_s_at | 2.27647 | AK068362 // Oryza sativa Japonica Group cDNA clone:J013145L16, full insert seque | 0.000926195 | 0.443 |
| Os.50680.1.S1_at | 2.2826 | AJ490482 // Oryza sativa z118c snoRNA. // gb // 11 // --- | 0.0203634 | 0.07453 |
| OsAffx.31257.1.S1_s_at | 2.28265 | BGIOSSBCE032226.1 // --- // bgi_ris // 11 // --- /// LOC_Os11g31950.1 // express | 0.00621336 | 0.4173 |
| Os.5117.1.S1_at | 2.28345 | AK111124 // Oryza sativa Japonica Group cDNA clone:002-176-C05, full insert sequ | 0.00604504 | 0.2397 |
| Os.15426.1.S1_at | 2.29585 | AK103518 // Oryza sativa Japonica Group cDNA clone:J033131H17, full insert seque | 0.0235418 | 0.5354 |
| OsAffx.19490.2.S1_at | 2.29653 | BGIOSSBCE033276.1 // --- // bgi_ris // 11 // --- /// BGIOSSFCE033806.1 // --- // | 0.013635 | 0.1307 |
| OsAffx.7301.1.S1_at | 2.29812 | LOC_Os11g35410.1 // expressed protein // rgap // 11 // --- /// NM_001074630 // O | 0.00410097 | 0.5916 |
| Os.50585.1.S1_at | 2.3002 | AK121953 // Oryza sativa Japonica Group cDNA clone:J033107L05, full insert seque | 0.000615281 | 0.1462 |
| Os.9982.1.S1_at | 2.30668 | AK073278 // Oryza sativa Japonica Group cDNA clone:J033025L08, full insert seque | 0.0344457 | 0.3153 |
| Os.6516.1.S1_at | 2.3089 | LOC_Os07g33280.1 // expressed protein // rgap // 11 // --- | 0.0236642 | 0.4074 |
| Os.54867.1.S1_at | 2.31439 | AK106955 // Oryza sativa Japonica Group cDNA clone:002-119-E08, full insert sequ | 0.00790292 | 0.182 |
| Os.50249.1.S1_x_at | 2.31507 | AK066472 // Oryza sativa Japonica Group cDNA clone:J013066N20, full insert seque | 0.000748729 | 0.1803 |
| Os.11900.1.S1_at | 2.31578 | LOC_Os02g37930.1 // hypoxia-responsive family protein, putative, expressed // rg | 0.0125495 | 0.2283 |
| Os.22940.1.S1_at | 2.32378 | AK069371 // Oryza sativa Japonica Group cDNA clone:J023013P12, full insert seque | 0.0105573 | 0.5952 |
| Os.5187.1.S1_at | 2.32409 | AK062162 // Oryza sativa Japonica Group cDNA clone:001-046-B03, full insert sequ | 0.00275791 | 0.06182 |
| Os.52607.1.S1_at | 2.32692 | AK068286 // Oryza sativa Japonica Group cDNA clone:J013149K15, full insert seque | 0.00264761 | 0.1259 |
| Os.5045.1.S1_at | 2.33236 | AK102138 // Oryza sativa Japonica Group cDNA clone:J033085I16, full insert seque | 0.0438062 | 0.1934 |
| Os.26757.1.A1_at | 2.33648 | LOC_Os11g31950.1 // expressed protein // rgap // 11 // --- | 0.00340562 | 0.327 |
| Os.406.1.S1_a_at | 2.33657 | AF216315 // Oryza sativa MAP kinase 1 mRNA, complete cds. // gb // 11 // --- /// | 0.00333344 | 0.3471 |
| Os.9558.1.S1_s_at | 2.33947 | LOC_Os02g45780.1 // zinc finger, C3HC4 type domain containing protein, expressed | 0.000358367 | 0.2472 |
| Os.11197.2.S1_x_at | 2.34089 | LOC_Os09g30414.1 // aspartic proteinase nepenthesin-2 precursor, putative, expre | 0.0132678 | 0.1956 |
| Os.9212.1.S1_at | 2.34484 | AK102058 // Oryza sativa Japonica Group cDNA clone:J033082D07, full insert seque | 0.000341674 | 0.02613 |
| OsAffx.24782.1.S1_s_at | 2.34782 | AK241201 // Oryza sativa Japonica Group cDNA, clone: J065121M03, full insert seq | 0.0318484 | 0.3903 |
| Os.51127.1.S1_a_at | 2.35054 | AK068176 // Oryza sativa Japonica Group cDNA clone:J013132G13, full insert seque | 0.00544225 | 0.0668 |
| OsAffx.3652.1.S1_at | 2.35442 | AK107781 // Oryza sativa Japonica Group cDNA clone:002-133-C07, full insert sequ | 0.0357435 | 0.2904 |
| Os.46287.1.S1_a_at | 2.35898 | LOC_Os05g40770.1 // leucine-rich repeat family protein, putative, expressed // r | 0.000948313 | 0.4548 |
| Os.6244.1.S1_x_at | 2.35948 | LOC_Os03g13740.1 // immediate-early fungal elicitor protein CMPG1, putative, exp | 0.00587037 | 0.6464 |
| OsAffx.26282.1.S1_s_at | 2.35989 | AK073461 // Oryza sativa Japonica Group cDNA clone:J033040O17, full insert seque | 0.0125988 | 0.1104 |
| Os.28406.1.S1_at | 2.36002 | EU847520 // Oryza sativa Japonica Group clone OsEP4 AP2/ERF domain protein mRNA, | 0.0240758 | 0.2352 |
| OsAffx.4996.1.S1_at | 2.37632 | BGIOSSFCE020700.1 // --- // bgi_ris // 9 // --- /// LOC_Os06g33970.1 // VQ domai | 0.012796 | 0.3268 |
| Os.11673.2.S1_x_at | 2.37673 | AK288466 // Oryza sativa Japonica Group cDNA, clone: J090036M20, full insert seq | 0.0235748 | 0.1584 |
| Os.282.1.S1_a_at | 2.37799 | AB042550 // Oryza sativa Japonica Group mRNA for OsCDPK7, complete cds. // gb // | 0.0000068 | 0.2079 |
| Os.6362.1.S1_at | 2.38593 | AK243228 // Oryza sativa Japonica Group cDNA, clone: J100044F19, full insert seq | 0.00337664 | 0.03877 |
| Os.33087.2.S1_at | 2.38886 | AK100978 // Oryza sativa Japonica Group cDNA clone:J023144E12, full insert seque | 0.000656526 | 0.02741 |
| OsAffx.14465.1.S1_s_at | 2.39465 | LOC_Os04g58120.1 // CRP2 - Cysteine-rich family protein precursor, expressed // | 0.00399042 | 0.2093 |
| Os.20614.2.S1_at | 2.39501 | LOC_Os04g49757.1 // purine permease, putative, expressed // rgap // 11 // --- // | 0.0236633 | 0.2363 |
| Os.15996.1.S1_at | 2.3968 | LOC_Os07g38250.1 // expressed protein // rgap // 11 // --- | 0.00104131 | 0.04703 |
| Os.35024.1.S1_at | 2.40129 | AK069247 // Oryza sativa Japonica Group cDNA clone:J023019B01, full insert seque | 0.000969911 | 0.07545 |
| Os.14959.1.S1_at | 2.4104 | AK065470 // Oryza sativa Japonica Group cDNA clone:J013020L24, full insert seque | 0.00026757 | 0.199 |
| Os.49337.1.S1_at | 2.41458 | LOC_Os02g39140.1 // helix-loop-helix DNA-binding domain containing protein, expr | 0.00151928 | 0.1145 |
| Os.20863.2.S1_x_at | 2.41583 | AK065438 // Oryza sativa Japonica Group cDNA clone:J013025A05, full insert seque | 0.00377965 | 0.2739 |
| Os.17247.1.S1_at | 2.41953 | AK101538 // Oryza sativa Japonica Group cDNA clone:J033048F22, full insert seque | 0.049674 | 0.296 |
| Os.6244.1.S1_at | 2.42001 | LOC_Os03g13740.1 // immediate-early fungal elicitor protein CMPG1, putative, exp | 0.0159089 | 0.6111 |
| Os.5676.1.S2_at | 2.42255 | LOC_Os08g34060.1 // DUF1336 domain containing protein, expressed // rgap // 11 / | 0.00883549 | 0.0502 |
| OsAffx.12954.1.S1_at | 2.42334 | LOC_Os03g20680.1 // late embryogenesis abundant protein 1, putative, expressed / | 0.0019715 | 0.2022 |
| Os.11335.3.S1_x_at | 2.42411 | AK071186 // Oryza sativa Japonica Group cDNA clone:J023086D18, full insert seque | 0.0356081 | 0.2832 |
| Os.53305.1.S1_at | 2.42551 | AK071917 // Oryza sativa Japonica Group cDNA clone:J013071I09, full insert seque | 0.00601826 | 0.273 |
| Os.10872.1.S1_at | 2.44132 | AK065505 // Oryza sativa Japonica Group cDNA clone:J013027N01, full insert seque | 0.000214661 | 0.2299 |
| OsAffx.19322.1.S1_at | 2.44943 | BGIOSSFCE033237.1 // --- // bgi_ris // 11 // --- /// EF532592 // Oryza sativa (i | 0.000305 | 0.04967 |
| Os.5047.1.S1_at | 2.4495 | CT832527 // Oryza sativa (indica cultivar-group) cDNA clone:OSIGCRA117M23, full | 0.00911226 | 0.05107 |
| Os.27718.1.A1_at | 2.45031 | AK241972 // Oryza sativa Japonica Group cDNA, clone: J075095P21, full insert seq | 0.00152218 | 0.0297 |
| Os.34174.1.S1_at | 2.45119 | AK072586 // Oryza sativa Japonica Group cDNA clone:J023132E12, full insert seque | 0.03741 | 0.3314 |
| Os.50104.1.S1_at | 2.45656 | LOC_Os06g40180.1 // phospholipase D, putative, expressed // rgap // 11 // --- // | 0.000504396 | 0.1408 |
| Os.8375.1.S1_at | 2.46446 | AK102045 // Oryza sativa Japonica Group cDNA clone:J033081M13, full insert seque | 0.000658102 | 0.2495 |
| Os.56074.1.S1_at | 2.46584 | LOC_Os02g03710.1 // UP-9A, putative, expressed // rgap // 11 // --- /// NM_00105 | 0.0419915 | 0.1053 |
| Os.49490.1.S1_at | 2.46833 | AK067952 // Oryza sativa Japonica Group cDNA clone:J013127E17, full insert seque | 0.00000709 | 0.01733 |
| OsAffx.27219.1.S1_at | 2.47821 | LOC_Os05g38290.1 // protein phosphatase 2C, putative, expressed // rgap // 11 // | 0.00652809 | 0.08552 |
| Os.2128.1.S1_at | 2.47953 | AK102467 // Oryza sativa Japonica Group cDNA clone:J033094F14, full insert seque | 0.00261244 | 0.3556 |
| Os.26789.1.A1_at | 2.48405 | NM_001075005 // Oryza sativa (japonica cultivar-group) Os11g0685600 (Os11g068560 | 0.0100906 | 0.05621 |
| Os.49803.1.S1_at | 2.48784 | AK111650 // Oryza sativa Japonica Group cDNA clone:J013130K01, full insert seque | 0.00188174 | 0.3828 |
| Os.28397.1.S1_at | 2.49666 | AK120127 // Oryza sativa Japonica Group cDNA clone:J013026F02, full insert seque | 0.022998 | 0.2873 |
| OsAffx.28651.1.S1_x_at | 2.49946 | AK069685 // Oryza sativa Japonica Group cDNA clone:J023030E13, full insert seque | 0.00446968 | 0.129 |
| Os.16139.1.S1_at | 2.50583 | LOC_Os02g48080.1 // cysteine-rich receptor-like protein kinase 7 precursor, puta | 0.0257893 | 0.3172 |
| OsAffx.14458.1.S1_x_at | 2.50587 | AK119671 // Oryza sativa Japonica Group cDNA clone:002-149-H08, full insert sequ | 0.0425513 | 0.281 |
| Os.51005.1.S1_at | 2.50791 | LOC_Os11g05530.1 // expressed protein // rgap // 11 // --- /// NM_001072309 // O | 0.00669455 | 0.1723 |
| Os.34130.1.S1_at | 2.50863 | AK121374 // Oryza sativa Japonica Group cDNA clone:J023127C14, full insert seque | 0.00122098 | 0.1214 |
| Os.37955.2.S1_at | 2.51498 | LOC_Os03g27019.6 // expressed protein // rgap // 11 // --- | 0.0281722 | 0.338 |
| Os.55455.1.S1_at | 2.52483 | LOC_Os04g45370.1 // OsSAUR19 - Auxin-responsive SAUR gene family member, express | 0.0147666 | 0.1603 |
| OsAffx.12587.1.S1_s_at | 2.52774 | LOC_Os02g51320.1 // helix-loop-helix DNA-binding domain containing protein, expr | 0.0127972 | 0.09552 |
| Os.50249.2.S1_at | 2.52873 | AK120329 // Oryza sativa Japonica Group cDNA clone:J013058K09, full insert seque | 0.0107987 | 0.3344 |
| Os.11786.2.S1_at | 2.53069 | LOC_Os01g15900.1 // expressed protein // rgap // 11 // --- | 0.0101271 | 0.06031 |
| Os.8569.1.S1_at | 2.53392 | LOC_Os04g48880.1 // fatty acid hydroxylase, putative, expressed // rgap // 11 // | 0.00248749 | 0.0671 |
| Os.282.2.S1_a_at | 2.53843 | AB042550 // Oryza sativa Japonica Group mRNA for OsCDPK7, complete cds. // gb // | 0.00840351 | 0.2554 |
| Os.7116.1.S1_at | 2.54135 | LOC_Os03g53020.1 // helix-loop-helix DNA-binding domain containing protein, expr | 0.00255575 | 0.1465 |
| Os.11786.1.S1_at | 2.54337 | AK063318 // Oryza sativa Japonica Group cDNA clone:001-113-G11, full insert sequ | 0.00102696 | 0.03228 |
| Os.17974.1.S1_at | 2.54607 | LOC_Os07g01904.1 // expressed protein // rgap // 11 // --- /// LOC_Os07g01904.2 | 0.003349 | 0.03943 |
| Os.52944.1.A1_at | 2.55092 | AK070038 // Oryza sativa Japonica Group cDNA clone:J023035D03, full insert seque | 0.016375 | 0.3807 |
| Os.315.1.S1_at | 2.55279 | AK071180 // Oryza sativa Japonica Group cDNA clone:J023082A15, full insert seque | 0.000765913 | 0.2318 |
| Os.52536.1.S1_at | 2.55279 | AK067801 // Oryza sativa Japonica Group cDNA clone:J013118I15, full insert seque | 0.00146986 | 0.1636 |
| OsAffx.23754.1.S1_x_at | 2.55562 | AK101454 // Oryza sativa Japonica Group cDNA clone:J033040A11, full insert seque | 0.00172273 | 0.1825 |
| Os.24106.3.S1_at | 2.55656 | AK066971 // Oryza sativa Japonica Group cDNA clone:J013089C22, full insert seque | 0.00266779 | 0.3321 |
| Os.16271.1.S1_s_at | 2.55806 | CT836454 // Oryza sativa (indica cultivar-group) cDNA clone:OSIGCSN016M19, full | 0.00271567 | 0.4229 |
| Os.46627.1.S1_at | 2.55864 | AB164463 // Oryza sativa Indica Group OsGGT mRNA for putative glycogenin glucosy | 0.00331333 | 0.3347 |
| Os.57293.1.S1_at | 2.56586 | CT828919 // Oryza sativa (indica cultivar-group) cDNA clone:OSIGCRN134I14, full | 0.0145476 | 0.4783 |
| OsAffx.17366.1.S1_at | 2.56757 | LOC_Os08g36920.1 // AP2 domain containing protein, expressed // rgap // 11 // -- | 0.0399388 | 0.2184 |
| Os.28828.1.S2_at | 2.56826 | AY341844 // Oryza sativa (japonica cultivar-group) WRKY3 mRNA, complete cds. // | 0.00503764 | 0.3405 |
| Os.37729.1.S1_s_at | 2.5801 | AB098712 // Oryza sativa Japonica Group ti mRNA for trypsin inhibitor, complete | 0.0493368 | 0.3653 |
| Os.11786.2.S1_x_at | 2.59185 | AK063318 // Oryza sativa Japonica Group cDNA clone:001-113-G11, full insert sequ | 0.00228687 | 0.04669 |
| OsAffx.26677.1.S1_x_at | 2.59307 | BGIOSSFCE016056.1 // --- // bgi_ris // 11 // --- /// LOC_Os05g01444.1 // polygal | 0.0427732 | 0.3021 |
| Os.33493.1.S1_x_at | 2.59388 | AK100382 // Oryza sativa Japonica Group cDNA clone:J023085F21, full insert seque | 0.00067262 | 0.02828 |
| Os.42024.1.S1_at | 2.59668 | LOC_Os01g56420.1 // ctr copper transporter family protein, putative, expressed / | 0.00141761 | 0.192 |
| Os.49135.1.S1_at | 2.60349 | LOC_Os01g72520.1 // phosphoesterase family protein, putative, expressed // rgap | 0.00256064 | 0.3069 |
| Os.11407.1.S1_at | 2.60624 | CT832589 // Oryza sativa (indica cultivar-group) cDNA clone:OSIGCRA202N08, full | 0.00197263 | 0.2362 |
| Os.11784.1.S1_at | 2.60851 | AK242336 // Oryza sativa Japonica Group cDNA, clone: J075199I04, full insert seq | 0.00181044 | 0.6731 |
| Os.12642.1.S1_at | 2.61332 | EU709762 // Oryza sativa Japonica Group cultivar Zhonghua 15 calcium-dependent p | 0.00659244 | 0.297 |
| Os.25687.1.S1_x_at | 2.61919 | AK068993 // Oryza sativa Japonica Group cDNA clone:J023001A06, full insert seque | 0.0152502 | 0.2471 |
| Os.7911.1.S1_at | 2.62224 | AF309382 // Oryza sativa (japonica cultivar-group) clone C63266_1A putative glut | 0.00869787 | 0.1574 |
| OsAffx.29656.1.S1_at | 2.62431 | AK241696 // Oryza sativa Japonica Group cDNA, clone: J065195P08, full insert seq | 0.00311452 | 0.05756 |
| Os.7938.1.S1_at | 2.6284 | AK065090 // Oryza sativa Japonica Group cDNA clone:J013001L22, full insert seque | 0.00246568 | 0.08292 |
| Os.22968.1.A1_at | 2.6292 | CT836027 // Oryza sativa (indica cultivar-group) cDNA clone:OSIGCSN050M07, full | 0.0441403 | 0.3427 |
| Os.41763.1.S1_at | 2.63122 | AK111885 // Oryza sativa Japonica Group cDNA clone:J033068D10, full insert seque | 0.0269655 | 0.3904 |
| Os.1978.1.S1_at | 2.63652 | AF044260 // Oryza sativa (japonica cultivar-group) clone OsRLK8 receptor serine/ | 0.00453247 | 0.4697 |
| Os.27175.1.S1_a_at | 2.64523 | AY324148 // Oryza sativa (japonica cultivar-group) putative zinc transporter (Os | 0.00587671 | 0.3078 |
| Os.12277.1.S1_at | 2.65014 | EU220429 // Oryza sativa Japonica Group arginine decarboxylase mRNA, complete cd | 0.0014382 | 0.06747 |
| Os.50599.1.S1_x_at | 2.65025 | AK122006 // Oryza sativa Japonica Group cDNA clone:J033110O17, full insert seque | 0.0363064 | 0.2172 |
| Os.15874.1.A1_at | 2.65986 | AY305864 // Oryza sativa (japonica cultivar-group) C2H2 type zinc finger transcr | 0.00283968 | 0.1504 |
| Os.8104.1.S1_a_at | 2.66009 | AB189040 // Oryza sativa Japonica Group OsPRR73 mRNA for pseudo-response regulat | 0.0318456 | 0.1057 |
| Os.44850.1.S1_at | 2.66289 | AK289261 // Oryza sativa Japonica Group cDNA, clone: J100086F23, full insert seq | 0.00148655 | 0.1912 |
| OsAffx.3569.1.S1_x_at | 2.67571 | AK288124 // Oryza sativa Japonica Group cDNA, clone: J080072O22, full insert seq | 0.0126848 | 0.3636 |
| Os.11195.1.S1_at | 2.69411 | CT836095 // Oryza sativa (indica cultivar-group) cDNA clone:OSIGCPI008A05, full | 0.0109533 | 0.4052 |
| Os.4766.1.S1_at | 2.69647 | AF050200 // Oryza sativa acireductone dioxygenase 1 (ARD1) mRNA, complete cds. / | 0.037164 | 0.2217 |
| Os.49571.1.S1_at | 2.69782 | AK060717 // Oryza sativa Japonica Group cDNA clone:001-031-E06, full insert sequ | 0.00101804 | 0.02567 |
| Os.57214.1.S1_at | 2.70159 | LOC_Os08g36970.1 // expressed protein // rgap // 11 // --- /// NM_001068563 // O | 0.000210277 | 0.274 |
| Os.12248.1.S1_at | 2.70459 | LOC_Os07g39680.1 // UV-induced protein uvi15, putative, expressed // rgap // 11 | 0.00978715 | 0.1638 |
| Os.38235.1.S1_x_at | 2.71044 | CT829944 // Oryza sativa (indica cultivar-group) cDNA clone:OSIGCSN008A11, full | 0.0214723 | 0.2997 |
| Os.51817.1.S1_x_at | 2.71136 | AK063851 // Oryza sativa Japonica Group cDNA clone:001-122-C12, full insert sequ | 0.000491605 | 0.02636 |
| Os.47360.1.S1_x_at | 2.71678 | AK072480 // Oryza sativa Japonica Group cDNA clone:J023126F05, full insert seque | 0.0240811 | 0.3537 |
| Os.15757.1.S1_at | 2.71836 | LOC_Os02g39710.1 // CCT/B-box zinc finger protein, putative, expressed // rgap / | 0.00238675 | 0.06395 |
| Os.49819.1.S1_at | 2.72207 | AK111751 // Oryza sativa Japonica Group cDNA clone:J023050J11, full insert seque | 0.0000813 | 0.3549 |
| Os.14998.1.S1_at | 2.73662 | AK100347 // Oryza sativa Japonica Group cDNA clone:J023082L12, full insert seque | 0.0043247 | 0.3007 |
| Os.30203.1.S1_at | 2.73827 | AK099765 // Oryza sativa Japonica Group cDNA clone:J013094O21, full insert seque | 0.000089 | 0.01578 |
| Os.54936.1.S1_at | 2.74305 | AK107136 // Oryza sativa Japonica Group cDNA clone:002-124-C10, full insert sequ | 0.016881 | 0.1653 |
| Os.14405.1.S1_at | 2.7438 | LOC_Os01g04350.1 // hsp20/alpha crystallin family protein, putative, expressed / | 0.00307736 | 0.3163 |
| Os.27807.1.S1_at | 2.75229 | AK063743 // Oryza sativa Japonica Group cDNA clone:001-120-F10, full insert sequ | 0.0286855 | 0.1732 |
| OsAffx.30150.3.S1_at | 2.76375 | AK059600 // Oryza sativa Japonica Group cDNA clone:001-030-E05, full insert sequ | 0.00388928 | 0.04116 |
| Os.57199.1.S1_at | 2.76862 | LOC_Os09g35870.1 // OsIAA26 - Auxin-responsive Aux/IAA gene family member, expre | 0.0135935 | 0.08758 |
| Os.8949.1.S1_at | 2.77189 | LOC_Os08g25140.1 // ethanol tolerance protein GEKO1, putative, expressed // rgap | 0.0304232 | 0.3597 |
| Os.27808.1.S1_at | 2.77212 | AK067697 // Oryza sativa Japonica Group cDNA clone:J013118D08, full insert seque | 0.00027978 | 0.3098 |
| Os.2225.1.S1_at | 2.77673 | AF090698 // Oryza sativa elicitor-responsive gene-3 (ERG3) mRNA, complete cds. / | 0.00885757 | 0.2285 |
| Os.7246.1.S1_s_at | 2.77765 | AK243642 // Oryza sativa Japonica Group cDNA, clone: J100088A21, full insert seq | 0.000393726 | 0.4281 |
| OsAffx.12738.1.S1_x_at | 2.77857 | AK120297 // Oryza sativa Japonica Group cDNA clone:J013051O08, full insert seque | 0.0000825 | 0.2763 |
| OsAffx.12485.1.S1_at | 2.78545 | BGIOSSBCE007422.1 // --- // bgi_ris // 9 // --- /// BGIOSSFCE007552.1 // --- // | 0.00765973 | 0.06859 |
| Os.22278.1.S1_at | 2.78583 | AY078162 // Oryza sativa (indica cultivar-group) phytoene synthase radicle isofo | 0.000039 | 0.07115 |
| Os.11255.1.S1_at | 2.78701 | AY100689 // Oryza sativa glutathione peroxidase 1 (GPX1) mRNA, complete cds. // | 0.000454391 | 0.1331 |
| Os.26411.2.A1_x_at | 2.79452 | AK072505 // Oryza sativa Japonica Group cDNA clone:J023128H01, full insert seque | 0.0354693 | 0.2765 |
| OsAffx.3569.1.S1_at | 2.82295 | AK288124 // Oryza sativa Japonica Group cDNA, clone: J080072O22, full insert seq | 0.0174707 | 0.4968 |
| Os.9840.1.S1_at | 2.82552 | LOC_Os10g17489.1 // UDP-glucoronosyl and UDP-glucosyl transferase domain contain | 0.01379 | 0.4159 |
| Os.15872.1.S1_at | 2.83097 | CT830092 // Oryza sativa (indica cultivar-group) cDNA clone:OSIGCPI029P08, full | 0.00654508 | 0.1854 |
| OsAffx.28023.2.S1_s_at | 2.83251 | BGIOSSFCE021067.1 // --- // bgi_ris // 11 // --- /// LOC_Os01g05770.1 // express | 0.00022709 | 0.02774 |
| Os.11952.1.S1_at | 2.83355 | AK067907 // Oryza sativa Japonica Group cDNA clone:J013127I02, full insert seque | 0.00140227 | 0.5059 |
| Os.20306.1.S1_at | 2.83983 | LOC_Os04g44600.1 // CRP1 - Cysteine-rich family protein precursor, expressed // | 0.000485606 | 0.2102 |
| OsAffx.24612.1.S1_at | 2.84167 | AK243499 // Oryza sativa Japonica Group cDNA, clone: J100074H07, full insert seq | 0.0384128 | 0.3045 |
| Os.32889.1.S1_at | 2.84587 | AK102508 // Oryza sativa Japonica Group cDNA clone:J033095G03, full insert seque | 0.00959147 | 0.05929 |
| Os.26626.1.S1_at | 2.84723 | BGIOSSBCE017474.1 // --- // bgi_ris // 0 // chr5:19569485-19570799(+) /// LOC_Os | 0.00254712 | 0.4744 |
| Os.50966.1.S1_at | 2.85851 | AK059817 // Oryza sativa Japonica Group cDNA clone:006-205-C02, full insert sequ | 0.000194167 | 0.3598 |
| Os.4378.4.S1_x_at | 2.85922 | AK067529 // Oryza sativa Japonica Group cDNA clone:J013108E24, full insert seque | 0.00023687 | 0.1464 |
| Os.23468.1.A1_at | 2.86421 | AK068378 // Oryza sativa Japonica Group cDNA clone:J013146K16, full insert seque | 0.0003741 | 0.3008 |
| Os.10197.1.S1_at | 2.87083 | AK071806 // Oryza sativa Japonica Group cDNA clone:J023111J10, full insert seque | 0.0024673 | 0.2176 |
| Os.50438.1.S1_at | 2.87297 | CT828313 // Oryza sativa (indica cultivar-group) cDNA clone:OSIGCPI012B19, full | 0.0033907 | 0.2677 |
| Os.4715.1.S1_at | 2.87328 | CT828477 // Oryza sativa (indica cultivar-group) cDNA clone:OSIGCRA110B14, full | 0.000665822 | 0.4066 |
| Os.20292.1.S1_at | 2.89214 | AK071680 // Oryza sativa Japonica Group cDNA clone:J023108B05, full insert seque | 0.0153037 | 0.452 |
| Os.10164.1.S1_at | 2.91349 | AK122172 // Oryza sativa Japonica Group cDNA clone:J033148H23, full insert seque | 0.00309687 | 0.04815 |
| Os.55395.1.S1_s_at | 2.91875 | BGIOSSFCE021419.1 // --- // bgi_ris // 9 // --- /// CT831422 // Oryza sativa (in | 0.000444294 | 0.1563 |
| OsAffx.15360.1.S1_at | 2.92557 | BGIOSSBCE019463.1 // --- // bgi_ris // 11 // --- /// BGIOSSBCE019464.1 // --- // | 0.00683059 | 0.1629 |
| Os.4653.1.S1_at | 2.93397 | EF520685 // Oryza sativa (japonica cultivar-group) Bowman-Birk type proteinase i | 0.0327634 | 0.33 |
| Os.19800.1.S1_at | 2.93752 | AY604047 // Oryza sativa (japonica cultivar-group) arginine decarboxylase (ADC1) | 0.00126317 | 0.07822 |
| OsAffx.27278.1.S1_at | 2.94732 | AK242393 // Oryza sativa Japonica Group cDNA, clone: J080064G22, full insert seq | 0.0039523 | 0.1012 |
| Os.52492.1.S1_at | 2.95284 | AK067494 // Oryza sativa Japonica Group cDNA clone:J013111P20, full insert seque | 0.00212433 | 0.03427 |
| Os.7920.1.S1_at | 2.95324 | AK243265 // Oryza sativa Japonica Group cDNA, clone: J100049E07, full insert seq | 0.00707865 | 0.06687 |
| Os.49787.1.S1_at | 2.95361 | AK111571 // Oryza sativa Japonica Group cDNA clone:J013071A13, full insert seque | 0.0285541 | 0.2781 |
| Os.50402.1.S1_at | 2.96351 | AK121064 // Oryza sativa Japonica Group cDNA clone:J023059C18, full insert seque | 0.0310174 | 0.5714 |
| Os.27906.1.S1_a_at | 2.96434 | AK071996 // Oryza sativa Japonica Group cDNA clone:J013091F23, full insert seque | 0.000951155 | 0.06438 |
| Os.5186.1.S1_at | 2.97076 | AK103609 // Oryza sativa Japonica Group cDNA clone:J033133I10, full insert seque | 0.00135403 | 0.2131 |
| Os.49502.1.S1_at | 2.97395 | CT830185 // Oryza sativa (indica cultivar-group) cDNA clone:OSIGCSN066P04, full | 0.0143651 | 0.1256 |
| Os.55394.1.S1_at | 2.97921 | LOC_Os03g52410.1 // expressed protein // rgap // 11 // --- /// NM_001057716 // O | 0.00209981 | 0.1297 |
| Os.27808.1.S1_s_at | 2.98836 | AK067697 // Oryza sativa Japonica Group cDNA clone:J013118D08, full insert seque | 0.00030602 | 0.3792 |
| Os.27807.1.S1_a_at | 2.99526 | AK063743 // Oryza sativa Japonica Group cDNA clone:001-120-F10, full insert sequ | 0.0212237 | 0.1609 |
| Os.6288.1.S1_at | 2.99629 | LOC_Os08g31850.1 // expressed protein // rgap // 11 // --- /// NM_001068331 // O | 0.00519445 | 0.2356 |
| Os.4378.2.S1_a_at | 2.99701 | AK067529 // Oryza sativa Japonica Group cDNA clone:J013108E24, full insert seque | 0.000272577 | 0.1604 |
| Os.10293.1.S1_at | 2.99846 | LOC_Os07g42510.1 // AP2 domain containing protein, expressed // rgap // 11 // -- | 0.020268 | 0.4988 |
| Os.5809.1.S1_at | 3.00082 | AK070289 // Oryza sativa Japonica Group cDNA clone:J023050F01, full insert seque | 0.00268187 | 0.03931 |
| Os.11417.1.S1_at | 3.01329 | AK072588 // Oryza sativa Japonica Group cDNA clone:J023132G16, full insert seque | 0.000374485 | 0.2324 |
| Os.20317.1.S1_at | 3.01631 | LOC_Os08g34900.1 // pectinesterase, putative, expressed // rgap // 11 // --- /// | 0.0220595 | 0.239 |
| Os.5622.1.S1_at | 3.03254 | AK066165 // Oryza sativa Japonica Group cDNA clone:J013052O14, full insert seque | 0.00507704 | 0.4753 |
| Os.28403.1.S1_a_at | 3.03569 | LOC_Os02g10390.1 // chlorophyll A-B binding protein, putative, expressed // rgap | 0.0283126 | 0.08655 |
| Os.13968.1.S1_a_at | 3.03847 | AF503583 // Oryza sativa (indica cultivar-group) early drought induced protein ( | 0.031155 | 0.3522 |
| Os.17887.1.S1_at | 3.04011 | AK102354 // Oryza sativa Japonica Group cDNA clone:J033091G14, full insert seque | 0.0297388 | 0.4749 |
| OsAffx.27278.1.S1_x_at | 3.04106 | AK242393 // Oryza sativa Japonica Group cDNA, clone: J080064G22, full insert seq | 0.0035878 | 0.1042 |
| Os.56314.1.S1_at | 3.04465 | LOC_Os03g19900.1 // AP2 domain containing protein, expressed // rgap // 10 // -- | 0.000221096 | 0.3371 |
| Os.52906.1.S1_at | 3.04483 | AK069841 // Oryza sativa Japonica Group cDNA clone:J023031G23, full insert seque | 0.000407858 | 0.1142 |
| Os.54782.1.S1_at | 3.05105 | AK106753 // Oryza sativa Japonica Group cDNA clone:002-115-D02, full insert sequ | 0.0234661 | 0.0725 |
| Os.15706.1.S1_a_at | 3.06228 | AK070670 // Oryza sativa Japonica Group cDNA clone:J023057H08, full insert seque | 0.00234047 | 0.05132 |
| Os.46832.1.S1_at | 3.08627 | LOC_Os10g28240.1 // calcium-transporting ATPase, plasma membrane-type, putative, | 0.00703336 | 0.1117 |
| Os.26768.1.S1_at | 3.09292 | LOC_Os03g58010.1 // acetyltransferase, GNAT family, putative, expressed // rgap | 0.00504123 | 0.3206 |
| Os.11867.2.S1_at | 3.10194 | AY594284 // Oryza sativa (japonica cultivar-group) cell death associated protein | 0.000128339 | 0.2158 |
| Os.24103.1.A1_at | 3.11503 | LOC_Os07g18990.1 // LTPL40 - Protease inhibitor/seed storage/LTP family protein | 0.0218713 | 0.3131 |
| Os.46050.1.A1_x_at | 3.1154 | AK106630 // Oryza sativa Japonica Group cDNA clone:002-113-D01, full insert sequ | 0.00286772 | 0.3076 |
| Os.12119.1.S1_at | 3.13543 | AK068115 // Oryza sativa Japonica Group cDNA clone:J013135B08, full insert seque | 0.000717915 | 0.1418 |
| Os.38235.2.S1_x_at | 3.13939 | LOC_Os04g52260.1 // LTPL124 - Protease inhibitor/seed storage/LTP family protein | 0.000635532 | 0.2909 |
| OsAffx.26001.1.S1_s_at | 3.14796 | LOC_Os10g22394.1 // retrotransposon protein, putative, unclassified // rgap // 1 | 0.000750814 | 0.04034 |
| Os.11354.1.S1_at | 3.15348 | AF443603 // Oryza sativa xyloglucan endotransglycosylase mRNA, complete cds. // | 0.0074525 | 0.3339 |
| Os.49503.1.S1_at | 3.15541 | LOC_Os03g27080.2 // calmodulin-binding transcription activator 2, putative, expr | 0.00366757 | 0.2082 |
| Os.51176.1.S1_at | 3.16655 | AK061298 // Oryza sativa Japonica Group cDNA clone:006-301-E04, full insert sequ | 0.0011156 | 0.4736 |
| Os.32602.2.S1_x_at | 3.17035 | AK103552 // Oryza sativa Japonica Group cDNA clone:J033132F15, full insert seque | 0.00688171 | 0.2892 |
| Os.55479.1.S1_at | 3.17679 | LOC_Os05g41490.1 // circadian clock coupling factor ZGT, putative, expressed // | 0.0188521 | 0.2077 |
| Os.37996.1.S1_at | 3.18775 | CT831693 // Oryza sativa (indica cultivar-group) cDNA clone:OSIGCSN016D14, full | 0.0202277 | 0.2315 |
| Os.55636.1.S1_at | 3.1948 | LOC_Os05g48010.1 // MYB family transcription factor, putative, expressed // rgap | 0.00112719 | 0.5197 |
| Os.46160.2.S1_x_at | 3.19961 | AK101170 // Oryza sativa Japonica Group cDNA clone:J033028K24, full insert seque | 0.0000549 | 0.2126 |
| Os.55392.1.S1_at | 3.20284 | LOC_Os02g57350.1 // expressed protein // rgap // 11 // --- /// NM_001055065 // O | 0.001649 | 0.2476 |
| Os.8149.1.S1_at | 3.20919 | AK111726 // Oryza sativa Japonica Group cDNA clone:J023036F23, full insert seque | 0.000380213 | 0.05798 |
| Os.12145.1.S1_at | 3.21038 | AK067007 // Oryza sativa Japonica Group cDNA clone:J013091D15, full insert seque | 0.0251845 | 0.09393 |
| Os.15942.1.S1_s_at | 3.21098 | AK061578 // Oryza sativa Japonica Group cDNA clone:006-311-H02, full insert sequ | 0.0480888 | 0.4017 |
| Os.2938.1.S1_x_at | 3.21329 | AK068088 // Oryza sativa Japonica Group cDNA clone:J013131C11, full insert seque | 0.00144974 | 0.2892 |
| Os.54238.1.S1_at | 3.22042 | AK102702 // Oryza sativa Japonica Group cDNA clone:J033103N20, full insert seque | 0.00493702 | 0.3025 |
| Os.4636.1.S2_at | 3.22045 | LOC_Os01g64170.1 // glycosyl hydrolases family 17, putative, expressed // rgap / | 0.00960099 | 0.315 |
| Os.37121.1.S1_at | 3.23508 | AK060548 // Oryza sativa Japonica Group cDNA clone:001-021-H04, full insert sequ | 0.000830134 | 0.02369 |
| OsAffx.7086.1.S1_at | 3.24974 | BGIOSSBCE031619.1 // --- // bgi_ris // 11 // --- /// BGIOSSFCE032090.1 // --- // | 0.0371299 | 0.4532 |
| Os.43600.1.S1_at | 3.25398 | LOC_Os01g10040.2 // cytochrome P450, putative, expressed // rgap // 11 // --- | 0.0181344 | 0.3927 |
| Os.7935.1.S1_at | 3.2545 | LOC_Os07g46852.1 // sex determination protein tasselseed-2, putative, expressed | 0.0183438 | 0.463 |
| OsAffx.26443.1.S1_s_at | 3.25927 | LOC_Os04g43440.1 // NB-ARC/LRR disease resistance protein, putative, expressed / | 0.00102809 | 0.385 |
| OsAffx.31501.1.S1_at | 3.2777 | BGIOSSFCE033455.1 // --- // bgi_ris // 11 // --- /// LOC_Os11g45850.1 // OsWRKY8 | 0.00151835 | 0.02999 |
| Os.18955.1.S1_at | 3.28743 | EU837248 // Oryza sativa Japonica Group clone KCS080F09 myb transcription factor | 0.00187622 | 0.04513 |
| Os.57182.1.S1_at | 3.29398 | NM_001068920 // Oryza sativa (japonica cultivar-group) Os08g0540900 (Os08g054090 | 0.000116934 | 0.2634 |
| Os.50243.1.S1_at | 3.29478 | LOC_Os06g39520.2 // myristoyl-acyl carrier protein thioesterase, chloroplast pre | 0.00187213 | 0.03965 |
| Os.53660.1.S1_at | 3.29753 | AK073812 // Oryza sativa Japonica Group cDNA clone:J033068P11, full insert seque | 0.0341033 | 0.2246 |
| OsAffx.31529.1.S1_at | 3.30094 | BGIOSSFCE033520.1 // --- // bgi_ris // 11 // --- /// CT835631 // Oryza sativa (i | 0.00308041 | 0.04018 |
| Os.3710.1.S1_at | 3.30843 | AB045120 // Oryza sativa Japonica Group EL5 mRNA, complete cds. // gb // 11 // - | 0.00712571 | 0.1288 |
| Os.8629.1.S1_x_at | 3.30954 | AF017360 // Oryza sativa lipid transfer protein LPT III mRNA, complete cds. // g | 0.00789766 | 0.3575 |
| Os.12937.1.S1_at | 3.3158 | LOC_Os04g51130.1 // expressed protein // rgap // 11 // --- /// NM_001060291 // O | 0.015999 | 0.2734 |
| Os.55850.1.S1_at | 3.3294 | AK108891 // Oryza sativa Japonica Group cDNA clone:002-152-E05, full insert sequ | 0.00322001 | 0.368 |
| Os.52902.1.S1_at | 3.3352 | AK069813 // Oryza sativa Japonica Group cDNA clone:J023030F23, full insert seque | 0.0147502 | 0.4613 |
| Os.40417.1.A1_at | 3.34453 | AK288078 // Oryza sativa Japonica Group cDNA, clone: J075164E22, full insert seq | 0.00269491 | 0.281 |
| Os.8104.1.S2_a_at | 3.3558 | AK068603 // Oryza sativa Japonica Group cDNA clone:J013157G11, full insert seque | 0.0246444 | 0.08568 |
| Os.6152.1.S1_at | 3.36063 | AK066089 // Oryza sativa Japonica Group cDNA clone:J013054O21, full insert seque | 0.00353313 | 0.304 |
| Os.12420.1.S1_at | 3.36084 | AK105533 // Oryza sativa Japonica Group cDNA clone:001-127-G12, full insert sequ | 0.0066297 | 0.3259 |
| Os.27764.1.S1_at | 3.37509 | LOC_Os05g38860.1 // expressed protein // rgap // 11 // --- | 0.0427184 | 0.2923 |
| Os.25276.1.S1_at | 3.37631 | AK107714 // Oryza sativa Japonica Group cDNA clone:002-132-E01, full insert sequ | 0.00110672 | 0.2477 |
| Os.15917.1.S1_at | 3.39398 | AB246888 // Oryza sativa Japonica Group OsNADPH-1 mRNA for NADPH oxidoreductase, | 0.00705795 | 0.4137 |
| Os.11326.1.S1_at | 3.40834 | AK069626 // Oryza sativa Japonica Group cDNA clone:J023024F13, full insert seque | 0.00462085 | 0.451 |
| Os.5999.1.S1_at | 3.40886 | LOC_Os12g28590.1 // ATPase 2, putative, expressed // rgap // 11 // --- /// NM_00 | 0.00100392 | 0.2503 |
| Os.7268.1.S1_at | 3.4338 | AK111542 // Oryza sativa Japonica Group cDNA clone:J013037N20, full insert seque | 0.00646611 | 0.3545 |
| Os.12167.1.S1_at | 3.44199 | AY587109 // Oryza sativa (japonica cultivar-group) dehydration-stress inducible | 0.00489975 | 0.1122 |
| Os.7935.1.S1_x_at | 3.44963 | LOC_Os07g46852.1 // sex determination protein tasselseed-2, putative, expressed | 0.0158729 | 0.3845 |
| Os.3388.2.S1_a_at | 3.45059 | AK111798 // Oryza sativa Japonica Group cDNA clone:J023105H16, full insert seque | 0.0251932 | 0.1161 |
| Os.30203.2.S1_at | 3.45823 | AK067145 // Oryza sativa Japonica Group cDNA clone:J013098M04, full insert seque | 0.00667667 | 0.04631 |
| Os.10839.1.S1_at | 3.47229 | LOC_Os03g16350.1 // DNA binding protein, putative, expressed // rgap // 11 // -- | 0.00161964 | 0.4252 |
| Os.37121.1.S2_at | 3.49907 | AK122042 // Oryza sativa Japonica Group cDNA clone:J033113J08, full insert seque | 0.000602999 | 0.0297 |
| OsAffx.15265.1.S1_at | 3.52321 | AK287701 // Oryza sativa Japonica Group cDNA, clone: J065130L06, full insert seq | 0.00275534 | 0.03566 |
| Os.19385.1.S1_at | 3.52424 | AK107020 // Oryza sativa Japonica Group cDNA clone:002-120-H09, full insert sequ | 0.0116623 | 0.272 |
| Os.11132.1.S1_s_at | 3.52936 | AK287608 // Oryza sativa Japonica Group cDNA, clone: J065058C07, full insert seq | 0.000106607 | 0.05224 |
| Os.53852.1.S1_at | 3.53564 | LOC_Os04g13210.1 // multidrug resistance-associated protein, putative, expressed | 0.00132018 | 0.2365 |
| Os.7338.1.S1_at | 3.5389 | LOC_Os07g28850.1 // retrotransposon protein, putative, unclassified, expressed / | 0.00261399 | 0.1358 |
| Os.4613.1.S1_at | 3.54635 | AF247162 // Oryza sativa alpha-expansin OsEXPA5 mRNA, complete cds. // gb // 11 | 0.015774 | 0.1262 |
| Os.3445.1.S1_at | 3.54901 | AF017366 // Oryza sativa metallothionein-like protein mRNA, complete cds. // gb | 0.0092505 | 0.6003 |
| Os.31858.1.S1_at | 3.56799 | AK103108 // Oryza sativa Japonica Group cDNA clone:J033118P18, full insert seque | 0.000242051 | 0.306 |
| Os.49419.1.S1_at | 3.57849 | AK065351 // Oryza sativa Japonica Group cDNA clone:J013020O22, full insert seque | 0.00245145 | 0.03614 |
| Os.11894.1.S1_at | 3.58242 | LOC_Os02g39330.1 // CHIT1 - Chitinase family protein precursor, expressed // rga | 0.00352072 | 0.2202 |
| Os.8570.3.S1_s_at | 3.58672 | AK063617 // Oryza sativa Japonica Group cDNA clone:001-118-D12, full insert sequ | 0.00481959 | 0.05043 |
| Os.2938.1.S1_at | 3.59684 | AK068088 // Oryza sativa Japonica Group cDNA clone:J013131C11, full insert seque | 0.00232071 | 0.3136 |
| Os.35013.1.S1_at | 3.61401 | CT828720 // Oryza sativa (indica cultivar-group) cDNA clone:OSIGCSN021L19, full | 0.0219532 | 0.09858 |
| Os.50985.1.S1_at | 3.61598 | LOC_Os05g36260.1 // soluble inorganic pyrophosphatase, putative, expressed // rg | 0.00146428 | 0.3142 |
| Os.17020.1.S1_x_at | 3.61966 | AK100858 // Oryza sativa Japonica Group cDNA clone:J023123M02, full insert seque | 0.0019365 | 0.3083 |
| Os.4782.1.S1_at | 3.63841 | CT832441 // Oryza sativa (indica cultivar-group) cDNA clone:OSIGCSN048K14, full | 0.000148243 | 0.3102 |
| Os.27713.1.A1_at | 3.64388 | AK120567 // Oryza sativa Japonica Group cDNA clone:J013134A09, full insert seque | 0.00116804 | 0.146 |
| Os.8545.1.S1_s_at | 3.66094 | CT829131 // Oryza sativa (indica cultivar-group) cDNA clone:OSIGCSN022I03, full | 0.000768025 | 0.3106 |
| Os.10097.1.S1_at | 3.66453 | AK121488 // Oryza sativa Japonica Group cDNA clone:J023150J09, full insert seque | 0.00230444 | 0.08278 |
| Os.12201.2.S1_at | 3.68408 | LOC_Os09g27820.1 // 1-aminocyclopropane-1-carboxylate oxidase protein, putative, | 0.0277737 | 0.3257 |
| Os.51714.1.S1_at | 3.68652 | AK073232 // Oryza sativa Japonica Group cDNA clone:J033021B15, full insert seque | 0.0136653 | 0.1382 |
| Os.15992.1.S1_at | 3.69434 | LOC_Os04g58190.1 // dof zinc finger domain containing protein, putative, express | 0.000814004 | 0.5107 |
| Os.28216.2.S1_s_at | 3.706 | AF022738 // Oryza sativa chlorophyll a-b binding protein mRNA, complete cds. // | 0.00718225 | 0.04919 |
| Os.7095.1.S1_at | 3.70724 | CT831123 // Oryza sativa (indica cultivar-group) cDNA clone:OSIGCSA053J09, full | 0.00168934 | 0.341 |
| Os.11040.1.S1_x_at | 3.71996 | AK061298 // Oryza sativa Japonica Group cDNA clone:006-301-E04, full insert sequ | 0.000837001 | 0.4479 |
| Os.9396.1.S1_at | 3.74396 | AK100981 // Oryza sativa Japonica Group cDNA clone:J023144M20, full insert seque | 0.00763864 | 0.1989 |
| Os.50825.1.S1_s_at | 3.75137 | AK100179 // Oryza sativa Japonica Group cDNA clone:J023029P10, full insert seque | 0.00262126 | 0.06979 |
| Os.8052.1.S1_at | 3.7545 | CT833245 // Oryza sativa (indica cultivar-group) cDNA clone:OSIGCRA118I06, full | 0.00116002 | 0.2147 |
| Os.10384.1.S1_at | 3.75576 | AK072292 // Oryza sativa Japonica Group cDNA clone:J023016C20, full insert seque | 0.0084984 | 0.2655 |
| Os.11065.1.A1_at | 3.78739 | LOC_Os03g19600.1 // retrotransposon protein, putative, unclassified, expressed / | 0.00139428 | 0.06485 |
| OsAffx.14311.1.S1_s_at | 3.81103 | AK288095 // Oryza sativa Japonica Group cDNA, clone: J075191E21, full insert seq | 0.0459077 | 0.3384 |
| Os.3397.1.S1_at | 3.81566 | AB001885 // Oryza sativa Japonica Group mRNA for zinc finger protein, complete c | 0.00407284 | 0.09602 |
| Os.15394.2.S1_at | 3.82722 | BGIOSSBCE001780.1 // --- // bgi_ris // 0 // chr1:17150608-17156364(+) /// LOC_Os | 0.0127632 | 0.05845 |
| Os.56080.1.S1_at | 3.83588 | CT828944 // Oryza sativa (indica cultivar-group) cDNA clone:OSIGCPI015D11, full | 0.0114862 | 0.2898 |
| Os.50411.2.S1_x_at | 3.84483 | AK066053 // Oryza sativa Japonica Group cDNA clone:J013052I15, full insert seque | 0.0021308 | 0.03495 |
| Os.55343.1.S1_at | 3.85452 | LOC_Os04g56030.1 // glycine-rich cell wall structural protein precursor, putativ | 0.000439113 | 0.3518 |
| Os.4770.1.S1_a_at | 3.85618 | AK106273 // Oryza sativa Japonica Group cDNA clone:002-100-G11, full insert sequ | 0.00156808 | 0.1016 |
| Os.15394.1.S1_at | 3.86533 | LOC_Os01g31360.2 // expressed protein // rgap // 11 // --- /// LOC_Os01g31360.3 | 0.0232098 | 0.07824 |
| Os.11029.1.S1_at | 3.87725 | LOC_Os03g16860.1 // DnaK family protein, putative, expressed // rgap // 11 // -- | 0.0202585 | 0.3706 |
| Os.9760.1.S1_at | 3.88291 | AK070347 // Oryza sativa Japonica Group cDNA clone:J023054E24, full insert seque | 0.00157336 | 0.3977 |
| Os.9749.1.S1_at | 3.88584 | LOC_Os08g44340.1 // monodehydroascorbate reductase, putative, expressed // rgap | 0.0000301 | 0.2146 |
| Os.49838.1.S1_at | 3.89372 | AK111866 // Oryza sativa Japonica Group cDNA clone:J033040N14, full insert seque | 0.000303076 | 0.3961 |
| Os.4385.1.S1_at | 3.89593 | AB028184 // Oryza sativa mRNA for OsNAC5 protein, complete cds. // gb // 11 // - | 0.00150036 | 0.1166 |
| Os.5049.1.S1_at | 3.89953 | AK069792 // Oryza sativa Japonica Group cDNA clone:J023031K22, full insert seque | 0.0067422 | 0.4545 |
| Os.11775.1.S2_at | 3.90184 | AK120741 // Oryza sativa Japonica Group cDNA clone:J023003P18, full insert seque | 0.000750139 | 0.3315 |
| Os.22831.1.S1_at | 3.90894 | LOC_Os03g50210.1 // DUF292 domain containing protein, expressed // rgap // 11 // | 0.0228147 | 0.4771 |
| Os.30200.1.S1_at | 3.91722 | LOC_Os11g47840.1 // OsRhmbd18 - Putative Rhomboid homologue, expressed // rgap / | 0.00158214 | 0.3027 |
| Os.26858.1.S1_at | 3.92136 | AK073697 // Oryza sativa Japonica Group cDNA clone:J033061P12, full insert seque | 0.0000666 | 0.2102 |
| Os.11798.1.S1_at | 3.93174 | AK101193 // Oryza sativa Japonica Group cDNA clone:J033030I03, full insert seque | 0.00604267 | 0.1676 |
| Os.37955.1.S1_a_at | 3.93243 | AK063085 // Oryza sativa Japonica Group cDNA clone:001-111-A07, full insert sequ | 0.00141225 | 0.03484 |
| Os.25687.1.S1_at | 3.93272 | AK068993 // Oryza sativa Japonica Group cDNA clone:J023001A06, full insert seque | 0.000849647 | 0.2816 |
| Os.52303.1.S1_x_at | 3.94803 | AK066260 // Oryza sativa Japonica Group cDNA clone:J013059H13, full insert seque | 0.000710657 | 0.02223 |
| Os.11983.1.S1_at | 3.95144 | AK073032 // Oryza sativa Japonica Group cDNA clone:J023146H06, full insert seque | 0.000295204 | 0.245 |
| Os.10024.1.S1_at | 3.95419 | AK070304 // Oryza sativa Japonica Group cDNA clone:J023049B02, full insert seque | 0.000556652 | 0.2782 |
| OsAffx.18589.1.S1_s_at | 3.95778 | AK241511 // Oryza sativa Japonica Group cDNA, clone: J065168H23, full insert seq | 0.0369167 | 0.3101 |
| Os.28124.1.S1_at | 3.97267 | LOC_Os10g28080.1 // glycosyl hydrolase, putative, expressed // rgap // 11 // --- | 0.0357308 | 0.2956 |
| Os.10654.1.S1_s_at | 3.97534 | AK062604 // Oryza sativa Japonica Group cDNA clone:001-105-A01, full insert sequ | 0.00141774 | 0.2618 |
| Os.15394.1.S1_s_at | 3.99635 | LOC_Os01g31360.2 // expressed protein // rgap // 11 // --- /// LOC_Os01g31360.4 | 0.0188681 | 0.07533 |
| Os.36152.1.S1_at | 4.0043 | LOC_Os01g63980.1 // ZOS1-17 - C2H2 zinc finger protein, expressed // rgap // 11 | 0.000106297 | 0.3448 |
| Os.20863.1.S1_at | 4.03831 | AK065438 // Oryza sativa Japonica Group cDNA clone:J013025A05, full insert seque | 0.000544375 | 0.2358 |
| OsAffx.24166.1.S1_at | 4.06628 | CT830591 // Oryza sativa (indica cultivar-group) cDNA clone:OSIGCRA124O19, full | 0.000643355 | 0.2353 |
| Os.33184.1.S1_at | 4.08413 | AK101454 // Oryza sativa Japonica Group cDNA clone:J033040A11, full insert seque | 0.000181378 | 0.4196 |
| Os.31521.1.S1_at | 4.10497 | AY341849 // Oryza sativa (japonica cultivar-group) WRKY8 mRNA, complete cds. // | 0.00287894 | 0.3704 |
| Os.14823.1.S1_s_at | 4.12116 | CT830663 // Oryza sativa (indica cultivar-group) cDNA clone:OSIGCRA128H09, full | 0.00103545 | 0.2593 |
| Os.56018.1.S1_at | 4.13727 | AK109131 // Oryza sativa Japonica Group cDNA clone:002-155-D10, full insert sequ | 0.00919429 | 0.2503 |
| Os.7322.1.S1_at | 4.1445 | BGIOSSFCE030074.1 // --- // bgi_ris // 10 // --- /// LOC_Os10g22730.1 // express | 0.00790223 | 0.05293 |
| Os.8088.1.S1_at | 4.14758 | AK070649 // Oryza sativa Japonica Group cDNA clone:J023062P07, full insert seque | 0.030342 | 0.3417 |
| Os.15394.2.S1_x_at | 4.149 | BGIOSSBCE001780.1 // --- // bgi_ris // 10 // --- /// LOC_Os01g31360.2 // express | 0.0113116 | 0.05794 |
| Os.46835.1.S1_at | 4.15354 | LOC_Os10g21860.1 // expressed protein // rgap // 11 // --- /// NM_001070972 // O | 0.00339081 | 0.06553 |
| Os.10933.1.S1_at | 4.19312 | D26536 // Oryza sativa Japonica Group mRNA for WSI18 protein induced by water st | 0.0086517 | 0.1573 |
| OsAffx.29276.1.S1_at | 4.19996 | AK288132 // Oryza sativa Japonica Group cDNA, clone: J080302F20, full insert seq | 0.000635993 | 0.06502 |
| Os.11657.1.S1_at | 4.20573 | LOC_Os01g47760.1 // OsGrx_I1 - glutaredoxin subgroup III, expressed // rgap // 1 | 0.0371493 | 0.2382 |
| Os.18085.1.S1_at | 4.2171 | LOC_Os04g44900.1 // lectin-like receptor kinase, putative, expressed // rgap // | 0.000321627 | 0.2744 |
| Os.37955.1.S2_at | 4.22106 | AK100539 // Oryza sativa Japonica Group cDNA clone:J023102F06, full insert seque | 0.00171464 | 0.03561 |
| Os.37548.1.S1_at | 4.26424 | LOC_Os05g34830.1 // No apical meristem protein, putative, expressed // rgap // 1 | 0.00237722 | 0.2794 |
| Os.18622.1.S1_at | 4.28378 | AK108145 // Oryza sativa Japonica Group cDNA clone:002-139-F03, full insert sequ | 0.00315749 | 0.3249 |
| Os.18053.1.S1_at | 4.30144 | BGIOSSBCE003107.1 // --- // bgi_ris // 9 // --- /// LOC_Os01g52340.1 // NB-ARC d | 0.0000696 | 0.326 |
| Os.52648.1.S1_at | 4.302 | AK068521 // Oryza sativa Japonica Group cDNA clone:J013155C12, full insert seque | 0.0000114 | 0.2786 |
| Os.6113.1.S1_at | 4.34018 | BGIOSSBCE024600.1 // --- // bgi_ris // 9 // --- /// LOC_Os08g04230.1 // cysteine | 0.0062385 | 0.284 |
| Os.5644.1.S1_at | 4.35505 | LOC_Os10g33540.1 // alcohol oxidase, putative, expressed // rgap // 11 // --- | 0.0236884 | 0.3307 |
| OsAffx.5948.1.S1_at | 4.36373 | LOC_Os08g27170.1 // calmodulin binding protein, putative // rgap // 11 // --- // | 0.019829 | 0.3389 |
| Os.12295.1.S1_at | 4.41937 | AK059990 // Oryza sativa Japonica Group cDNA clone:006-301-A12, full insert sequ | 0.0151714 | 0.1378 |
| Os.15633.1.S1_at | 4.43895 | AK071225 // Oryza sativa Japonica Group cDNA clone:J023087K13, full insert seque | 0.00421293 | 0.4226 |
| Os.12165.1.S1_at | 4.44055 | AK289238 // Oryza sativa Japonica Group cDNA, clone: J100074M03, full insert seq | 0.00763234 | 0.07149 |
| Os.5031.1.S1_at | 4.44599 | AB127580 // Oryza sativa Japonica Group RSOsPR10 mRNA for root specific pathogen | 0.0000657 | 0.2369 |
| Os.9739.1.S1_at | 4.45093 | AK103013 // Oryza sativa Japonica Group cDNA clone:J033116L09, full insert seque | 0.00156586 | 0.352 |
| Os.54932.1.S1_at | 4.45174 | LOC_Os12g07030.1 // expressed protein // rgap // 11 // --- /// NM_001072784 // O | 0.0055843 | 0.2048 |
| Os.57298.1.S1_at | 4.46453 | AK111259 // Oryza sativa Japonica Group cDNA clone:002-180-E11, full insert sequ | 0.000902655 | 0.03427 |
| Os.40045.1.S1_at | 4.48572 | BGIOSSBCE033746.1 // --- // bgi_ris // 0 // chr12:27076188-27078549(+) /// BGIOS | 0.00283499 | 0.4229 |
| Os.39872.1.A1_s_at | 4.52046 | AK242227 // Oryza sativa Japonica Group cDNA, clone: J075175B09, full insert seq | 0.000521252 | 0.2914 |
| Os.53148.1.S1_at | 4.53154 | AK071016 // Oryza sativa Japonica Group cDNA clone:J023081E04, full insert seque | 0.00283567 | 0.2786 |
| Os.7870.1.S1_at | 4.54572 | LOC_Os02g39850.1 // transferase family protein, putative, expressed // rgap // 1 | 0.000531539 | 0.2401 |
| OsAffx.30939.2.S1_s_at | 4.5467 | AK242746 // Oryza sativa Japonica Group cDNA, clone: J090050D06, full insert seq | 0.0142258 | 0.2739 |
| Os.37565.1.S1_x_at | 4.55662 | AK063697 // Oryza sativa Japonica Group cDNA clone:001-119-G04, full insert sequ | 0.00171823 | 0.06924 |
| Os.34782.1.S1_at | 4.57307 | CT832545 // Oryza sativa (indica cultivar-group) cDNA clone:OSIGCPI021O13, full | 0.0247283 | 0.343 |
| OsAffx.27557.1.S1_at | 4.58377 | BGIOSSBCE008088.1 // --- // bgi_ris // 0 // chr2:32325339-32326056(-) /// BGIOSS | 0.00895577 | 0.2885 |
| Os.46160.2.S1_at | 4.59049 | AK101170 // Oryza sativa Japonica Group cDNA clone:J033028K24, full insert seque | 0.00150587 | 0.2838 |
| Os.50342.1.S1_at | 4.59137 | AK120799 // Oryza sativa Japonica Group cDNA clone:J023012H18, full insert seque | 0.00302889 | 0.2871 |
| Os.57193.1.S1_at | 4.60011 | NM_001052669 // Oryza sativa (japonica cultivar-group) Os02g0188400 (Os02g018840 | 0.00845568 | 0.312 |
| Os.51172.1.S1_x_at | 4.61497 | LOC_Os06g51050.1 // CHIT7 - Chitinase family protein precursor, expressed // rga | 0.00321254 | 0.215 |
| Os.10901.1.S1_a_at | 4.62413 | AK104073 // Oryza sativa Japonica Group cDNA clone:001-027-C12, full insert sequ | 0.0101832 | 0.06147 |
| Os.9945.1.S1_at | 4.64331 | AK073044 // Oryza sativa Japonica Group cDNA clone:J033001E11, full insert seque | 0.0203864 | 0.2187 |
| Os.54864.1.S1_at | 4.65966 | NM_001063607 // Oryza sativa (japonica cultivar-group) Os06g0200700 (Os06g020070 | 0.0417826 | 0.3893 |
| Os.11471.1.S1_at | 4.67015 | AK121265 // Oryza sativa Japonica Group cDNA clone:J023105D11, full insert seque | 0.0189904 | 0.263 |
| Os.11237.1.S1_at | 4.67992 | LOC_Os03g18560.1 // DUF538 domain containing protein, putative, expressed // rga | 0.00955642 | 0.314 |
| Os.15706.1.S1_at | 4.74186 | AK070670 // Oryza sativa Japonica Group cDNA clone:J023057H08, full insert seque | 0.0129242 | 0.09856 |
| Os.23054.1.S1_at | 4.74708 | AK105639 // Oryza sativa Japonica Group cDNA clone:001-200-E04, full insert sequ | 0.000129156 | 0.2648 |
| Os.15060.1.S1_at | 4.77208 | CT835732 // Oryza sativa (indica cultivar-group) cDNA clone:OSIGCFA218B02, full | 0.00185342 | 0.03246 |
| OsAffx.27605.1.S1_s_at | 4.77583 | LOC_Os06g13190.1 // expressed protein // rgap // 11 // --- | 0.00112978 | 0.2616 |
| OsAffx.31405.1.S1_at | 4.78191 | AK240898 // Oryza sativa Japonica Group cDNA, clone: J065032M10, full insert seq | 0.00010721 | 0.01582 |
| Os.5384.1.S1_at | 4.78783 | AK062356 // Oryza sativa Japonica Group cDNA clone:001-101-G11, full insert sequ | 0.0127536 | 0.2658 |
| Os.9292.1.S1_at | 4.90543 | AK111536 // Oryza sativa Japonica Group cDNA clone:J013031I11, full insert seque | 0.000391564 | 0.04966 |
| Os.37955.1.S1_at | 4.91202 | AK063604 // Oryza sativa Japonica Group cDNA clone:001-118-C07, full insert sequ | 0.00482121 | 0.04112 |
| Os.47761.1.S1_at | 4.92175 | LOC_Os03g13300.1 // glutamate decarboxylase, putative, expressed // rgap // 11 / | 0.0132695 | 0.2721 |
| Os.37621.3.S1_at | 4.92257 | BGIOSSBCE000106.1 // --- // bgi_ris // 11 // --- /// BGIOSSFCE000106.1 // --- // | 0.0120081 | 0.2963 |
| Os.7941.2.S1_s_at | 4.94587 | AK121563 // Oryza sativa Japonica Group cDNA clone:J033034J10, full insert seque | 0.002108 | 0.03808 |
| Os.11296.1.S1_a_at | 5.0098 | AK065344 // Oryza sativa Japonica Group cDNA clone:J013007H05, full insert seque | 0.0017911 | 0.0965 |
| Os.27738.1.S1_at | 5.0182 | AK069726 // Oryza sativa Japonica Group cDNA clone:J023030M13, full insert seque | 0.000110661 | 0.3008 |
| OsAffx.14605.1.S1_s_at | 5.02769 | CT832801 // Oryza sativa (indica cultivar-group) cDNA clone:OSIGCSA009K06, full | 0.000572939 | 0.2984 |
| Os.33800.1.S1_at | 5.06523 | AK074006 // Oryza sativa Japonica Group cDNA clone:J033077D15, full insert seque | 0.00193478 | 0.08506 |
| Os.52303.1.S1_at | 5.08424 | AK066260 // Oryza sativa Japonica Group cDNA clone:J013059H13, full insert seque | 0.000868667 | 0.04341 |
| Os.55597.1.S1_at | 5.10631 | AK108389 // Oryza sativa Japonica Group cDNA clone:002-142-F03, full insert sequ | 0.0435288 | 0.335 |
| Os.51798.1.S1_at | 5.11082 | AK063737 // Oryza sativa Japonica Group cDNA clone:001-120-F04, full insert sequ | 0.00462057 | 0.04513 |
| Os.37890.1.S1_at | 5.15685 | LOC_Os11g24070.1 // LTPL10 - Protease inhibitor/seed storage/LTP family protein | 0.00149131 | 0.2596 |
| Os.20364.1.S1_at | 5.22089 | AB051864 // Oryza sativa OsAMT2;1 mRNA for ammonium transporter, complete cds. / | 0.00630576 | 0.2201 |
| Os.46633.1.A1_at | 5.25958 | CT837931 // Oryza sativa (indica cultivar-group) cDNA clone:OSIGCSA058D18, full | 0.0257532 | 0.1161 |
| Os.50449.1.S1_at | 5.26618 | AK100415 // Oryza sativa Japonica Group cDNA clone:J023087M18, full insert seque | 0.00878768 | 0.2899 |
| Os.10619.1.S2_at | 5.27693 | AK100082 // Oryza sativa Japonica Group cDNA clone:J023002E24, full insert seque | 0.000185555 | 0.3638 |
| Os.9585.1.S1_s_at | 5.30949 | AB060552 // Oryza sativa Japonica Group OsCAM-2 mRNA for Calmodulin-2, complete | 0.0275936 | 0.3981 |
| Os.39063.1.A1_s_at | 5.31895 | AK100539 // Oryza sativa Japonica Group cDNA clone:J023102F06, full insert seque | 0.00105061 | 0.0297 |
| Os.51817.1.S1_at | 5.32269 | AK063851 // Oryza sativa Japonica Group cDNA clone:001-122-C12, full insert sequ | 0.00208823 | 0.03614 |
| Os.51929.1.S1_at | 5.33053 | LOC_Os10g31330.1 // retrotransposon protein, putative, unclassified, expressed / | 0.0210253 | 0.5227 |
| Os.9677.2.S1_x_at | 5.40407 | AK106438 // Oryza sativa Japonica Group cDNA clone:002-103-D06, full insert sequ | 0.00412502 | 0.3063 |
| Os.1193.1.S1_at | 5.40409 | AF001505 // Oryza sativa putative ammonium transporter OsAMT1p (OsAMT1) mRNA, co | 0.00838724 | 0.263 |
| Os.50411.1.S1_at | 5.42752 | AK121102 // Oryza sativa Japonica Group cDNA clone:J023069N11, full insert seque | 0.000294182 | 0.01738 |
| Os.9067.1.S1_at | 5.43442 | AK121733 // Oryza sativa Japonica Group cDNA clone:J033083C10, full insert seque | 0.023981 | 0.3058 |
| Os.17479.1.S1_at | 5.43971 | AK106178 // Oryza sativa Japonica Group cDNA clone:001-208-C10, full insert sequ | 0.00668628 | 0.2749 |
| Os.12498.2.S1_at | 5.45367 | LOC_Os01g72360.1 // expressed protein // rgap // 11 // --- /// NM_001051958 // O | 0.0148456 | 0.2659 |
| Os.51959.1.S2_at | 5.4561 | AK101902 // Oryza sativa Japonica Group cDNA clone:J033071C04, full insert seque | 0.00309546 | 0.3427 |
| Os.12652.1.S1_at | 5.56192 | CT833337 // Oryza sativa (indica cultivar-group) cDNA clone:OSIGCRA205A07, full | 0.000206678 | 0.03449 |
| Os.55550.1.S1_at | 5.57697 | LOC_Os02g47370.1 // transcription factor TF2, putative, expressed // rgap // 11 | 0.00303095 | 0.2564 |
| Os.46956.1.S1_at | 5.5849 | LOC_Os01g50940.1 // helix-loop-helix DNA-binding domain containing protein, expr | 0.00492523 | 0.273 |
| Os.54463.1.S1_at | 5.66864 | LOC_Os05g48680.1 // expressed protein // rgap // 11 // --- /// NM_001062844 // O | 0.0272092 | 0.2701 |
| OsAffx.14605.1.S1_at | 5.68296 | CT832801 // Oryza sativa (indica cultivar-group) cDNA clone:OSIGCSA009K06, full | 0.000642376 | 0.3078 |
| Os.51479.1.S1_at | 5.68557 | AK062914 // Oryza sativa Japonica Group cDNA clone:001-108-G12, full insert sequ | 0.00175284 | 0.3643 |
| Os.19519.1.S1_s_at | 5.68655 | AK289238 // Oryza sativa Japonica Group cDNA, clone: J100074M03, full insert seq | 0.00590506 | 0.06629 |
| Os.10908.1.S1_a_at | 5.73642 | AK073487 // Oryza sativa Japonica Group cDNA clone:J033041J03, full insert seque | 0.00642864 | 0.6077 |
| Os.15633.1.S2_at | 5.83734 | AK071225 // Oryza sativa Japonica Group cDNA clone:J023087K13, full insert seque | 0.00949099 | 0.5963 |
| Os.36901.1.S1_at | 5.87272 | LOC_Os01g58150.1 // expressed protein // rgap // 11 // --- /// NM_001051037 // O | 0.0258128 | 0.2861 |
| Os.12452.1.S1_s_at | 5.89409 | AK073487 // Oryza sativa Japonica Group cDNA clone:J033041J03, full insert seque | 0.00744487 | 0.5592 |
| Os.9022.1.S1_at | 5.89911 | LOC_Os09g15670.1 // protein phosphatase 2C, putative, expressed // rgap // 11 // | 0.0106432 | 0.08145 |
| Os.55293.1.S1_at | 5.95145 | LOC_Os06g05510.1 // expressed protein // rgap // 11 // --- /// NM_001063339 // O | 0.0100011 | 0.2994 |
| Os.10510.1.S1_at | 5.96466 | AK068438 // Oryza sativa Japonica Group cDNA clone:J013154K17, full insert seque | 0.00708114 | 0.2284 |
| Os.37067.1.S1_at | 5.99504 | AK061038 // Oryza sativa Japonica Group cDNA clone:006-205-C01, full insert sequ | 0.000378389 | 0.09412 |
| Os.55682.1.S1_at | 6.0028 | LOC_Os03g22820.1 // expressed protein // rgap // 11 // --- /// NM_001056654 // O | 0.00772732 | 0.266 |
| Os.17601.1.S1_at | 6.15516 | AK120803 // Oryza sativa Japonica Group cDNA clone:J023013B14, full insert seque | 0.00115352 | 0.3097 |
| OsAffx.7025.1.S1_at | 6.22176 | LOC_Os11g06770.1 // ethylene-responsive transcription factor ERF110, putative, e | 0.00751279 | 0.1771 |
| Os.2227.1.S1_at | 6.24359 | AF090872 // Oryza sativa 33 kDa secretory protein mRNA, complete cds. // gb // 1 | 0.0115045 | 0.3616 |
| Os.54232.1.S1_at | 6.26486 | AK102658 // Oryza sativa Japonica Group cDNA clone:J033101H19, full insert seque | 0.00255166 | 0.2346 |
| Os.51226.1.S1_at | 6.34879 | LOC_Os03g52910.1 // uncharacterized UPF0114 domain containing protein, expressed | 0.00538649 | 0.1993 |
| Os.10884.1.S1_at | 6.38553 | AK070731 // Oryza sativa Japonica Group cDNA clone:J023061A01, full insert seque | 0.000183756 | 0.2773 |
| Os.10235.1.S1_at | 6.39052 | LOC_Os07g48570.1 // expressed protein // rgap // 11 // --- /// NM_001067208 // O | 0.0038748 | 0.06938 |
| Os.37243.2.S1_x_at | 6.45562 | NM_001050593 // Oryza sativa (japonica cultivar-group) Os01g0714600 (Os01g071460 | 0.0331051 | 0.239 |
| Os.53981.1.S1_at | 6.61275 | LOC_Os11g45890.1 // expressed protein // rgap // 11 // --- /// NM_001075008 // O | 0.000512724 | 0.0239 |
| Os.11290.1.S1_at | 6.65989 | LOC_Os06g44170.1 // NAD dependent epimerase/dehydratase family protein, putative | 0.00137801 | 0.3188 |
| Os.6092.1.S1_at | 6.76204 | AB120515 // Oryza sativa Japonica Group OsTPP1 mRNA for trehalose-6-phosphate ph | 0.000115795 | 0.1329 |
| Os.37890.1.S1_s_at | 6.78651 | LOC_Os11g24070.1 // LTPL10 - Protease inhibitor/seed storage/LTP family protein | 0.0044039 | 0.2854 |
| Os.9145.1.S1_at | 6.8408 | AK070360 // Oryza sativa Japonica Group cDNA clone:J023048G01, full insert seque | 0.000220576 | 0.2616 |
| Os.55034.1.S1_at | 6.90473 | AK107351 // Oryza sativa Japonica Group cDNA clone:002-126-H06, full insert sequ | 0.000158617 | 0.01578 |
| Os.7941.2.S1_a_at | 6.9185 | AK121563 // Oryza sativa Japonica Group cDNA clone:J033034J10, full insert seque | 0.000956931 | 0.02613 |
| OsAffx.32023.1.S1_at | 7.04165 | LOC_Os03g42510.1 // transposon protein, putative, CACTA, En/Spm sub-class // rga | 0.000492112 | 0.1439 |
| Os.10126.1.S1_at | 7.07904 | CT828454 // Oryza sativa (indica cultivar-group) cDNA clone:OSIGCSN010A13, full | 0.0059535 | 0.0446 |
| Os.15688.1.S1_at | 7.09823 | AK067865 // Oryza sativa Japonica Group cDNA clone:J013124K14, full insert seque | 0.000389347 | 0.1001 |
| Os.37565.1.S1_at | 7.31692 | AK063697 // Oryza sativa Japonica Group cDNA clone:001-119-G04, full insert sequ | 0.00194555 | 0.05283 |
| Os.16401.1.S1_at | 7.33621 | LOC_Os03g21380.1 // OsCML27 - Calmodulin-related calcium sensor protein, express | 0.00277791 | 0.2816 |
| OsAffx.26001.1.S1_at | 7.39481 | LOC_Os10g22394.1 // retrotransposon protein, putative, unclassified // rgap // 1 | 0.00932853 | 0.0893 |
| Os.10734.1.S1_at | 7.5114 | AK101544 // Oryza sativa Japonica Group cDNA clone:J033048K18, full insert seque | 0.000342186 | 0.02059 |
| Os.29987.2.S1_at | 7.58084 | LOC_Os05g50610.2 // OsWRKY8v2 - Superfamily of TFs having WRKY and zinc finger d | 0.0155544 | 0.3012 |
| Os.49528.2.S1_at | 7.60143 | LOC_Os04g44910.1 // receptor like protein kinase, putative, expressed // rgap // | 0.0010375 | 0.2941 |
| Os.52601.1.S1_at | 7.61739 | AK103221 // Oryza sativa Japonica Group cDNA clone:J033122P06, full insert seque | 0.00128205 | 0.02857 |
| Os.55306.1.S1_at | 7.65739 | AK107839 // Oryza sativa Japonica Group cDNA clone:002-133-H08, full insert sequ | 0.00512074 | 0.08472 |
| Os.5353.1.S1_at | 7.74508 | LOC_Os10g22630.1 // expressed protein // rgap // 11 // --- | 0.00477295 | 0.0433 |
| Os.27097.1.A1_x_at | 7.80013 | AK064444 // Oryza sativa Japonica Group cDNA clone:002-110-E09, full insert sequ | 0.000264572 | 0.01787 |
| Os.7228.1.S1_at | 7.94265 | LOC_Os02g51970.1 // phosphate-induced protein 1 conserved region domain containi | 0.0011617 | 0.1909 |
| Os.11196.1.S1_at | 7.99759 | CT836008 // Oryza sativa (indica cultivar-group) cDNA clone:OSIGCSN022F03, full | 0.000871194 | 0.03787 |
| Os.53105.1.S1_at | 8.02979 | AK070844 // Oryza sativa Japonica Group cDNA clone:J023073J15, full insert seque | 0.00284777 | 0.2471 |
| Os.27387.1.S1_x_at | 8.04814 | LOC_Os01g17396.1 // expressed protein // rgap // 11 // --- /// LOC_Os01g17396.2 | 0.00596641 | 0.1205 |
| Os.10930.1.S1_at | 8.0715 | LOC_Os02g41670.1 // phenylalanine ammonia-lyase, putative, expressed // rgap // | 0.0013267 | 0.1978 |
| OsAffx.24153.1.S1_s_at | 8.0801 | LOC_Os02g07690.1 // VQ domain containing protein, putative, expressed // rgap // | 0.00236385 | 0.2405 |
| Os.54928.1.S1_at | 8.13377 | CT831506 // Oryza sativa (indica cultivar-group) cDNA clone:OSIGCSN065M06, full | 0.000534961 | 0.3156 |
| Os.51741.1.S1_at | 8.25753 | CT829837 // Oryza sativa (indica cultivar-group) cDNA clone:OSIGCSA003N17, full | 0.000569968 | 0.2629 |
| Os.4651.1.S1_at | 8.28265 | LOC_Os07g48710.1 // VQ domain containing protein, putative, expressed // rgap // | 0.0000202 | 0.2501 |
| Os.37565.2.S1_at | 8.3043 | LOC_Os05g25770.1 // OsWRKY45 - Superfamily of TFs having WRKY and zinc finger do | 0.00807506 | 0.1237 |
| Os.37243.1.S1_at | 8.31976 | AK059855 // Oryza sativa Japonica Group cDNA clone:006-206-H11, full insert sequ | 0.0154127 | 0.2031 |
| Os.30657.1.S1_at | 8.32056 | AY341846 // Oryza sativa (japonica cultivar-group) WRKY5 mRNA, complete cds. // | 0.0000264 | 0.272 |
| Os.50015.1.S1_at | 8.34091 | LOC_Os06g44010.1 // OsWRKY28 - Superfamily of TFs having WRKY and zinc finger do | 0.0152843 | 0.139 |
| Os.17182.1.S1_at | 8.38533 | AK072119 // Oryza sativa Japonica Group cDNA clone:J013123D02, full insert seque | 0.00144549 | 0.3015 |
| Os.4683.3.S1_at | 8.38927 | AB040742 // Oryza sativa RRJ2 premature mRNA for pyruvate decarboxylase, partial | 0.0391519 | 0.2648 |
| Os.169.1.S1_at | 8.46309 | AK073800 // Oryza sativa Japonica Group cDNA clone:J033067D20, full insert seque | 0.0091294 | 0.2712 |
| Os.51606.1.S1_at | 8.47079 | AK063156 // Oryza sativa Japonica Group cDNA clone:001-111-H12, full insert sequ | 0.000000353 | 0.2431 |
| Os.18922.2.S1_at | 8.516 | CT829966 // Oryza sativa (indica cultivar-group) cDNA clone:OSIGCRA111C06, full | 0.0162735 | 0.2793 |
| Os.31778.2.S1_at | 8.59462 | LOC_Os01g61610.1 // flavonol synthase/flavanone 3-hydroxylase, putative, express | 0.000477156 | 0.08658 |
| Os.8545.1.S1_at | 8.60966 | CT829131 // Oryza sativa (indica cultivar-group) cDNA clone:OSIGCSN022I03, full | 0.0021209 | 0.2853 |
| Os.12012.1.S1_at | 8.62632 | LOC_Os10g25290.1 // ZIM domain containing protein, putative, expressed // rgap / | 0.0104131 | 0.2407 |
| Os.27387.1.S1_at | 8.85671 | LOC_Os01g17396.1 // expressed protein // rgap // 11 // --- /// LOC_Os01g17396.2 | 0.00661178 | 0.1207 |
| Os.27097.1.A1_at | 9.11412 | AK064444 // Oryza sativa Japonica Group cDNA clone:002-110-E09, full insert sequ | 0.00011097 | 0.02613 |
| Os.51063.1.S1_at | 9.17355 | AK070458 // Oryza sativa Japonica Group cDNA clone:J023048E05, full insert seque | 0.0000651 | 0.07585 |
| OsAffx.18575.1.S1_x_at | 9.21699 | LOC_Os10g39140.1 // flavonol synthase/flavanone 3-hydroxylase, putative, express | 0.00624685 | 0.1807 |
| Os.18443.1.S1_at | 9.44189 | AK073119 // Oryza sativa Japonica Group cDNA clone:J033001C17, full insert seque | 0.000556271 | 0.02124 |
| Os.46902.1.S1_at | 9.50649 | AJ302060 // Oryza sativa mRNA for glycine rich RNA binding protein (grp5 gene). | 0.0153315 | 0.06112 |
| Os.14616.1.S1_at | 9.53328 | AK241194 // Oryza sativa Japonica Group cDNA, clone: J065121D09, full insert seq | 0.0103701 | 0.2595 |
| Os.52147.1.S1_at | 9.53833 | AK065205 // Oryza sativa Japonica Group cDNA clone:J013002F11, full insert seque | 0.0167479 | 0.3284 |
| Os.11812.1.S1_at | 9.70876 | LOC_Os02g48770.1 // SAM dependent carboxyl methyltransferase, putative, expresse | 0.00137131 | 0.2165 |
| Os.50399.1.S1_at | 9.82461 | AK121050 // Oryza sativa Japonica Group cDNA clone:J023055G07, full insert seque | 0.0000341 | 0.2201 |
| Os.54944.1.S1_at | 10.24 | LOC_Os02g52670.1 // AP2 domain containing protein, expressed // rgap // 11 // -- | 0.000304847 | 0.2284 |
| Os.15688.2.S1_x_at | 10.4765 | AK067126 // Oryza sativa Japonica Group cDNA clone:J013096O03, full insert seque | 0.000766248 | 0.1423 |
| OsAffx.27542.1.S1_s_at | 10.4846 | BGIOSSFCE019645.1 // --- // bgi_ris // 11 // --- /// LOC_Os06g09990.1 // hypothe | 0.00540571 | 0.2345 |
| Os.8961.1.S1_s_at | 10.5944 | CT837881 // Oryza sativa (indica cultivar-group) cDNA clone:OSIGCRA124O14, full | 0.000221385 | 0.2467 |
| Os.23207.1.S1_at | 10.6977 | LOC_Os08g04340.1 // plastocyanin-like domain containing protein, putative, expre | 0.00129543 | 0.2549 |
| Os.55408.1.S1_at | 10.781 | LOC_Os04g52750.1 // expressed protein // rgap // 11 // --- /// NM_001060426 // O | 0.00471485 | 0.2259 |
| Os.26411.1.A1_at | 11.2037 | AY224427 // Oryza sativa (japonica cultivar-group) isolate 20474 receptor kinase | 0.00188609 | 0.2502 |
| Os.49619.1.S1_at | 11.4759 | CT830663 // Oryza sativa (indica cultivar-group) cDNA clone:OSIGCRA128H09, full | 0.000299107 | 0.2507 |
| Os.10356.1.S1_at | 11.5146 | AK241472 // Oryza sativa Japonica Group cDNA, clone: J065165F04, full insert seq | 0.00877703 | 0.2044 |
| Os.10860.2.S1_at | 12.3177 | AK069437 // Oryza sativa Japonica Group cDNA clone:J023023O07, full insert seque | 0.00240482 | 0.2804 |
| Os.11506.1.S1_at | 12.351 | AK101888 // Oryza sativa Japonica Group cDNA clone:J033070M12, full insert seque | 0.0000264 | 0.004478 |
| Os.7213.1.S1_at | 12.4082 | LOC_Os03g26229.1 // 40S ribosomal protein S9-1, putative, expressed // rgap // 1 | 0.000874202 | 0.02613 |
| OsAffx.21616.1.S1_s_at | 12.4594 | LOC_Os01g54890.1 // ethylene-responsive transcription factor 2, putative, expres | 0.00655231 | 0.3226 |
| OsAffx.28164.1.S1_at | 12.7463 | AK064918 // Oryza sativa Japonica Group cDNA clone:J013000M23, full insert seque | 0.0457368 | 0.1114 |
| Os.28514.1.S1_at | 12.7694 | CT833192 // Oryza sativa (indica cultivar-group) cDNA clone:OSIGCRA111J19, full | 0.0000888 | 0.1622 |
| Os.12063.1.S1_at | 13.3465 | CT829812 // Oryza sativa (indica cultivar-group) cDNA clone:OSIGCSN040P20, full | 0.000304696 | 0.2958 |
| OsAffx.11050.2.S1_x_at | 13.3571 | AK105509 // Oryza sativa Japonica Group cDNA clone:001-127-E04, full insert sequ | 0.000785459 | 0.1935 |
| Os.6961.1.S1_a_at | 14.407 | AK065366 // Oryza sativa Japonica Group cDNA clone:J013020D06, full insert seque | 0.000151634 | 0.223 |
| Os.170.1.S1_at | 14.5096 | AB003195 // Oryza sativa Japonica Group mRNA for basic class III chitinase OsChi | 0.000540925 | 0.3052 |
| Os.9554.1.S1_at | 14.5561 | AK101227 // Oryza sativa Japonica Group cDNA clone:J033031F14, full insert seque | 0.00132829 | 0.3487 |
| Os.31778.2.S1_x_at | 15.4722 | LOC_Os01g61610.1 // flavonol synthase/flavanone 3-hydroxylase, putative, express | 0.000902835 | 0.0864 |
| Os.12048.1.S1_at | 15.5965 | AK065217 // Oryza sativa Japonica Group cDNA clone:J013002G10, full insert seque | 0.00103872 | 0.3102 |
| Os.53889.1.S1_at | 16.424 | AK100635 // Oryza sativa Japonica Group cDNA clone:J023110C18, full insert seque | 0.000119253 | 0.01582 |
| Os.53617.1.S1_at | 16.5754 | AK101155 // Oryza sativa Japonica Group cDNA clone:J033027E14, full insert seque | 0.040139 | 0.3247 |
| Os.39031.1.S1_at | 17.6211 | BGIOSSFCE019645.1 // --- // bgi_ris // 0 // chr6:5096035-5096381(-) /// LOC_Os06 | 0.00315079 | 0.2662 |
| Os.609.3.S1_a_at | 17.6504 | AK066830 // Oryza sativa Japonica Group cDNA clone:J013075I22, full insert seque | 0.0000083 | 0.238 |
| Os.26932.1.S1_at | 17.7093 | LOC_Os07g37320.1 // transporter family protein, putative, expressed // rgap // 1 | 0.000165683 | 0.2443 |
| Os.22000.1.S1_at | 17.9162 | EF122477 // Oryza sativa (japonica cultivar-group) clone KCG045D08 chitinase mRN | 0.000591416 | 0.288 |
| Os.46849.1.S1_at | 18.0658 | LOC_Os10g25230.1 // ZIM domain containing protein, putative, expressed // rgap / | 0.00196742 | 0.21 |
| Os.32890.1.S1_at | 18.5565 | LOC_Os11g47600.1 // glycosyl hydrolase, putative, expressed // rgap // 11 // --- | 0.0090701 | 0.2698 |
| Os.56950.1.S1_at | 18.7826 | LOC_Os05g07120.1 // basic helix-loop-helix, putative, expressed // rgap // 11 // | 0.00385789 | 0.2257 |
| Os.31778.1.S1_x_at | 19.606 | LOC_Os01g61610.1 // flavonol synthase/flavanone 3-hydroxylase, putative, express | 0.000456105 | 0.09382 |
| Os.32602.1.S1_at | 20.6864 | AK103552 // Oryza sativa Japonica Group cDNA clone:J033132F15, full insert seque | 0.00076461 | 0.2909 |
| Os.56145.1.S1_at | 21.1439 | LOC_Os12g13890.1 // retrotransposon protein, putative, unclassified, expressed / | 0.00947863 | 0.3433 |
| Os.6043.1.S1_at | 21.4601 | LOC_Os04g23550.1 // basic helix-loop-helix family protein, putative, expressed / | 0.0158485 | 0.1567 |
| OsAffx.5150.1.S1_at | 23.1246 | LOC_Os06g48870.1 // methyl-binding domain protein MBD, putative, expressed // rg | 0.000816359 | 0.03316 |
| Os.11546.1.S1_at | 23.254 | AK121950 // Oryza sativa Japonica Group cDNA clone:J033107J23, full insert seque | 0.00139667 | 0.3124 |
| Os.12032.1.S1_at | 25.9804 | AB190817 // Oryza sativa Japonica Group OsWRKY71 mRNA for transcription factor O | 0.00121246 | 0.1489 |
| Os.38812.1.S1_at | 27.3487 | AY878695 // Oryza sativa (japonica cultivar-group) subitilisin-chymotrypsin inhi | 0.00116763 | 0.2699 |
| Os.3729.1.S1_at | 27.3618 | AB059238 // Oryza sativa IAI2 mRNA for wound induced protein homolog, complete c | 0.00210236 | 0.1843 |
| OsAffx.26226.1.S1_at | 33.6078 | BGIOSSBCE025115.1 // --- // bgi_ris // 0 // chr4:17209023-17209597(+) | 0.0000769 | 0.0129 |
| Os.4683.1.S1_at | 34.836 | AK100678 // Oryza sativa Japonica Group cDNA clone:J023113E15, full insert seque | 0.0335633 | 0.2286 |
| Os.49879.1.S1_at | 37.4229 | AK119268 // Oryza sativa Japonica Group cDNA clone:001-130-A08, full insert sequ | 0.00000309 | 0.004143 |
| Os.37571.1.S1_at | 37.7854 | CT829833 // Oryza sativa (indica cultivar-group) cDNA clone:OSIGCSN040B09, full | 0.000377245 | 0.2678 |
| Os.2915.1.S1_at | 49.1707 | AK243113 // Oryza sativa Japonica Group cDNA, clone: J100027C20, full insert seq | 0.0000535 | 0.2447 |
| Os.7593.1.S1_at | 53.8381 | LOC_Os08g12410.1 // pectinesterase, putative, expressed // rgap // 11 // --- /// | 0.0000321 | 0.02124 |
| Os.47438.1.A1_at | 79.3436 | BGIOSSBCE030523.1 // --- // bgi_ris // 0 // chr10:20479763-20480549(+) /// LOC_O | 0.000903852 | 0.139 |
