# Supplementary material for: Hydrostatic pressure-induced heritable variation in rice is associated with transposon mobilization, DNA methylation changes, and transcriptional reprogramming
Source: Plant Cell Rep. 2026 Oct 6;45(11):322. doi: 10.1007/s00299-026-04009-y (PMC13642388; doi:10.1007/s00299-026-04009-y)
Supplement: Supplementary file 2 [file 299_2026_4009_MOESM3_ESM.docx]

Supplementary Figure S1. Cytosine methylation patterns in promoter regions of four representative genes analyzed by bisulfite sequencing. DNA methylation profiles of LOC_Os03g08320, LOC_Os06g48870, LOC_Os02g48770, and LOC_Os07g48710 were visualized using CyMATE. Methylation status at CG, CHG, and CHH sites is shown for WT and HPM plants. Filled circles indicate methylated cytosines, whereas open circles indicate unmethylated cytosines.
